# Mixed Effects Deep Learning for the interpretable analysis of single cell RNA sequencing data by quantifying and visualizing batch effects

**Published:** 2024-11-13

**Authors:** Aixa X. Andrade, Son Nguyen, Albert Montillo

**Affiliations:** Lyda Hill Department of Bioinformatics, University of Texas Southwestern Medical Center, Dallas, TX 75390, USA

## Abstract

Single-cell RNA sequencing (scRNA-seq) data are often confounded by technical or biological batch effects. Existing deep learning models mitigate these effects but often discard batch-specific information, potentially losing valuable biological insights. We propose a Mixed Effects Deep Learning (MEDL) autoencoder framework that separately models batch-invariant (fixed effects) and batch-specific (random effects) components. By decoupling batch-invariant biological states from batch variations, our framework integrates both into predictive models. Our approach also generates 2D visualizations of how the same cell appears across batches, enhancing interpretability. Retaining both fixed and random effect latent spaces improves classification accuracy.

We applied our framework to three datasets spanning the cardiovascular system (Healthy Heart), Autism Spectrum Disorder (ASD), and Acute Myeloid Leukemia (AML). With 147 batches in the Healthy Heart dataset—far exceeding typical numbers—we tested our framework’s ability to handle many batches. In the ASD dataset, our approach captured donor heterogeneity between autistic and healthy individuals. In the AML dataset, it distinguished donor heterogeneity despite missing cell types and diseased donors exhibiting both healthy and malignant cells. These results highlight our framework’s ability to characterize fixed and random effects, enhance batch effect visualization, and improve prediction accuracy across diverse datasets.

## 1. Introduction

Single-cell RNA sequencing provides critical insights of cellular heterogeneity by capturing gene expression variations at single-cell resolution (Hwang et al., 2018), and it has become a widely used for analyzing biological systems. However, the non-linearity (Yousuff et al., 2024), sparsity (Bouland et al., 2023), and high dimensionality (Wu & Zhang, 2020) of scRNA-seq data pose challenges in extracting meaningful biological insights. Additionally, scRNA-seq data are often confounded by batch effects—variations introduced by different experimental conditions or biological variability (Tung et al., 2017).

These batch effects can arise from biological sources, such as donor variability, or technical factors, including collection time, library preparation methods, sequencing lanes, and differences during data integration from various experiments or laboratories (Haghverdi et al., 2018; Luecken & Theis, 2019). Such batch effects hinder researchers’ ability to uncover biological relationships within the data such as such as identifying cell types, mapping cell trajectories, and understanding gene expression patterns.

The increasing availability of publicly accessible scRNA-seq data has intensified the demand for effective data integration across multiple batches (Hwang et al., 2024). Consequently, batch correction algorithms have gained popularity, addressing this need. Autoencoder-based deep learning methods for batch correction have become prevalent due to their ability to handle non-linearity, as well as their flexibility, and scalability. These methods offer a robust approach to transforming data into a batch-invariant space. Pioneering examples include Batch Effect ReMoval Using Deep Autoencoders (BERMUDA) (Wang et al., 2019), DESC (Li et al., 2020), variational autoencoders for single-cell gene expression data (scVAE) (Gronbech et al., 2020), and autoencoders with cell type classifiers to preserve cell type signals like AutoClass (Li et al., 2020). On the other hand, the principles of generative adversarial networks (GANs)—where a generator aims to create batch-invariant data that appears realistic, and a discriminator strives to distinguish real from fake data—have also been employed for batch correction. Moreover, GANs have been combined with autoencoders and Mutual Nearest Neighbor (MNN) pairing for enhanced batch correction. For example, iMAP integrates all cells as if they originated from an anchor batch using an adversarial paired transfer network within the GAN framework, leveraging MNN pairing to align datasets (Wang et al., 2021). ResPAN advances this approach by introducing a residual autoencoder with skip connections and utilizing MNN pairing to guide the adversarial network across all batches without relying on an anchor (Wang et al., 2022). Building upon these adversarial frameworks, recent methods have incorporated batch classifiers and cell type classifiers to the adversarial autoencoders to further improve batch correction. scDREAMER integrates a batch classifier and offers the option of adding a cell type classifier for supervised batch correction, enhancing the preservation of biological variability while removing batch effects (Shree et al., 2023). Similarly, Integrating Multiple Single-Cell Datasets via an Adversarial Autoencoder (IMAAE) employs an anchor batch GAN-style adversarial approach combined with a cell type classifier to effectively align datasets (Wang et al., 2023). The Autoencoder-based Batch Correction (ABC) method introduces two cell type classifiers: one applied to the latent space and another to the reconstructed data. (Danino et al., 2024). Additionally, the Dynamic Batching Adversarial Autoencoder (DB-AAE) presents a novel strategy by varying the batch size during training—distinct from experimental or RNA-seq batch sizes—to enhance the model’s robustness to batch effects (Ko & Sartorelli, 2024). None of these methods explicitly model the batch effects and trying to correct the batch effects without explicitly modeling them can inadvertently discard crucial batch-specific information, especially when batches represent individual donors with biologically relevant data. This scenario is analogous to using models that include only fixed effects (Nguyen et al., 2023), which are inadequate when batch effects—akin to random effects in linear mixed-effects models—are present. Fixed-effects models concentrate solely on common signals, ignoring variability across batches or donors. We hypothesize that characterizing batch variability is important to enhance our understanding of scRNA-seq data and to make more accurate predictions of relevant biological states such as diagnoses and cell type. For example, when batch variability is experimental or technical, it can provide insights into experimental differences; when it is biological, it can reveal information about cellular heterogeneity. By precisely modeling and quantifying batch variability such methods can offer deeper biological insights and improve the interpretability.

Furthermore, Adversarial Information Factorization (AIF) addresses batch effect correction by explicitly modeling the distributions of cells conditioned on their respective batches. By learning these batch-specific distributions, AIF projects all cells onto a unified batch distribution, effectively aligning and integrating datasets across different batches (Monnier & Cournede, 2024). Furthermore, models that learn batch variability, such as AIF, *do not clearly separate batch-specific effects from dataset-invariant effects, reducing interpretability.* This lack of interpretability limits their applicability and misses opportunities in personalized medicine.

While numerous batch correction methods have been developed, most focus on removing batch effects without explicitly modeling them, *which can lead to overcorrection or loss of biologically relevant information—especially when biological signals are intertwined with batch effects* (Luecken et al., 2022). Some approaches attempt to enhance cell-type signals by incorporating cell-type labels into the integration process such as ABC or AutoClass by imposing similarity constraints (e.g., ResPAN and AIF). However, balancing effective batch correction with the retention of biological variation remains a challenge.

We propose the Mixed Effects Deep Learning (MEDL) framework builds upon subnetworks inspired by Nguyen et al. (2023), which models fixed effects and explicitly captures batch effects as random effects. Unlike previous methods that discard batch-specific information, MEDL retains both batch-invariant and batch-specific data, minimizing information loss. Implemented as an autoencoder tailored for scRNA-seq data, MEDL captures complementary information: fixed effects represent batch-invariant biological states, while random effects capture batch or donor-specific variations.

When batch effects are purely technical, the fixed effects subnetwork preserves cell type signals. However, when batches contain biological variation—such as tissue differences or donor-specific characteristics—the fixed effects subnetwork may inadvertently remove important biological information. In these cases, the random effects subnetwork becomes crucial by capturing the discarded batch-specific variations, allowing MEDL to recover meaningful signals that might otherwise be lost. By modeling these effects separately, MEDL provides a transparent and interpretable approach that preserves both core biological signals and batch-specific information, enhancing the accuracy of scRNA-seq analysis.

This approach not only improves classification of disease status, donor group, and cell type but also enhances interpretability by revealing the impact of batch-specific variations on cellular heterogeneity. Moreover, because our model trains the fixed and random effects subnetworks independently, it remains adaptable; existing batch correction methods that aim to generate a batch-invariant latent space such as AutoClass, ABC and scDREAMER—can be integrated into our framework as the fixed effects subnetwork, complemented by our random effects subnetwork.

We characterize our MEDL subnetworks through the following experiments: (1) We evaluate the model’s ability to preserve biological signals and correct batch effects within the fixed effects latent space while modeling batch effects in the random effects latent space. By utilizing the learned batch effects, we can compare differences in a cell’s scRNA dataset across batches via visual representation. (2) We demonstrate the complementary nature of the fixed and random effects subnetworks by using both latent spaces to predict targets of interest such as disease status, donor group, and cell type. (3) Inspired by models like ABC, scDREAMER, and AutoClass, we incorporate a cell-type classifier into the fixed effects subnetwork to evaluate its impact on cell-type signal preservation and batch correction, mitigating potential loss of cell type information.

## 2. Materials

To create a comprehensive evaluation of our MEDL framework across diverse biological and technical contexts, we selected three datasets that span key dimensions of variability in scRNA-seq data (Table 1). These dimensions include health status (healthy individuals versus diseased patients), tissue types (heart, brain, blood cells), sources of batch effects (technical protocols, donor differences, tissue types, inclusion of cell lines), and the degree of confoundedness between cell type and batch.

The first dataset, the *Healthy Heart dataset* (Litvinukova et al., 2020; Yu et al., 2023a) consists of scRNA-seq data from heart tissues of healthy individuals. It presents multiple sources of batch variability due to the use of two different sequencing protocols (technical differences), samples from various donors, and different tissues within the heart. There is confoundedness between cell type and batch because some cell types are present only in specific tissues. This dataset allows us to assess MEDL’s ability to handle a high number of batches and complex batch effects.

The second dataset, the *Autism Spectrum Disorder (ASD)* (Velmeshev et al., 2019) includes single-nuclei (sn) RNA-seq data from prefrontal cortex (PFC) and anterior cingulate cortex (ACC) brain samples. Batch effects primarily arise from donor variability between ASD and control subjects. There is indirect confoundedness between cell type and batch, as certain cell types are more affected in ASD than in controls. This dataset enables us to evaluate MEDL’s capacity to capture donor heterogeneity and disease-related biological variations.

The third dataset, the *Acute Myeloid Leukemia (AML)* (van Galen et al., 2019) comprises scRNA-seq data from healthy individuals, AML patients, as well as cell lines. Batch effects stem from donor variability, including differences between healthy and AML subjects, and cell lines. This dataset features strong confoundedness between cell type and batch: malignant cells that are only present in diseased subjects and cell lines, while healthy cells can originate from both diseased and healthy patients.

### Data Preparation and Preprocessing

All the scRNA-seq data was preprocessed with the Scanpy library (Wolf et al., 2018) using standard preprocessing steps (Yu et al., 2023a).

The datasets were then divided into five folds for cross-validation, stratified by batch and the biological feature of interest—cell type in all three datasets. For each split, three folds were used for training, one for validation, and one for testing. The data were loaded for each subset and scaled between zero and one using min-max scaling before model fitting.

Since the ASD and AML datasets had previously undergone quality control, no additional cells were removed during the filtering step. For the AML dataset, additional quality control measures were implemented. Cells with undefined or missing cell type annotations were excluded. Furthermore, samples identified by Dai et al., (2021) as having ambiguous annotations—specifically AML314, AML371, AML722B, and AML997 (Dai et al., 2021)—were removed from the original 16 AML donors to ensure the dataset’s integrity, resulting in 12 AML donors available for analysis. The ASD dataset had previously undergone a log2 transformation as part of the original preprocessing by van Galen et al. (2019). To maintain consistency across all datasets, this transformation was reversed by applying an exponential function (base 2) to the data after adding 1 to each value, thereby returning the data to its original scale. Following this reversal, the standard preprocessing pipeline was applied.

## 3. Methods

### 3.1. MEDL Framework for scRNA-seq data

When data is confounded by batch effects, as commonly observed in single-cell RNA sequencing (scRNA-seq) data, it is essential to employ methods that account for these confounders. The Mixed Effects Deep Learning (MEDL) framework addresses this need by extending linear mixed-effects models (Vestal et al., 2022) into a nonlinear context. A linear mixed-effects model is defined as:

$$y_{i}=x_{i}^{T}\beta_{i}+x_{i}^{T}u_{j}+\epsilon_{i}$$

In this equation, $y_{i}$ represents the target variable, $x_{i}^{T}$ is the gene expression vector of the $i^{th}$ cell, $\beta_{i}$ denotes the fixed effects intercepts and weights capturing batch invariant trends across all samples, $u_{j}$ signifies the random effects intercepts and weights accounting for variability of the batches j (assumed to follow a normal distribution $N\left(0,\sigma_{j}\right)$), and $\epsilon_{i}$ is the residual error term. Building upon this foundation, the MEDL framework extends the model into a nonlinear context, effectively addressing the inherent non-linearity from confounded scRNA-seq data.

#### MEDL Model Architecture

Our Mixed Effects Deep Learning Autoencoder (MEDL-AE) framework for single-cell RNA sequencing (scRNA-seq) data captures both fixed and random effects within gene expression vectors of individual cells. Fixed effects represent batch invariant features, while random effects account for batch or donor-specific variations. The model processes a gene expression count matrix $X\in R^{n\times m}$, where n is the number of cells and m is the number of genes, the model outputs a gene expression matrix $\overset{ˆ}{X}$ and latent space representations derived from the encoder.

**Fig. 1.**
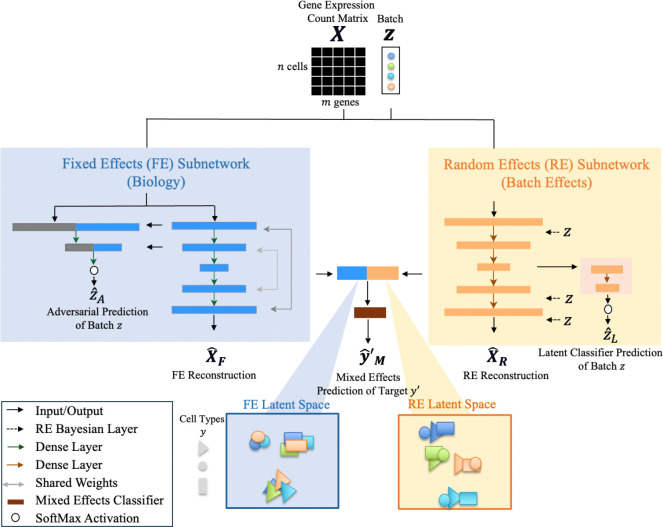
Architecture of the Mixed Effects Deep Learning Autoencoder (MEDL-AE) for scRNA-Seq data. The inputs to the model are the gene expression count matrix X and the one-hot encoded batch design matrix z. Both subnetworks aim to reconstruct the input data, outputting a reconstructed gene expression matrix $\hat{X}$. The blue block represents the *Fixed Effects subnetwork (MEDL-AE-FE)*, which comprises a conventional autoencoder augmented with an adversarial classifier. This classifier penalizes the model’s ability to predict batch labels z from the latent representation, encouraging the encoder to learn a batch-invariant latent space that preserves the cell type signal y. The orange block represents the *Random Effects subnetwork (MEDL-AE-RE)*, featuring an autoencoder with dense layers and additional random effects layers designed to capture batch-specific variations. This subnetwork enhances batch variability in its latent representation by modeling the random effects associated with different batches. For each subnetwork, the encoder produces a latent space representation. The fixed effects latent space is batch-invariant and preserves cell type information y, while the random effects latent space captures and enhances batch-specific variability. A classifier, depicted in brown, can be applied to the latent spaces to predict the labels of interest ${\overset{ˆ}{y}}^{\prime }$. This allows us to assess how well each latent space representation preserves the biological signals necessary for accurate cell type classification.

The model architecture of our framework comprises two parallel subnetworks: one which captures the fixed effects and another for the random effects (Fig. 1). The fixed effects subnetwork (MEDL-AE-FE) is designed to suppress batch effects. It consists of an autoencoder (AE) with weight tying between encoder and decoder, two dense hidden layers, and an adversarial classifier that learns to predict the batch labels 𝑧.

The fixed effects loss function $L_{FE}$ is given by:

$$L_{FE}(X,\overset{ˆ}{X},z,\overset{ˆ}{z}):\lambda_{MSE}L_{MSE}(X,\overset{ˆ}{X})-\lambda_{A}L_{CCE}(z,\overset{ˆ}{z})$$

Here, $\lambda_{\text{MSE}}$ and $\lambda_{A}$ balance the reconstruction and adversarial losses, respectively. The mean squared error (MSE) measures the reconstruction error:

$$L_{MSE}(X,X)=\frac{1}{n}\sum_{i=1}^{n}\;{\left||X_{i}-{\overset{ˆ}{X}}_{i}|\right|}^{2}$$

where $X_{i}$ and ${\overset{ˆ}{X}}_{i}$ are the original and reconstructed gene expression vectors of cell i. The categorical cross-entropy (CCE) quantifies the adversarial classifier’s performance:

$$L_{CCE}(z,\overset{ˆ}{z})=-\frac{1}{n}\sum_{i=1}^{n}\;\sum_{k=1}^{K}\;z_{i,k}\;log\;{\overset{ˆ}{z}}_{i,k}$$

where $z_{i,k}$ is the true label (one-hot encoded) and ${\overset{ˆ}{z}}_{i,k}$ is the predicted probability for class k for cell i. Optimizing $L_{FE}$ enables the model to effectively capture fixed effects while mitigating batch-specific variations.

The random effects subnetwork models the distribution of batch-specific variations using variational inference (Blei et al., 2017). The true batch distributions p(U∣X) with $U=\left\{u_{j}\right\}$ are approximated by an optimized surrogate posterior q(U). Each batch prior p(U) is represented as a normal distribution centered at zero.

Although the objective involves *maximizing* the Evidence Lower Bound (ELBO), in practice, we can achieve this by *minimizing* the negative ELBO. This leads to the following loss function:

$$L_{ELBO}=-E_{q(U)}[log\;p(X|U)]+D_{KL}(q(U)∥p(U))$$

Maximizing the ELBO is equivalent, up to an additive constant logp(X), to minimizing the Kullback–Leibler (KL) divergence between the surrogate posterior (q(U) and the true posterior p(U∣X).


$$D_{KL}(q(U)∥p(U|X))=log\;p(X)-ELBO$$

The expected log-likelihood $E_{q(U)}[log\;p(X|U)]$ measures how well the model fits the data. This term is approximated using suitable loss functions—in this case, a combination of MSE for data reconstruction and CCE for batch classification:

$$-E_{q(U)}[log\;p(X|U)]\approx \lambda_{RE_{1}}L_{MSE}\left(X,{\overset{ˆ}{X}}^{\prime }\right)+\lambda_{RE_{2}}L_{CCE}(z,{\overset{ˆ}{z}}^{\prime })$$

Here, $\overset{ˆ}{X}$ is the reconstructed gene expression matrix, and ${\overset{ˆ}{z}}^{\prime }$ represents the predicted batch labels from the random effects subnetwork. The term logp(X) is constant with respect to U and can therefore be ignored during optimization.

Combining these components, the total loss function for the random effects subnetwork $L_{RE}$ becomes:

$$L_{RE}\left(X,\overset{ˆ}{X^{\prime }},z,{\overset{ˆ}{z}}^{\prime },\right)=\lambda_{MSE}L_{MSE}\left(X,\overset{ˆ}{X^{\prime }}\right)+\lambda_{CCE_{z}}L_{CCE}\left(z,{\overset{ˆ}{z}}^{\prime }\right)+\lambda_{KL}D_{KL}(q(U)∥p(U))$$

where $\lambda_{MSE},\;\lambda_{CCE_{z}}$ and $\lambda_{KL}$ are weighting factors for each loss term. The first term encourages accurate reconstruction of the data, the second term ensures that the latent space captures batch-specific information, and the third term regularizes the model by aligning the learned distribution q(U) with the prior p(U), thus preventing overfitting.

The Kullback–Leibler (KL) divergence between two univariate Gaussian distributions $q\left(u_{j}\right)=N\left(\mu_{q},\sigma_{q}^{2}\right)$ and $p\left(u_{j}\right)=N\left(\mu_{0},\sigma_{0}^{2}\right)$ is given by:

$$D_{KL}\left(q\left(u_{j}\right)∥p\left(u_{j}\right)\right)=\int_{-\infty }^{\infty }\;q(u)\;log\frac{q\left(u_{j}\right)}{p\left(u_{j}\right)}\;du$$

In a more explicit form, the KL divergence over all batches 𝑗 can be written as:

$$D_{KL}(q(U)∥p(U))=\frac{1}{2}\sum_{j}\;\left[log\left(\frac{\sigma_{j}^{2}}{\sigma_{0}^{2}}\right)-1+\frac{\sigma_{0}^{2}\;+\;{\left(\mu_{j}\;-\;\mu_{0}\right)}^{2}}{\sigma_{j}^{2}}\right]$$

where $\mu_{j}$ and $\sigma_{j}^{2}$ are the mean and variance of $q\left(u_{j}\right)$, and $\mu_{0}=0$ and $\sigma_{0}^{2}$ are those of the prior $p\left(u_{j}\right)$.

The MEDL hyperparameters are described in Tables S1–S3. In the fixed effects subnetwork, we employed two Adam optimizers: one dedicated to training the adversarial classifier alone, and the other optimizing the total loss of the subnetwork. In contrast, the random effects subnetwork was trained using a single Adam optimizer. Early stopping based on validation loss was utilized throughout as a regularization strategy to prevent overfitting.

#### Comparable, Complementary and Auxiliary Models

To rigorously evaluate the performance of the MEDL models, we incorporated comparable models that are widely used in scRNA-seq analysis, as well as auxiliary models to further validate our approach. Principal Component Analysis (PCA), a standard method for dimensionality reduction in scRNA-seq, was implemented using scikit-learn (Pedregosa et al., 2015) and served as a baseline for comparison. While PCA provides linear dimensionality reduction, it does not explicitly address batch effect correction, making it a useful reference point. To overcome PCA’s linearity limitation, we included a standard Autoencoder (AE) as a nonlinear alternative. This model served as both an ablation test for the MEDL-AE-FE model and as a complementary nonlinear dimensionality reduction method to PCA.

To evaluate the complementary nature of the two subnetworks (fixed and random effects), we employed a Random Forest classifier (Breiman, 2001) from scikit-learn with default hyperparameters (Pedregosa et al., 2015) to classify the target labels based on the latent representations from the fixed effects subnetwork (MEDL-AE-FE) and the combined latent spaces from the full MEDL-AE model. The Random Forest model was chosen because it is interpretable, effectively handles both linear and nonlinear relationships and it is not prone to overfitting. We used the same 5-fold cross-validation partitioning scheme described for preprocessing the data input for the MEDL subnetworks. The PCA latent space was also utilized as a comparative baseline. This approach enabled us to assess how the integration of both fixed and random effects enhances classification performance compared to using fixed effects alone.

Furthermore, when cell-type labels are available or can be estimated for certain datasets, an approach to leveraging this information is to use an Autoencoder Classifier (AEC). To ensure a comprehensive evaluation, we included this model in our comparisons.

The MEDL Autoencoder (AE) model can be complemented with a latent classifier to enhance the cell-type signal, thereby forming the fixed effects classifier subnetwork (MEDL-AEC-FE). This subnetwork predicts the one-hot encoded labels y by incorporating a categorical cross-entropy term $L_{CCE}(y,\overset{ˆ}{y})$ into the fixed Effects loss, weighted by $\lambda_{CCE_{y}}$. The updated fixed effects loss function $L_{FE}$ becomes:

$$L_{FE}(X,\overset{ˆ}{X},z,\overset{ˆ}{z},y,\overset{ˆ}{y})=\lambda_{MSE}L_{MSE}(X,\overset{ˆ}{X})-\lambda_{A}L_{CCE}(z,\overset{ˆ}{z})+\lambda_{CCEy}L_{CCE}(y,\overset{ˆ}{y})$$

Additionally, an Autoencoder Classifier (AEC) with an architecture similar to AutoClass (Li et al., 2022) was included as an ablation test for the MEDL-AEC-FE model. This facilitated the evaluation of the specific contribution of the classifier to cell type signal preservation and batch correction.

### 3.2. Performance Metrics

The core metric used to guide our MEDL training was the validation total loss for each of the subnetworks which is described in equations 1 and 2. Additionally, clustering metrics were used to evaluate the biological separability, batch correction, and batch modeling.

#### Metrics to characterize cell type separability, batch correction, and batch modeling

To assess the effects of the MEDL subnetworks on cell type separability, batch correction, and batch modeling, we utilized three internal clustering metrics: Average Silhouette Width (ASW) (Rousseeuw, 1987), Calinski-Harabasz (CH) index (Calinski & Harabasz, 1974), and Davies-Bouldin (DB) index (Davies & Bouldin, 1979), to assess the effects of the MEDL subnetworks on cell type separability, batch correction, and batch modeling. These metrics have been widely used in scRNA-seq data analysis (Li et al., 2021; Ning et al., 2023; Wang et al., 2024), with ASW being the most popular among them (Hu et al., 2024; Luecken et al., 2022; Shree et al., 2023; Yu et al., 2022; Yu et al., 2023a).

The average silhouette width (ASW) is defined as the mean of the silhouette widths for all data points in the dataset. We selected ASW as our primary metric for its popularity, its robustness to clusters of varying shapes compared to the Davies-Bouldin (DB) and Calinski-Harabasz (CH) indices (Vendramin et al., 2010), and because its values range from −1 to 1, facilitating comparisons across methods and datasets. Unlike the CH and DB indices—which assume convex or spherical clusters and focus on cluster variance structure and overlap, respectively—ASW does not rely on such assumptions. By including the CH and DB indices, we ensured a comprehensive evaluation from different perspectives: CH captures variance structure and compactness, while DB highlights the degree of overlap between clusters.

We computed these scores using both cell type and batch labels for cell samples, capped at 10,000 per fold (training, validation, or test). Biological separability was measured using cell type labels from the original publications: ASD dataset (Velmeshev et al., 2019), Healthy Heart dataset (Yu et al., 2023b), and AML dataset (van Galen et al., 2019).

To align the interpretation of the DB index with the CH index and ASW, we used its reciprocal (1/DB), ensuring that higher scores consistently indicate better clustering quality. For cell types, higher scores signify improved separability in the latent space, enhancing the cell type signal—the primary objective of batch correction. Conversely, lower scores for batches indicate more effective batch correction, as batches become less distinguishable, reflecting improved batch mixing. Additionally, higher batch scores in the random effects subnetwork suggest effective capture of between-batch variance in gene expression data.

#### Classification Performance Metrics

To evaluate the classification performance of predicting labels from the MEDL latent spaces, we calculated accuracy, balanced accuracy, and chance accuracy. For chance accuracy, we employed scikit-learn’s Dummy Classifier with the stratified strategy, which generates random predictions while maintaining the original class distribution in the dataset (Pedregosa et al., 2015). This provides a more realistic baseline for comparison, as it accounts for the imbalanced nature of the classes rather than assuming uniform distribution, thus offering a more appropriate measure of chance-level performance. These metrics range between 0 and 100%. Balanced accuracy accounts for class imbalance, which is crucial in our datasets due to multiple cell type classes with varying abundances.

In the results section, we report test results for ASW, classification accuracy, balanced accuracy and chance accuracy.

### 3.3. Hyperparameter Optimization

The MEDL model comprises two subnetworks—the fixed effects subnetwork and the random effects subnetwork—each with multiple hyperparameters detailed in Tables S1–S3. All hyperparameters, except for the weights assigned to individual loss terms, were kept constant across all base models and ablation tests. The total loss function is constructed as a sum (or difference) of individual loss terms, each multiplied by its specific weight hyperparameter, thereby controlling the contribution of each loss term to the overall loss function.

To select the weights of the individual loss terms, we employed a manual, case-by-case strategy using training and validation folds from the 5-fold cross-validation detailed in the preprocessing section. Our primary objective was to ensure proper optimization and prevent any single component—such as the adversarial loss—from dominating the training process. Specifically, we ensured that the reconstruction loss remained of the same order of magnitude, or greater, than the other loss terms. This involved running the model for several epochs and monitoring the contribution of each loss term to the total loss function. Adjustments were made to the weights so that none of the adversarial, cell type classifier, batch classifier, or KLD losses exceeded the reconstruction loss during the initial epochs of training. By maintaining this balance, we facilitated proper optimization across all components.

Due to the variation in the number of batches across datasets, the loss weights for the adversarial and reconstruction components were adjusted accordingly, as illustrated by the training curves (Fig. S5–S10). For the ASD and AML fixed effects subnetworks, we performed additional hyperparameter optimization to maximize cell type separability in the latent spaces using the ASW for cell type. Using our manual, case-by-case approach, we increased the reconstruction loss weight relative to the adversarial loss whenever the cell type signal diminished.

Early stopping based on validation loss was utilized throughout as a regularization strategy to prevent overfitting. In summary, our hyperparameter selection involved carefully balancing the weights of the loss terms, using training and validation folds from the 5-fold cross-validation strategy, and applying early stopping to ensure robust and generalizable models.

For evaluation and visualization purposes, the latent space of all models was encoded into a two-dimensional vector for each data cell, resulting in an (n,2) matrix where n is the number of cells. This two-dimensional latent representation allows us to effectively visualize and evaluate the model’s performance.

### 3.4. Visualization Methods

We used Uniform Manifold Approximation and Projection (UMAP) (McInnes et al., 2018) visualizations of the models latent spaces to provide a more comprehensive evaluation of the model’s performance. This combined approach allows us to better assess both cell-type signal enhancement and batch mixing. We generated UMAP visualizations using Scanpy UMAP tools (Wolf et al., 2018).

#### Genomaps for 2D visualization of individual cells

Genomaps (Islam & Xing, 2023) provide a 2D representation of gene expression profiles for each cell by arranging genes on a 2D grid according to their gene-to-gene interactions. In this layout, genes positioned at the same distance r from the center exhibit equivalent levels of gene-to-gene interaction. Importantly, this method preserves the original gene expression values while organizing the genes spatially, allowing for a clear visualization of the relationships between genes and providing insights into batch effects. Islam and Xing et al. (2023) demonstrated that Genomaps can create gene expression patterns specific to cell types. We visualized batch variability as Genomaps specific patterns.

### 3.5. Experiments

#### 3.5.1 Experiment 1: Characterization of the cell type separability, batch correction and visualization of learned batch effects

We conducted two analyses to understand the performance and interpretability of the proposed MEDL framework. First, we assessed the latent spaces of the two MEDL subnetworks quantitatively and qualitatively by (a) evaluating cell type separability, batch correction, and (b) visualizing the learned batch effects through gene expression reconstructions called genomaps (Islam & Xing, 2023), which provided a 2D image representations of the gene expression profile individual cells.

##### a) Characterization of cell type separability and batch correction of each MEDL subnetwork

To demonstrate that the fixed effects subnetwork (MEDL-AE-FE) and the random effects subnetwork (MEDL-AE-RE) learn complementary information, we assessed their ability to transform input sequence data into batch-invariant and batch-specific latent spaces, respectively. The fixed effects subnetwork is designed to map the input to a batch-invariant space, which can enhance cell type separability as cell type information is a larger proportion of the information contained in the batch-invariant space. We evaluated this by calculating the Average Silhouette Width (ASW) across the cell types in the latent space derived from its encoder, where a higher ASW indicates better separability of the cell types. We also measured the degree of batch correction in the fixed effects subnetwork by calculating the ASW across the batches, where a lower ASW indicates more effective suppression of batch effects (i.e., less batch separability). Conversely, in the random effects subnetwork, an increase in the ASW for batch suggests successful capture of batch-specific effects. In this work, latent spaces were obtained from the bottleneck layer (i.e., the encoder outputs) of each subnetwork.

To ensure robust estimates, we employed 5-fold cross-validation to calculate the mean ASW and calculated the 95% confidence intervals across folds. To reduce compute time for the ASW calculations, we used a random sample of 10,000 cells when datasets exceeded this size in training, validation, or testing subsets. For baseline comparisons, we also computed these characteristics of the latent spaces from a Principal Component Analysis (PCA) based approach and from a standard Autoencoder (AE) model.

##### b) Visualization of learned batch effects

To demonstrate the unique capabilities of our Mixed Effects Deep Learning Autoencoder (MEDL-AE) framework to address critical biological and clinical “what if” questions—an advantage not offered by traditional methods—we visualized the gene expression patterns learned by the Fixed Effects (FE) and Random Effects (RE) subnetworks under different conditions. Specifically, we reconstructed gene expression count matrices using both subnetworks. The FE subnetwork transforms input data into a batch-invariant space, revealing gene expression patterns common across all batches. In contrast, the RE subnetwork captures batch-specific variations, allowing us to simulate how gene expression profiles would appear if cells originated from a different batch, i.e., a different technical batch or biological donor.

By setting the one-hot encoded batch vectors in the RE subnetwork, we projected cells into different technical batches conditions, or biological batches (e.g., donor or donor diagnoses). This projection capability enabled us to explore variability between different donors, or diagnoses, or technical data acquisition conditions. This enables new capacity to answer pertinent biological questions such as how gene expression changes when a cell is subjected to different technical batch effects, changes in the donor, or changes in the disease diagnosis. For example, we can now address questions like: What would this cell have looked like if it had instead come from a diseased donor?

To facilitate this exploration, we randomly selected 300 cells from specific cell types of interest, ensuring that each batch of interest was represented. We projected the gene expression data for these cells using the Autoencoder (AE), MEDL-AE-FE, and MEDL-AE-RE models. Using MEDL-AE-RE, we obtained counterfactual projections of every cell into all batches. We combined these projections with the original gene expression vectors to form an overall count matrix, which we standardized. From this matrix, we computed a genomap transform—a visualization that was unbiased toward any specific projection. By using the same genomap transform for all projections, we maintained a consistent arrangement of genes to pixels, enabling direct comparison of individual pixels (genes) across the genomaps. Finally, we plotted the batches of interest on the genomap to compare gene expression patterns across batches. This visualization highlighted the effects of batch correction, underlying biological signals, and batch-specific influences, enabling us to assess cell variability across batches.

For instance, in the Healthy Heart dataset, we focus on four cell types of interest—pericytes, endothelial cells, fibroblasts, and ventricular cardiomyocytes—across four different batches. By projecting cells into different batches using the RE subnetwork, we examine how the batch effects impact the gene expression profiles, and how these differences vary across cell types. In the ASD dataset, we investigate differences between autistic and healthy individuals by visualizing L2/3 excitatory neurons from three control donors and three autistic donors. We selected L2/3 excitatory neurons because they exhibit the highest number of differentially expressed genes between autistic and control subjects (Velmeshev et al., 2019). By projecting cells from healthy donors onto the autistic condition—and vice versa—we elucidate alterations in gene expression associated with autism. This can not only provide insight into disease-specific gene expression patterns but also demonstrate the model’s ability to visualize diagnostic conditions. Similarly, in the AML dataset, we visualize healthy and malignant monocytes from healthy donors, diseased donors, and cell lines. We utilize the RE subnetwork to project cells across different conditions to demonstrate how our MEDL-AE-RE model captures variations associated with disease states. This capability is crucial to help understand the gene expression changes underlying malignancy and to aid in identifying therapeutic targets. Overall, this experiment explores our frameworks projection capability, to allow researchers to simulate and visualize how gene expression profiles change under different conditions. In this way we explore how it can provide insights into biological variability and disease mechanisms, a unique capacity for answering biological and clinical questions that are not accessible through prior batch effects suppression methods.

#### 3.5.2 Experiment 2: Evaluation of how the complementary nature of the FE and RE latent representations enhances prediction performance

The aim of this experiment is to leverage the complementary nature of the Fixed Effects (FE) and Random Effects (RE) latent representations to enhance label prediction. The FE subnetwork (MEDL-AE-FE) suppresses batch effects by transforming gene expression data into a batch-invariant space, while the RE subnetwork (MEDL-AE-RE) constructs a space that captures batch effects, by explicitly modeling the batch-to-batch variability. This experiment explores the extent to which quantitatively modeling batch effects, rather than discarding them as other batch correction methods do, can improve models that make predictions about the cells, with prediction targets such as cell type, patient group, and disease diagnosis.

To assess the complementary nature of the fixed and random effects latent spaces, we utilize the latent representations from MEDL-AE-FE and MEDL-AE-RE obtained in Experiment 1. Then we train a Random Forest classifier using three different input sets: (1) the latent space from MEDL-AE-FE alone, (2) the latent space from the principal component analysis (PCA) baseline model, and (3) the concatenated latent spaces of MEDL-AE-FE and MEDL-AE-RE, which combines the fixed and random effects representations. Prior to training, all latent spaces were standardized, adjusting each to have a mean of zero and a standard deviation of one, to ensure comparability of the inputs.

Since all three datasets (van Galen et al., 2019; Velmeshev et al., 2019; Yu et al., 2023b) included cell type labels, we used cell type as the primary target for classification. In the AML dataset, malignant and healthy cells from the same cell type were treated as two distinct cell type labels. We conducted experiments using several targets. For the AML dataset we used patient group—which includes diseased (AML patients), healthy donors, and cell line classes—as the target variable to assess the model’s capability to differentiate among these groups. For the ASD dataset we used disease diagnosis (autism spectrum disorder or control) as the target variable. To ensure consistency in our evaluation, we employed the same 5-fold cross-validation splits as in Experiment 1 for all classification tasks.

#### 3.5.3 Experiment 3: Quantification of the impact of an embedded cell type classifier on batch and cell type separability

Many transcriptomics datasets include cell type labels from an external source. When such cell type labels are available, this information can be leveraged by an *autoencoder classifier* to further ensure preservation of the cell type information in its latent representation. The autoencoder classifier aims to construct a latent representation that simultaneously has low reconstruction error *and* high cell classification performance. Ultimately, we hypothesize that autoencoder *cell type classifier* will induce a trade-off between batch effects suppression and cell type separability. Therefore, in this experiment we replace the AE in our MEDL-AE of Experiment 1, with an autoencoder classifier for cell type in a MEDL-AEC model and investigate how it impacts batch effects suppression and cell type separability. For such a MEDL-AEC model, our FE model becomes a MEDL-AEC-FE model, while our RE model does not need to change, since its goal is to construct a latent space predictive of batch. We then not only evaluate the impact of the latent space classifier on cell type and batch separability, but also assess whether these effects are driven by the classifier itself or by the adversarial component. To do this, we compare the performance of MEDL-AEC-FE against a baseline AEC model. We use the same five-fold cross-validation partitioning splits of Experiment 1a, and we computed the mean ASW for batch and cell type, along with 95% confidence intervals, across the five folds.

### 3.6 Implementation

All deep learning models were developed using TensorFlow 2.3 (Martín Abadi et al., 2015) and trained on Nvidia Tesla V100 GPUs with 32 GB of memory and Tesla P4 GPUs with 8 GB of memory. However, the fixed effects subnetworks and autoencoder models were less computationally intensive and could be trained on simpler hardware, such as the Tesla P4 GPU with 8 GB of memory. We calculated the ASW, DB, CH, accuracy, balanced accuracy, and chance accuracy using the Scikit-learn package (Pedregosa et al., 2015). ScRNA-seq preprocessing and UMAP computations (McInnes et al., 2018) were performed using the Scanpy library (Wolf et al., 2018).

## 4. Results

The following sections present the results of the three experiments. For each experiment the results are presented for the Health Heart, ASD, and AML datasets.

### 4.1. Experiment 1. Characterization of cell type separability, batch correction and visualization of learned batch effects

#### a) Characterization of cell type separability and batch correction of each MEDL subnetwork

##### Healthy Heart dataset

Table 2 presents the mean Average Silhouette Width (ASW) scores for batch and cell type separability across five folds of the Healthy Heart dataset, comparing latent spaces generated by the PCA, AE, MEDL-AE-FE, and MEDL-AE-RE models. PCA is the baseline model, as it is often used in scRNA-seq analysis to reduce dimensionality. Similarly, the AE is included as a reference model, as it is also a commonly used approach for scRNA-seq dimensionality reduction. First, we compared the methods by separability. Here we observe that PCA provides a baseline ASW of −0.48, while the proposed MEDL framework both suppresses batch separability with the MEDL-AE-FE attaining −0.50 (more negative is less separable) and learns the batch effect with the MEDL-AE-RE attaining an ASW of +0.37 (Table 2 left columns) and forms clusters separable by batch (Fig. S2, Table S7). This confirms the power of the proposed approach to reduce dimensionality while both suppressing and modeling the batch effect, while other approaches such as the AE merely have one effect (dimensionality reduction). The MEDL-AE-FE (−0.50) also suppresses batch effects better than the AE (−0.45). Second, we compared the methods by cell type separability. Here, the PCA approach gives a baseline score of −0.05. We observe that both the AE and MED-AE-FE substantially improve cell type separability compared to the PCA (Table 2 right columns, and Table S7). Second, we compared the methods by cell type separability. Here, the PCA approach gives a baseline score of −0.05. We observe that both the AE and MED-AE-FE substantially improve cell type separability compared to the PCA (Table 2 right columns, and Table S7).

**Fig. 2.**
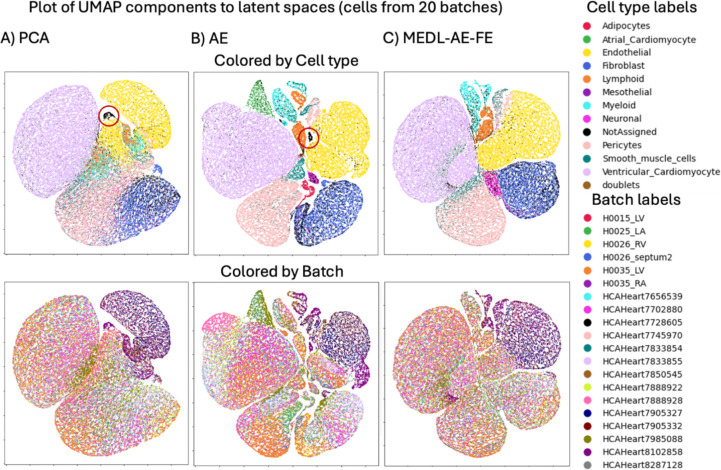
UMAP visualizations of 44,987 cells from 20 out of 147 batches in the latent space from the Healthy Heart dataset, generated from three different models: A) PCA, B) AE and C) MEDL-AE-FE.MEDL-AE-FE.

These trends are also evident in the latent space visualizations of these models (Fig. 2). In particular we apply UMAP (McInnes et al., 2018) to 44,987 cells from 20 batches selected randomly out of 147 batches, to improve figure clarity. In the first row, which contrasts cell-type separability, the AE and MEDL-AE-FE both show many discernable cell types, much more so than PCA, which confirms the increased cell type separability that these methods attain as quantified in Table 2. The second row which depicts the batch separability of the methods, we observe that the MEDL-AE-FE model has the most uniform distribution of batches (colors), while the AE has many discernable batches (colored regions), in agreement with Table 2.

**Fig. 3.**
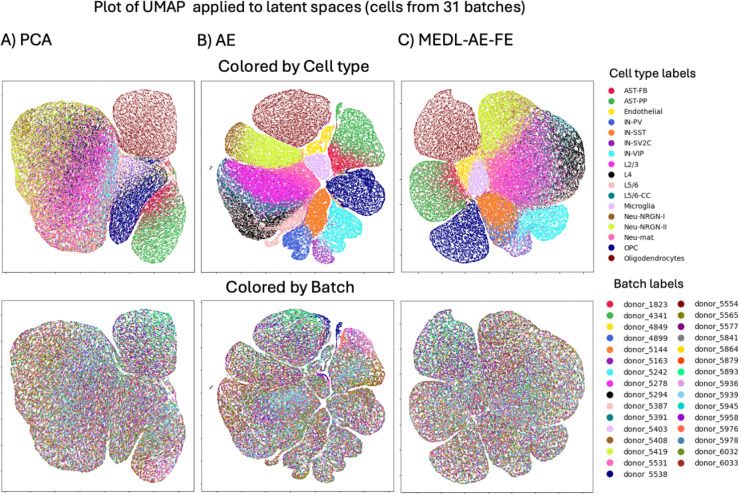
UMAPs from 62735 cells from ASD dataset latent spaces obtained with A) PCA (Raw), B) AE and C) MEDL-AE-FE.

Cells labeled as “Not Assigned” by Yu et al., 2023 (Yu et al., 2023b)—meaning they could not be assigned to any known singular cell type—are dispersed in the MEDL-AE-FE latent representation (Fig. 3C), reflecting their heterogeneity. In contrast, these unassigned cells form a small cluster (circled) in the AE and PCA representations (Fig. 2A and B), which is likely a false positive since they are not expected to cluster together. This observation indicates that while AE and PCA can amplify signal, they may also amplify noise. Additionally, when using the MEDL-AE-FE model, atrial cardiomyocytes are dispersed alongside ventricular cardiomyocytes. We attribute this to the suppression of batch effects—a combination of variations due to tissue type, donor, and protocol. By eliminating some of the tissue variability information, both types of cardiomyocytes appear to merge. By comparing the AE representation to the MEDL-AE-FE representation, we can identify which cell types are batch-invariant, thereby further enhancing our understanding of the cell variability.

##### ASD dataset

Next, we evaluated the proposed framework on the ASD dataset. Comparing the cell type separability, in this dataset, the MEDL-AE-FE latent space improved the cell type separability compared to PCA, as evidenced by more defined boundaries for myeloid, lymphoid, pericytes, and smooth muscle cells (Fig. 3, top row, column A and C). Although the ASW batch scores of MEDL-AE-FE are not more negative than those of PCA (Table 3, left columns), we observe that in the UMAP projection of Fig. 3 (bottom row), an equivalent degree of batch mixing is visibly apparent between the two methods, giving confidence that the framework has formed a FE space with suppressed batch effects. Additionally, we observe that the nonlinear properties of the AE component in MEDL-AE-FE best preserve the cell type signal (Table S5). The total loss is calculated as the reconstruction loss minus the adversarial loss. Therefore, increasing the weight of the reconstruction loss reduces the adversarial contribution to the total loss, which lowers batch correction but enhances the cell type signal. We selected the MEDL-AE-FE model with a slightly higher reconstruction loss because it exhibited the strongest cell type signal while effectively mitigating some batch effects (Table 3 and Fig. 3).

By comparing the latent spaces of the AE and the MEDL-AE-FE models on the ASD dataset (Fig. 3, columns B and C), we can identify neuron types that exhibit donor heterogeneity. For instance, parvalbumin interneurons—which Velmeshev et al., 2019 reported to be dysregulated in patients with epilepsy and ASD—form a distinct cluster in the AE latent space but become dispersed in the MEDL-AE-FE representation. This dispersion suggests that the MEDL-AE-FE model effectively removes donor-specific effects. Similarly, certain excitatory neurons, such as layer 2/3 (L2/3) and layer 5/6 corticocortical (L5/6CC) neurons—which are dysregulated in ASD compared to controls—also become dispersed among other layer neurons in the MEDL-AE-FE latent space. In contrast, oligodendrocytes and oligodendrocyte precursor cells (OPCs) exhibit minimal donor heterogeneity, indicating that their latent representations are largely unaffected by donor-specific variability. This distinction highlights how the proposed framework can be used to learn a donor invariant latent space and enhance our understanding of neurobiology.

The MEDL-AE-RE shows an increase in the ASW, 1/DB and CH batch scores compared to other models, confirming its ability to capture the batch effects as desired (see Table 3), as well as (Table S8 and Fig. S3).

##### AML dataset

**Fig. 4.**
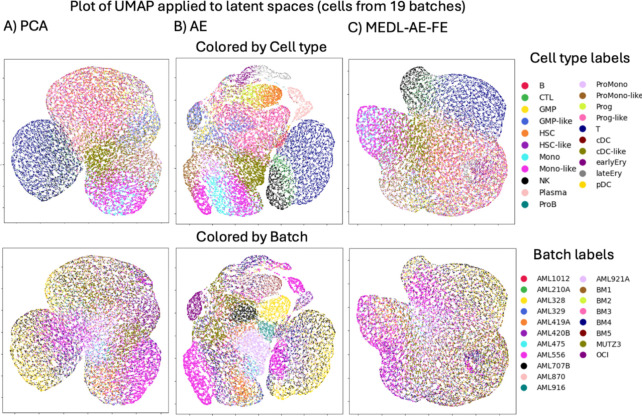
UMAPs from 23,050 cells from the AML dataset latent spaces obtained with A) PCA (Raw), B) AE and C) MEDL-AE-FE.

For the leukemia dataset (AML), regarding batch separability, the PCA model sets the baseline score at −0.28. The AE model fails to suppress batch effects, and has a less negative ASW at −0.19, meanwhile the proposed framework works as intended. The MEDL-AE-FE component in our framework further suppresses batch separability to −0.30 and the MEDL-AE-RE component successfully captures batch variability, with a substantially increased ASW of +0.32 (Table 4, left columns). This dataset exhibits substantial variation in cell type representation across donors, introducing a cell type to donor confounding factor (Fig. S1). Additionally, malignant cell types are exclusively present in donors with the disease and in cell lines. Consequently, regarding cell type separability, even though the MEDL-AE-FE component of our framework is *more* effective at recovering cell type signals compared to PCA, it shows a slight reduction in cell type separability *relative* to the AE (Table 4, right columns, and Fig. 4). This reduction is attributable to the cell type–donor confounding factor; removing batch information leads to the loss of some cell type information (Fig. 4B and 4C).

Upon further analysis, when comparing the latent spaces of the AE and MEDL-AE-FE models, it can be observed that natural killer (NK), cytotoxic T lymphocyte (CTL), and T cells exhibit greater invariance to donor heterogeneity. Notably, these cell types lack malignant counterparts, which explains their increased invariance in the MEDL-AE-FE representation compared to other cell types.

This observation underscores the necessity of a framework like ours with the added MEDL-AE-RE component which can recover donor variability that is removed by the MEDL-AE-FE. As mentioned, the MEDL-AE-RE model effectively captures batch variability, as indicated by all ASW, but also by the 1/DB and CH scores (Table 4 and Table S9). The MEDL-AE-RE latent space further reveals that patients AML328 and AML556, as well as the cell line MUTZ3, exhibit greater heterogeneity compared to other patients and healthy donors (Fig S4). This distinction highlights the framework’s ability to identify and separate highly heterogeneous batches, thereby enhancing our understanding of the underlying variability within the dataset.

In this dataset, the classifier enhances the cell type signal, but the adversarial component diminishes it due to confounding between cell type and batch effects (Table S6). Therefore, we prioritize the model with the strongest cell type signal over maximal batch correction. Increasing the reconstruction loss weight boosts the mean cell type signal, indicating that emphasizing reconstruction loss (and reducing adversarial loss) preserves it. This highlights a trade-off between batch correction and maintaining cell type signal; cell type–donor confounding likely contributes, so optimizing batch correction may obscure cell type distinctions.

#### b) Visualization of learned batch effects

The proposed MEDL batch suppression framework offers novel capabilities for addressing “What if?” scenarios. For instance, how would a cell’s gene expression profile change if the cell came from a different batch? What if the cell originated from a donor with a different diagnosis, or from another donor with the same diagnosis? What if it came from a cell line? This subsection demonstrates the method’s effectiveness in addressing these questions.

##### Healthy Heart Dataset

**Fig. 5.**
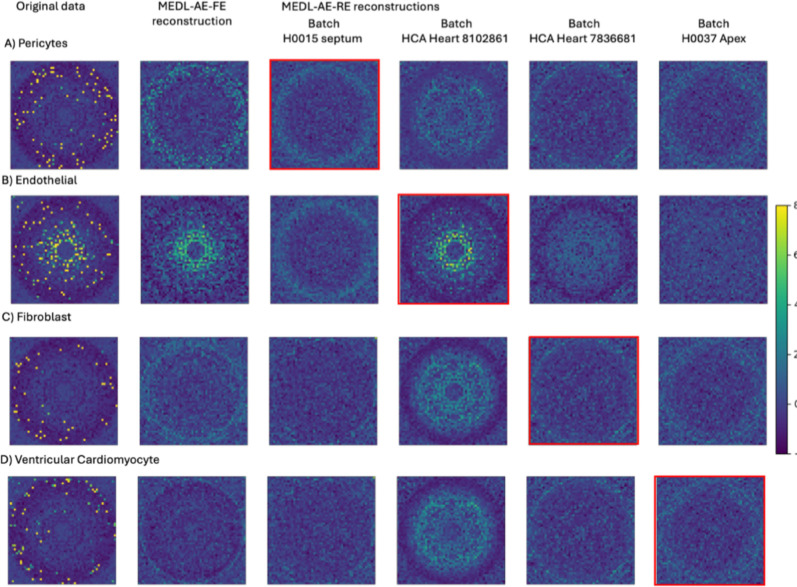
Genomaps show the gene expression of 2916 genes for four cells from different cell types across 4 different batches. The figure columns include the original data, the fixed effects reconstruction from MEDL-AE-FE, along with four counterfactual reconstructions for four different batches using MEDL-AE-RE. The red squares highlight the reconstruction of MEDL-AE-RE for their original batch.

To evaluate the effectiveness of the proposed method in learning a generative model of batch effects, we selected four cell types from the Healthy Heart dataset to represent broad categories: pericytes, endothelial cells, fibroblasts, and ventricular cardiomyocytes. A random cell from each type was chosen, and its gene expression was visualized using a genomap (Fig. 5, first column). A genomap (Islam & Xing, 2023) displays a cell’s gene expression as an image, where each pixel represents a gene, and the pixel’s intensity corresponds to the gene’s scRNA-seq expression. The genes (pixels) are arranged in a polar plot, where genes with equivalent gene-to-gene interactions are positioned at the same distance from the center (origin, radius ρ=0). The angle θ is arbitrary. The first column shows the original gene expression of the selected cells of each cell type, where distinct expression patterns are observed across the cell types. The second column shows the genomap for the fixed effects reconstruction using the MEDL-AE-FE model, which captures the batch-independent characteristics of the cells. That there are distinct gene expression patterns visible, highlights the variability between cell types, independent of batch effects, that are captured by the MEDL-AE-FE model. In the remaining columns, the following question is explored: If the cells had come from a different batch, how would their gene expression pattern differ? To address this, the cells were projected (reconstructed) as if they originated from each of four different batches (shown in columns three through six), including their own batch and three other batches. A red square outline indicates each cell’s original batch. Here we observe that the MEDL-AE-RE model successfully learned batch-to-batch variability, as demonstrated by the visually distinct genomaps across each row for the same cell. Additionally, within each column, the variability between cell types remains apparent in the differing appearances of the genomaps. Taken together, these results demonstrate that the proposed framework captures both cell type-specific and batch-specific patterns across an array of cell types contained within the Healthy Heart dataset.

##### ASD Dataset

**Fig. 6.**
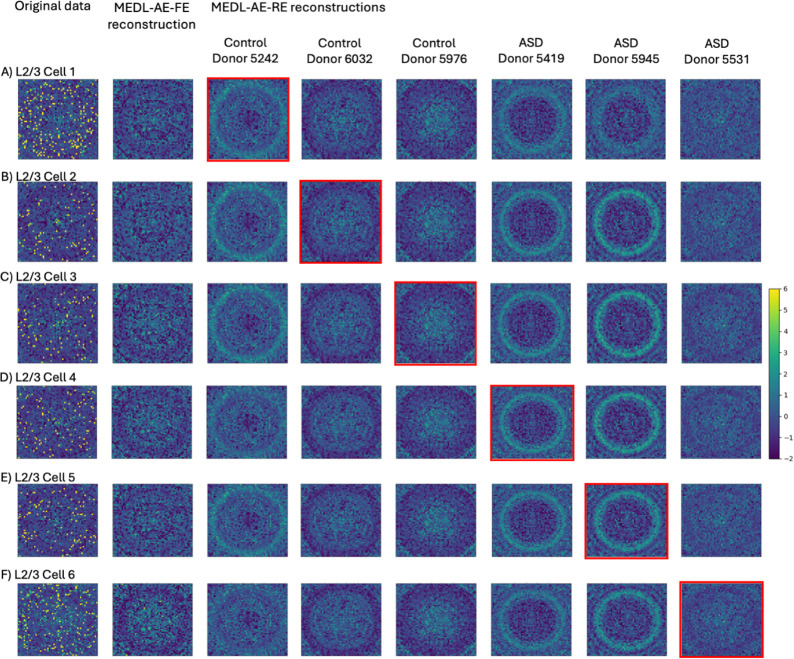
Genomaps show the gene expression of 2916 genes for six L2/3 cells. The figure columns include the original data, the fixed effects reconstruction from MEDL-AE-FE, along with six counterfactual reconstructions for six different donors using MEDL-AE-RE. The red squares highlight the reconstruction of MEDL-AE-RE for their original batch.

In the ASD dataset, the batch effect comes from the variety of donors and the framework learns the donor-specific and donor-agnostic effects. Cells of the L2/3 neuronal subtype were selected, and their gene expression profiles were visualized using genomaps (Fig. 6, first column). The first column reveals distinct genomap patterns between cells from control donors (Fig. 6, rows A-C) and those from autistic donors (Fig. 6, rows D-F), highlighting differences in gene expression associated with autism. The second column displays the fixed effects reconstruction using the MEDL-AE-FE model, capturing cell-type variability independent of donor effects. To address the question of how a cell’s gene expression pattern would appear if it originated from a different donor—specifically, from a subject with autism versus a control—the cells were reconstructed as if they had come from each of several donors, including their own and others (shown in columns three through eight). We observe that the MEDL-AE-RE model effectively captures donor-specific variability, as evidenced by the varying genomap patterns across each row for the same cell when projected onto different donors. Notably, cells projected onto autistic donors exhibit distinct gene expression patterns, such as more pronounced rings, compared to those cells projected onto control donors. The reconstruction corresponding to the cell’s original donor is outlined with the red square. These findings demonstrate that the model captures both cell-type–specific and donor-specific patterns, enabling prediction of how gene expression might differ if a cell had come from a different donor, with the same or different diagnosis.

##### AML Dataset

**Fig. 7.**
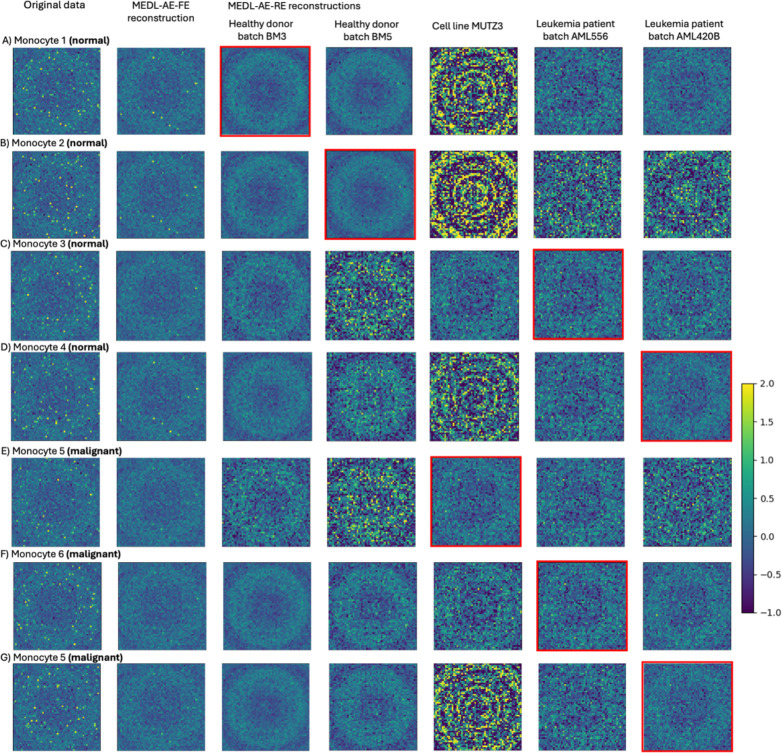
Genomaps show gene expression level of 2916 genes for normal Monocytes (A-D) and malignant Monocytes (E-G) from different batches from 2 healthy donors, 2 leukemia patients (AML) and one cell type (MUTZ3). The figure columns include the original data, the fixed effects reconstruction from MEDL-AE-FE, along with five counterfactual reconstructions for four different batches using MEDL-AE-RE and one cell line. The red squares highlight the reconstruction of MEDL-AE-RE for their original batch.

In the leukemia dataset, the source of the cells (donors and cell lines) is the batch effect. Both normal (Fig. 7, rows A-C) and malignant (Fig. 7, rows D-F) cells are randomly selected for visualization. The first column shows the original gene expression of the selected cells of each cell type, where distinct genomap patterns among the normal and malignant monocytes from different healthy donors and cell lines are observed, highlighting the natural variability between donors capture by the proposed framework. The second column presents the fixed effects reconstruction using the MEDL-AE-FE model, which captures cell variability independent of the donor. In the subsequent columns, the following question is explored: How would a cell’s expression pattern vary if it had come from a different donor—specifically from healthy donors, AML donors, or cell lines? To investigate this, both normal and malignant monocytes were reconstructed as if they originated from various donors and cell lines (shown in columns three through seven). We observe that the MEDL-AE-RE model effectively captures donor-specific and disease-specific variability, as evidenced by the varying genomap patterns across each row for the same cell. Notably, gene expression patterns differ when normal cells are projected onto AML donors versus healthy donors, and vice versa for malignant cells. This demonstrates the model’s ability to predict how gene expression would change if a normal or malignant cell had come from a different donor or cell lines. The reconstruction corresponding to the original donor or cell line of each cell is indicated by the red square. We can also observe that our framework has learned distinct gene expression patterns between healthy donors, AML donors, and cell lines. For instance, the MUTZ3 cell line exhibits high heterogeneity. This underscores the advantage of modeling random effects rather than merely suppressing them, allowing exploration of how gene expression varies between normal and malignant cells as well as across different donors and batches. Overall, the comprehensive generative MEDL-based modeling of both cell-type-specific and donor-specific patterns enhance give a new window through which we can increase our understanding of gene expression variability due to disease status and donor differences.

### 4.2. Experiment 2. Evaluation of how the complementary nature of the FE and RE latent representations enhances prediction performance

The primary goal of experiment 2 is to quantify the impact of embedding a cell-type classifier on batch and cell-type separability. This is achieved by evaluating how the complementary nature of fixed and random effects latent representations can enhance prediction performance across various targets in our databases. Our *first objective* is to determine whether cell type can be predicted more accurately using both latent spaces combined (fixed and random effects) than when using either one alone, and whether this combination yields better cell type classification than using either the PCA or AE latent spaces.

We observed that when a Random Forest classifier is trained to predict a cell’s cell type superior accuracy (ACC = 88.6%, 73.7%, and 60.4%) is achieved across all three datasets (Healthy Heart, ASD, AML) (Table 5: first, second, and fourth rows), when the predictive model uses latent spaces that combine both fixed and random effects of our framework. This performance surpassed that achieved when training on latent spaces derived solely from PCA (ACC = 70.8%, 49.1%, 43.4%) or fixed effects (ACC = 83.5%, 66.9%, 42.7%).

Our second experiment objective is to determine whether diagnosis could be predicted more accurately using both latent spaces. For the ASD dataset, using diagnosis (*typically developing* versus *autism spectrum disorder*) as the target, the Random Forest classifier achieved the highest performance (ACC = 94.6%) when trained on latent spaces fusing (concatenating) both fixed and random effects. In contrast, classifiers trained on latent spaces derived from PCA or fixed effects alone barely exceeded chance accuracy, with ACC of 52.8% and 52.4%, respectively. In the AML dataset, using patient group (healthy donor, leukemia donor, or cell line) as the target variable, the Random Forest classifier trained on latent spaces combining the fixed and random effects attained significantly higher performance (ACC = 94.0%), compared to those trained on latent spaces derived from PCA or fixed effects alone, which attained ACC of 75.5% and 72.0%, respectively. Collectively, the findings of superior accuracy predicting both cell type, diagnosis, and patient group targets, underscore the potential utility of the proposed framework which simultaneously suppresses and quantifies batch effects rather than merely suppressing them, as the information extracted from each part of the framework is complementary.

### 4.3. Experiment 3. Quantification of the impact of an embedded cell type classifier on batch and cell type separability

Since many transcriptomics datasets have an external means to determine cell type, the overall goal of experiment three is to determine the effect of replacing the autoencoder in our framework with an autoencoder classifier of cell type on the subsequent batch and cell type separability.

#### Healthy Heart dataset

**Fig. 8.**
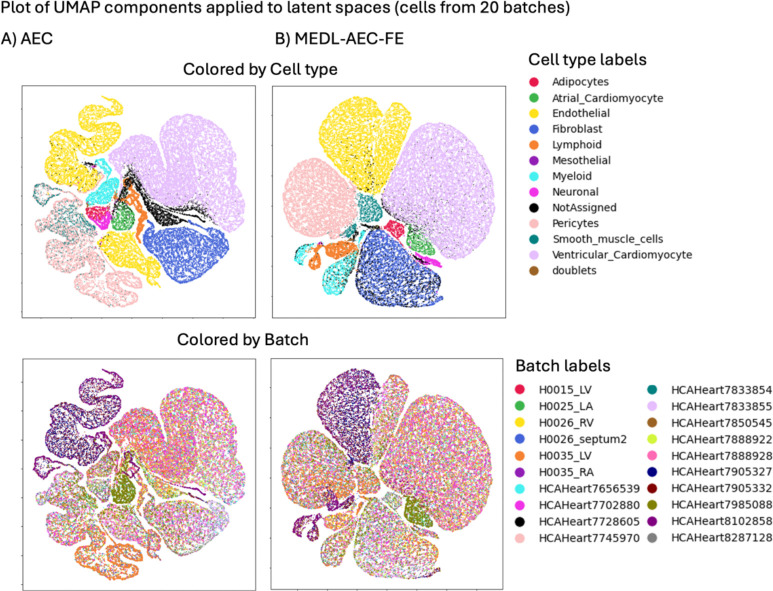
UMAP visualizations of the gene expression latent spaces 44,987 cells from 20 out of 147 batches from the Healthy Heart dataset. The latent spaces were obtained with A) AEC, B) MEDL-AEC-FE.

Integrating a cell type classifier into the base models—resulting in the AEC and MEDL-AEC-FE variants—leads to an improved mean ASW scores for both batch effects and cell type classification within the Healthy Heart dataset (Table 6). Specifically, the mean ASW for cell type classification increased from 0.19 (Table 2) to 0.30 with the AEC (Table 6, right columns), representing substantial a 58% improvement. For the MEDL-AEC-FE model, this score also increased from 0.16 (Table 2) to 0.28 (Table 6), reflecting a similarly impressive 75% improvement. Additionally, although the ASW and CH clustering scores for batch and cell type suggest that the AEC has a slightly better separability scores compared with the MEDL-AEC-FE portion of our proposed framework (Table 6 and S10), the UMAP projections indicate that MEDL-AEC-FE better preserves the biological structure. The MEDL-AEC-FE latent space (Fig. 8B, right column) more effectively maintains cell type biology than both the AEC (Fig. 8A, left column) and MEDL-AE-FE models (Fig. 2C), and it reduces the number of cells within the “Not Assigned” cluster compared to the AEC, which is also desirable as this cell “type” is heterogeneous, and not a singular type. Additionally, including a cell type classifier in the AE model introduced false positives when cell type labels are uncertain. For instance, the AEC (Fig. 8A) displays a significantly larger cluster of “Not Assigned” cells compared to the AE and PCA models (Figs. 2A and 2C).

#### ASD dataset

In the ASD dataset, incorporating a classifier enhanced the MEDL-AE-FE model, increases mean cell type ASW score from 0.20 (Table 3) to 0.27 (Table 7), which represents a sizeable 35% improvement.

**Fig. 9.**
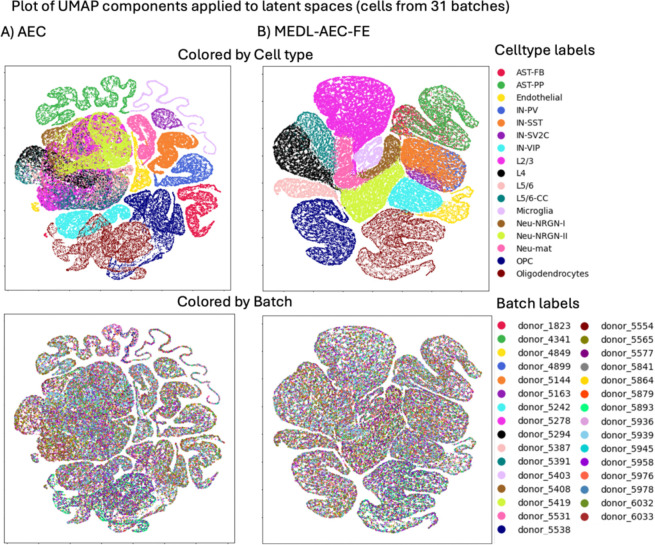
UMAPs from 62735 cells from ASD dataset latent spaces obtained with A) AEC and B) MEDL-AE-CFE.

Although adding the classifier to the AEC model improved the performance of the AE, it did not preserve biological features as effectively as the AE alone, as illustrated in Fig. 3B and Fig 9A. Furthermore, while the AEC model achieves a higher overall score compared to the MEDL-AEC-FE model, the proposed framework more effectively preserves biological characteristics (Fig. 9). Specifically, excitatory neurons (L cells) are more distinctly defined in the MEDL-AEC-FE model, and the boundaries between L cells and NeuN-NRGN (neurogranin-expressing) cells are more pronounced.

#### AML dataset

**Fig. 10.**
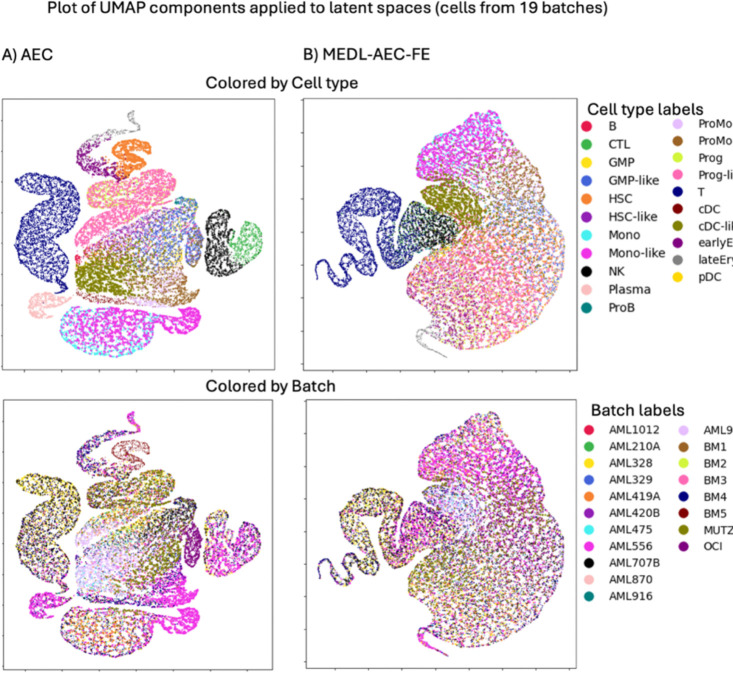
UMAPs from 23,050 cells from the AML dataset latent spaces obtained with A) AEC, B) MEDL-AEC-FE.

On the leukemia dataset, embedding an autoencoder classifier, did not change the ASW scores appreciably. For example, the ASW by cell type remained largely the same in Table 8 versus Table 4. The ASW scores for batch separability stayed the same for the MEDL-AEC-FE at −0.30 while the AEC batch separability improved to about the PCA baseline, though with notably higher variance across cross-validation folds than when using the plain AE. The UMAP projections tell a somewhat different story. Comparing Fig 10 to Figure 4, we observe that there is a noticeable improvement in the cell type separability of both the MEDL-AEC-FE compared to the MEDL-AE-FE and of the AEC compared to the AE, as the cell colors are more noticeably separated in Fig 10 compared to Fig 4. This can be understood, in part because this dataset, contains high-quality cell type labels, enabling an embedded AEC to faithfully preserve additional cell type signal. Within the AEC latent space, in particular, clusters representing monocytes (Mono), progenitor cells (Prog), hematopoietic stem cells (HSC), granulocyte-macrophage progenitors (GMP), and conventional dendritic cells (CDC) from both healthy cells and their malignant counterparts (designated with a “-like” suffix, such as Mono-like) are in proximity and, in some instances, overlap (Fig. 10). This makes sense as they are likely largely similar in their gene expression, differing only in cancer related gene expression. Additionally, the UMAPs (Fig 10, bottom row) also show that the MEDL-AEC-FE method still exhibits a superior ability to suppress batch effects, though as in Experiment 1, this improvement is traded off for cell type separability due to a confounding and overlap between cell type and donor information (Table S6)

## 5. Discussion

We applied our MEDL framework to three datasets where batches contained valuable biological information, highlighting the benefits of modeling batch effects instead of discarding them. In Experiment 1, we found that using only the fixed-effects subnetwork led to a trade-off between suppressing batch effects and preserving cell type signals due to the choice of biologically informative batches. The random-effects subnetwork counterweights this trade-off by capturing the information discarded by the fixed effects, e.g., by modeling batch effects including biological batch effects due to diagnosis or donor. The dual nature to the proposed approach addresses two critical issues: mitigating the loss of cell type information due to batch–cell type confounding and modeling batch effects valuable for describing biological batch heterogeneity.

Unlike other batch correction methods which focus on creating a batch-invariant space (e.g., iMAP (Wang et al., 2021), ResPAN (Wang et al., 2022), scDREAMER (Shree et al., 2023), IMAAE (Wang et al., 2023), ABC (Danino et al., 2024), DB-AAE (Ko & Sartorelli, 2024)), our MEDL framework explicitly learns batch distributions through its random-effects subnetwork. These prior works neither include a random-effects subnetwork nor explicitly model or quantify batch distributions. Our results demonstrate that learning batch distributions is necessary to recover information discarded by the fixed-effects subnetwork, which alone resembles prior work.

By comparing our fixed-effects subnetwork with the autoencoder, our approach enables introspective analysis of the learned latent spaces, identifying cell types exhibiting the most heterogeneity across batches. In the Healthy Heart dataset, the fixed-effects subnetwork alone prevented a misleading clustering of “not assigned” cell types seen in PCA and the traditional autoencoder, thereby avoiding false clustering and cell type identification.

In the second part of Experiment 1, we generated 2D visualizations of cells reconstructed by the MEDL subnetworks, demonstrating the model’s ability to separate batch-invariant and batch-specific gene expression variability at the single-cell level. We utilized the quantified random effects to answer “what if” questions, by reconstructing the changes in a cell’s gene expression profile under the targeted conditions. For example, in the Healthy Heart dataset, we predicted how a cell’s profile would alter if it originated from a different batch, highlighting gene expression differences between cell types. In the ASD and AML datasets, we simulated how cells would appear if they came from donors with different diagnoses or from cell lines, providing insights into disease variability and donor heterogeneity. These visualizations revealed variability across batches and cell types, including: 1) differences between autism and control donors in the ASD dataset, and 2) differences between malignant and normal cells from healthy, AML patients and cell lines in the AML dataset. *Our model’s ability to project cells across different batches represents a significant advancement in personalized single-cell modeling.* Additionally, the AIF model (Monnier & Cournede, 2024) which is one of the few prior works that incorporates batch modeling, still does not explicitly separate batch effects, while our proposed approach offers enhanced explainability by isolating batch variability, facilitating the disentanglement of batch effects from other variations. Overall, our approach is novel in using batch projections to explore and begin to address “what if” questions. We are the first ones to exploit cell projections for hypothetical scenarios or separate fixed and random effects in scRNA-seq data with a deep learning method. This enhances the explainability of our model compared to existing methods.

Traditionally, batch correction evaluation focuses on clustering metrics (Buttner et al., 2019; Haghverdi et al., 2018; Luecken & Theis, 2019; Tran et al., 2020) rather than classification performance. In Experiment 2, a complementary integration of FE and RE latent spaces improved classification accuracy of both cell type and diagnoses. This demonstrates the practical utility of leveraging batch effects information. The combined model outperformed both PCA and the fixed-effects subnetwork alone in classification performance across all tested targets and datasets. This supports the findings of Experiment 1: while the fixed-effects subnetwork discards essential batch-related variability, the random-effects subnetwork captures this variability, leading to more robust and comprehensive modeling of scRNA-seq data.

To preserve the cell type biology within the batch-invariant space, when external cell type information is available, in Experiment 3, we examined the effect of incorporating a cell type classifier into the MEDL-AE-FE subnetwork. Methods like AutoClass (Li et al., 2020), scDREAMER (Shree et al., 2023), and ABC (Danino et al., 2024) incorporate cell type classifiers in their supervised approaches for batch effect suppression and cell type biology preservation. As shown in scDREAMER’s ablation tests, the cell type classifier effectively enhances cell type separability. However, using a cell type classifier requires available cell type labels, which may involve prior annotation prone to human error. Utilizing low-quality or undefined labels can lead to false clustering and incorrect biological interpretations, as we identified in the AEC but mitigated with our MEDL-AEC-FE. This underscores the importance of high-quality labels when adding a cell type classifier for biology preservation. Simple autoencoder models with a cell type classifier like AutoClass may not adequately prevent false positives from mislabeled datasets, potentially leading to erroneous conclusions.

The AML dataset presented unique challenges due to a substantial confound between cell type and batch effect (donor), as certain cell types were present only in specific donors. In such datasets, maintaining the cell type signal is challenging even with a cell type classifier in the fixed-effects subnetwork. This highlights the necessity of effectively modeling batch variability to recover information discarded by batch suppression methods.

In summary, we propose a general framework for batch effect suppression and modeling. The fixed-effects subnetwork we implemented is just one example for that subnetwork. Our framework supports independent training of both fixed (batch effect suppression) and random-effects subnetworks. Therefore the user is free to incorporate a different fixed effects network and hence it allows incorporating other batch-invariant models while pairing with the proposed MEDL-AE-RE model. This could potentially further enhance overall performance, and an avenue to explore in future work.

## 6. Limitations

Our scRNA-seq MEDL subnetworks used a latent space of dimensions (n,2) with n cells and a bottleneck dimension of 2. We leave for future work to assess performance in higher-dimensional latent spaces.

Showing how our random-effects subnetwork complements other batch suppression methods remains for future work. This study explores a mixed-effects deep learning framework to both suppress and model batch effects, which has the potential to complement existing methods focused solely on suppression. Our flexible framework reveals new capabilities for addressing biological questions—a capacity traditional batch suppression methods lack.

The framework requires hyperparameter optimization, particularly the weights assigned to loss functions in the fixed and random-effects subnetworks. Factors like the number of batches and cell types must be considered to adjust these weights, ensuring all loss functions are on a similar scale. For instance, with more classes, each class’s contribution to the total loss diminishes, which can be addressed by tuning the weights of each individual loss function.

Finally, although we tested on diverse datasets covering various diseases and body regions, our evaluation is not exhaustive. A comprehensive assessment is beyond the scope of this initial demonstration.

## 7. Conclusions

In this study, we introduced a mixed effects deep learning (MEDL) method for single-cell RNA sequencing (scRNA-seq) data analysis, offering a novel approach that quantitatively and separately models fixed (batch invariant) and random (batch specific) effects. Our key contributions are: 1) the development of a novel MEDL framework that, to our knowledge, is the first in scRNA-seq analysis to separately model batch-invariant biological signals and batch-specific variability, 2) demonstrating that this framework effectively suppresses batch effects through the fixed effects subnetwork, creating a batch-invariant latent space, while the random effects subnetwork captures batch-specific variability, thereby enhancing accuracy and interpretability, 3) exploring ‘what-if’ analyses by leveraging the modeled batch effects—such as predicting a cell’s expression profile if it originated from a different batch or from a subject with a different diseased diagnosis—exemplified through genomap visualizations revealing subtypes in the ASD dataset and donor and cell line variability in the AML dataset, 4) achieving significant improvements in classification accuracy by combining the fixed (batch-agnostic) and random (batch-representative) effects representations, suggesting that retaining batch variability alongside batch invariance enhances performance and a unique window for insights into biological data, 5) preventing false clustering observed in PCA, autoencoders, and autoencoders with a cell type classifier when using low-quality labels, 6) preserving cell type biology in the latent space through the addition of a cell type classifier to the fixed effects subnetwork. These six key contributions were demonstrated across diverse scRNA-seq datasets encompassing various health conditions, cell types, and batch effects, underscoring our methods potential and the necessity of both fixed and random effects latent spaces for comprehensive data modeling.

## Supplementary Material

Supplement 1

## Figures and Tables

**Table 1.** Details of Preprocessed datasets used to evaluate MEDL models.

	Healthy Heart (Litvinukova et al., 2020; Yu et al., 2023b)	ASD and Controls (Velmeshev et al., 2019)	AML and healthy (van Galen et al., 2019)

Description	Heart Cells of Different Tissues	ASD and Controls	Samples of Healthy and AML Subjects
Data Type	Single cell	Single nuclei	Single cell
Total Cells (Pre-Filtering)	486,134	104, 559	41,090
Total Cells (Post-Filtering)	486,134	104, 559	38,417
Total Genes	33,538	36,501	27,899
High Variable Genes	3,000	2,916	2,916
Number of Cell Types	12 + Not assigned cell type category	17	6 Malignant + 15 Healthy
Batch Effect	Batch (z =147)	Donor (z = 31)	Donor (z=19)

**Table 2.** Average Silhouette Width (ASW) scores (Mean and 95% CI across 5 folds) for batch and cell type separability in the latent spaces of the Healthy Heart dataset, using PCA, AE, MEDL-AE-FE, and MEDL-AE-RE models.

	ASW (batch)			ASW (cell type)		
	mean	95% CI		mean	95% CI	

PCA (Baseline)	−0.48	−0.51	−0.46	−0.05	−0.06	−0.05
AE	−0.45	−0.48	−0.43	**0.19**	**0.15**	**0.23**
MEDL-AE-FE	**−0.50**	**−0.52**	**−0.48**	**0.16**	**0.09**	**0.23**
MEDL-AE-RE	**0.37**	**0.16**	**0.58**	−0.28	−0.45	−0.11

**Table 3:** Average Silhouette Width (ASW) scores (Mean and 95% CI across 5 folds) for batch and cell type separability in the latent spaces of the ASD dataset, using PCA, AE, MEDL-AEFE, and MEDL-AE-RE models.

	ASW (batch)			ASW (cell type)		
	mean	95% CI		mean	95% CI	

PCA (Baseline)	−0.31	−0.33	−0.30	0.07	0.06	0.08
AE	−0.22	−0.23	−0.20	**0.33**	**0.31**	**0.36**
MEDL-AE-FE	−0.23	−0.26	−0.21	**0.20**	**0.16**	**0.23**
MEDL-AE-RE	**−0.11**	**−0.49**	**0.27**	−0.19	−0.28	−0.10

**Table 4.** Average Silhouette Width (ASW) scores (Mean and 95% CI across 5 folds) for batch and cell type separability in the latent spaces of the AML dataset, using PCA, AE, MEDL-AE-FE, and MEDL-AE-RE models.

	ASW (batch)			ASW (cell type)		
	mean	95% CI		mean	95% CI	
PCA (Baseline)	−0.28	−0.29	−0.28	−0.09	−0.09	−0.08
AE	−0.19	−0.21	−0.17	**0.03**	**0.02**	**0.05**
MEDL-AE-FE	**−0.30**	**−0.31**	**−0.28**	−0.05	−0.07	−0.04
MEDL-AE-RE	**0.32**	**0.23**	**0.42**	−0.36	−0.45	−0.28

**Table 5.** Mean results across 5 folds and 95% confidence intervals (CI) obtained from the Random Forest Classifier applied to the Healthy Heart, ASD, and AML datasets, with cell type as the primary target. In addition, the ASD dataset includes diagnosis as a secondary target, and the AML Dataset includes patient group as a secondary target. The performance metrics reported include Accuracy, Balanced Accuracy, and Chance Accuracy.

			Accuracy			Balanced Accuracy			Chance Accuracy		
Dataset	Target	Latent space	mean	95%CI		mean	95%CI		mean	95%CI	

Healthy Heart (n = 486,134)	Cell types (k = 13)	PCA	70.8%	70.6%	70.9%	37.2%	37.0%	37.5%	16.2%	16.1%	16.3%
		MEDL-AE-FE	83.5%	82.2%	84.9%	57.4%	54.2%	60.6%			
		MEDL-AE (FE + RE)	**88.6%**	**88.2%**	**88.9%**	**67.3%**	**65.1%**	**69.4%**			



ASD (n = 104,559)	Cell types (k = 17)	PCA	49.1%	48.6%	49.5%	38.1%	37.7%	38.6%	7.9%	7.8%	8.0%
		MEDL-AE-FE	66.9%	61.5%	72.2%	59.1%	52.0%	66.1%			
		MEDL-AE (FE + RE)	**73.7%**	**69.8%**	**77.6%**	**67.0%**	**61.6%**	**72.4%**			



	Diagnosis (k = 2)	PCA	52.8%	52.5%	53.2%	52.8%	52.5%	53.2%	50.1%	49.7%	50.4%
		MEDL-AE-FE	52.4%	52.0%	52.7%	52.4%	52.0%	52.7%			
		MEDL-AE (FE + RE)	**94.6%**	**89.6%**	**99.6%**	**94.6%**	**89.6%**	**99.6%**			



AML (n = 38,417)	Cell types (k = 21)	PCA	43.4%	42.6%	44.2%	30.3%	29.5%	31.0%	8.0%	7.9%	8.1%
		MEDL-AE-FE	42.7%	40.7%	44.7%	30.0%	28.5%	31.6%			
		MEDL-AE (FE + RE)	**60.4%**	**58.0%**	**62.8%**	**47.8%**	**45.1%**	**50.5%**			



	Patient group (k = 3)	PCA	75.5%	75.1%	75.9%	51.2%	50.2%	52.3%	60.8%	60.4%	61.3 %
		MEDL-AE-FE	72.0%	71.7%	72.4%	40.0%	38.6%	41.4%			
		MEDL-AE (FE + RE)	**94.0%**	**87.9%**	**100.0%**	**88.1%**	**76.3%**	**100.0%**			

**Table 6.** Average Silhouette Width (ASW) scores (Mean and 95% CI across 5 folds) for batch and cell type separability in the latent spaces of the Healthy Heart dataset, using AEC and MEDL-AEC-FE.

	ASW (batch)			ASW (cell type)		
	mean	95% CI		mean	95% CI	

AEC	−0.59	−0.65	−0.54	**0.30**	0.25	0.36
MEDL-AEC-FE	−0.55	−0.57	−0.52	0.28	0.18	0.39

**Table 7.** Average Silhouette Width (ASW) scores (Mean and 95% CI across 5 folds) for batch and cell type separability in the latent spaces of the ASD dataset, using AEC and MEDL-AEC-FE.

	ASW (batch)			ASW (cell type)		
	mean	95% CI		mean	95% CI	

AEC	−0.26	−0.30	−0.22	**0.36**	0.29	0.43
MEDL-AEC-FE	−0.22	−0.26	−0.19	0.27	0.21	0.33

**Table 8.** Average Silhouette Width (ASW) scores (Mean and 95% CI across 5 folds) for batch and cell type separability in the latent spaces of the AML dataset, using AEC and MEDL-AEC-FE.

	ASW (batch)			ASW (cell type)		
	mean	95% CI		mean	95% CI	

AEC	−0.29	−0.34	−0.24	**0.03**	−0.04	0.10
MEDL-AEC-FE	−0.30	−0.37	−0.24	−0.05	−0.08	−0.02

## Data Availability

Upon acceptance of this publication in a journal, all code for this paper including interpretable scRNA-seq analysis, ablation tests and reproducibility scripts, will be released on GitHub: https://github.com/DeepLearningForPrecisionHealthLab/MEDLforRNAseqData. Gene expression matrices and cell annotations for the AML dataset were obtained from the Gene Expression Omnibus (GEO) under accession number GSE116256 (van Galen et al., 2019). The Healthy Heart dataset was acquired from Yu et al. (2023) via figshare (DOI: https://doi.org/10.6084/m9.figshare.20499630.v2) (Yu et al., 2023b), though they also reference the Heart Cell Atlas (https://www.heartcellatlas.org/) (Litvinukova et al., 2020). The log2-transformed ASD dataset was downloaded from the UCSC Cell Browser (https://autism.cells.ucsc.edu)(Speir et al., 2021; Velmeshev et al., 2019)

## References

[R1] Blei D. M., Kucukelbir A., & McAuliffe J. D. (2017). Variational Inference: A Review for Statisticians. Journal of the American Statistical Association, 112(518), 859–877. 10.1080/01621459.2017.1285773

[R2] Bouland G. A., Mahfouz A., & Reinders M. J. T. (2023). Consequences and opportunities arising due to sparser single-cell RNA-seq datasets. Genome Biol, 24(1), 86. 10.1186/s13059-023-02933-w 37085823 PMC10120229

[R3] Breiman L. (2001). Machine Learning, 45(1), 5–32. 10.1023/a:1010933404324

[R4] Buttner M., Miao Z., Wolf F. A., Teichmann S. A., & Theis F. J. (2019). A test metric for assessing single-cell RNA-seq batch correction. Nat Methods, 16(1), 43–49. 10.1038/s41592-018-0254-1 30573817

[R5] Calinski T., & Harabasz J. (1974). A dendrite method for cluster analysis. Communications in Statistics - Theory and Methods, 3(1), 1–27. 10.1080/03610927408827101

[R6] Dai C., Chen M., Wang C., & Hao X. (2021). Deconvolution of Bulk Gene Expression Profiles with Single-Cell Transcriptomics to Develop a Cell Type Composition-Based Prognostic Model for Acute Myeloid Leukemia. Front Cell Dev Biol, 9, 762260. 10.3389/fcell.2021.762260 34869351 PMC8633313

[R7] Danino R., Nachman I., & Sharan R. (2024). Batch correction of single-cell sequencing data via an autoencoder architecture. Bioinform Adv, 4(1), vbad186. 10.1093/bioadv/vbad186 38213820 PMC10781938

[R8] Davies D. L., & Bouldin D. W. (1979). A Cluster Separation Measure. IEEE Transactions on Pattern Analysis and Machine Intelligence, PAMI-1(2), 224–227. 10.1109/tpami.1979.4766909 21868852

[R9] Gronbech C. H., Vording M. F., Timshel P. N., Sonderby C. K., Pers T. H., & Winther O. (2020). scVAE: variational auto-encoders for single-cell gene expression data. Bioinformatics, 36(16), 4415–4422. 10.1093/bioinformatics/btaa293 32415966

[R10] Haghverdi L., Lun A. T. L., Morgan M. D., & Marioni J. C. (2018). Batch effects in single-cell RNA-sequencing data are corrected by matching mutual nearest neighbors. Nat Biotechnol, 36(5), 421–427. 10.1038/nbt.4091 29608177 PMC6152897

[R11] Hu Y., Xie M., Li Y., Rao M., Shen W., Luo C., Qin H., Baek J., & Zhou X. M. (2024). Benchmarking clustering, alignment, and integration methods for spatial transcriptomics. Genome Biol, 25(1), 212. 10.1186/s13059-024-03361-0 39123269 PMC11312151

[R12] Hwang B., Lee J. H., & Bang D. (2018). Single-cell RNA sequencing technologies and bioinformatics pipelines. Exp Mol Med, 50(8), 1–14. 10.1038/s12276-018-0071-8 PMC6082860 30089861

[R13] Hwang H., Jeon H., Yeo N., & Baek D. (2024). Big data and deep learning for RNA biology. Exp Mol Med, 56(6), 1293–1321. 10.1038/s12276-024-01243-w 38871816 PMC11263376

[R14] Islam M. T., & Xing L. (2023). Cartography of Genomic Interactions Enables Deep Analysis of Single-Cell Expression Data. Nat Commun, 14(1), 679. 10.1038/s41467-023-36383-6 36755047 PMC9908983

[R15] Ko K. D., & Sartorelli V. (2024). A deep learning adversarial autoencoder with dynamic batching displays high performance in denoising and ordering scRNA-seq data. iScience, 27(3), 109027. 10.1016/j.isci.2024.109027 38361616 PMC10867661

[R16] Li H., Brouwer C. R., & Luo W. (2022). A universal deep neural network for in-depth cleaning of single-cell RNA-Seq data. Nat Commun, 13(1), 1901. 10.1038/s41467-022-29576-y 35393428 PMC8990021

[R17] Li X., Wang K., Lyu Y., Pan H., Zhang J., Stambolian D., Susztak K., Reilly M. P., Hu G., & Li M. (2020). Deep learning enables accurate clustering with batch effect removal in single-cell RNA-seq analysis. Nat Commun, 11(1), 2338. 10.1038/s41467-020-15851-3 32393754 PMC7214470

[R18] Li X., Zhang S., & Wong K. C. (2021). Deep embedded clustering with multiple objectives on scRNA-seq data. Brief Bioinform, 22(5). 10.1093/bib/bbab090 33822877

[R19] Litvinukova M., Talavera-Lopez C., Maatz H., Reichart D., Worth C. L., Lindberg E. L., Kanda M., Polanski K., Heinig M., Lee M., Nadelmann E. R., Roberts K., Tuck L., Fasouli E. S., DeLaughter D. M., McDonough B., Wakimoto H., Gorham J. M., Samari S.,…Teichmann S. A. (2020). Cells of the adult human heart. Nature, 588(7838), 466–472. 10.1038/s41586-020-2797-4 32971526 PMC7681775

[R20] Luecken M. D., Buttner M., Chaichoompu K., Danese A., Interlandi M., Mueller M. F., Strobl D. C., Zappia L., Dugas M., Colome-Tatche M., & Theis F. J. (2022). Benchmarking atlas-level data integration in single-cell genomics. Nat Methods, 19(1), 41–50. 10.1038/s41592-021-01336-8 34949812 PMC8748196

[R21] Luecken M. D., & Theis F. J. (2019). Current best practices in single-cell RNA-seq analysis: a tutorial. Mol Syst Biol, 15(6), e8746. 10.15252/msb.20188746 31217225 PMC6582955

[R22] Martín Abadi A. A., Barham Paul, Brevdo Eugene,, Zhifeng Chen C. C., Corrado Greg S., Davis Andy,, Jeffrey Dean M. D., Ghemawat Sanjay, Goodfellow Ian,, Andrew Harp G. I., Isard Michael, Jozefowicz Rafal, Jia Yangqing,, Lukasz Kaiser M. K., Levenberg Josh, Mané Dan, Schuster Mike,, Rajat Monga S. M., Murray Derek, Olah Chris, Shlens Jonathon,, t Steiner Benoi I. S., Talwar Kunal, Tucker Paul,, Vincent Vanhoucke V. V., Viégas Fernanda,, Oriol Vinyals P. W., Wattenberg Martin, Wicke Martin,, & Yuan Yu a. X. Z. (2015). TensorFlow: Large-scale machine learning on heterogeneous systems. White Paper. 10.5281/zenodo.4724125

[R23] McInnes L., Healy J., & Melville J. (2018). Umap: uniform manifold approximation and projection for dimension reduction. arXiv. 10.48550/ARXIV.1802.03426

[R24] Monnier L., & Cournede P. H. (2024). A novel batch-effect correction method for scRNA-seq data based on Adversarial Information Factorization. PLoS Comput Biol, 20(2), e1011880. 10.1371/journal.pcbi.1011880 38386700 PMC10914288

[R25] Nguyen K. P., Treacher A. H., & Montillo A. A. (2023). Adversarially-Regularized Mixed Effects Deep Learning (ARMED) Models Improve Interpretability, Performance, and Generalization on Clustered (non-iid) Data. IEEE Trans Pattern Anal Mach Intell, 45(7), 8081–8093. 10.1109/TPAMI.2023.3234291 37018678 PMC10644386

[R26] Ning Z., Dai Z., Zhang H., Chen Y., & Yuan Z. (2023). A clustering method for small scRNA-seq data based on subspace and weighted distance. PeerJ, 11, e14706. 10.7717/peerj.14706 36710872 PMC9879162

[R27] Pedregosa F., Eickenberg M., Ciuciu P., Thirion B., & Gramfort A. (2015). Data-driven HRF estimation for encoding and decoding models. Neuroimage, 104, 209–220. 10.1016/j.neuroimage.2014.09.060 25304775

[R28] Rousseeuw P. J. (1987). Silhouettes: A graphical aid to the interpretation and validation of cluster analysis. Journal of Computational and Applied Mathematics, 20, 53–65. 10.1016/0377-0427(87)90125-7

[R29] Shree A., Pavan M. K., & Zafar H. (2023). scDREAMER for atlas-level integration of single-cell datasets using deep generative model paired with adversarial classifier. Nat Commun, 14(1), 7781. 10.1038/s41467-023-43590-8 38012145 PMC10682386

[R30] Speir M. L., Bhaduri A., Markov N. S., Moreno P., Nowakowski T. J., Papatheodorou I., Pollen A. A., Raney B. J., Seninge L., Kent W. J., & Haeussler M. (2021). UCSC Cell Browser: visualize your single-cell data. Bioinformatics, 37(23), 4578–4580. 10.1093/bioinformatics/btab503 34244710 PMC8652023

[R31] Tran H. T. N., Ang K. S., Chevrier M., Zhang X., Lee N. Y. S., Goh M., & Chen J. (2020). A benchmark of batch-effect correction methods for single-cell RNA sequencing data. Genome Biol, 21(1), 12. 10.1186/s13059-019-1850-9 31948481 PMC6964114

[R32] Tung P. Y., Blischak J. D., Hsiao C. J., Knowles D. A., Burnett J. E., Pritchard J. K., & Gilad Y. (2017). Batch effects and the effective design of single-cell gene expression studies. Sci Rep, 7, 39921. 10.1038/srep39921 28045081 PMC5206706

[R33] van Galen P., Hovestadt V., Wadsworth Ii M. H., Hughes T. K., Griffin G. K., Battaglia S., Verga J. A., Stephansky J., Pastika T. J., Lombardi Story J., Pinkus G. S., Pozdnyakova O., Galinsky I., Stone R. M., Graubert T. A., Shalek A. K., Aster J. C., Lane A. A., & Bernstein B. E. (2019). Single-Cell RNA-Seq Reveals AML Hierarchies Relevant to Disease Progression and Immunity. Cell, 176(6), 1265–1281 e1224. 10.1016/j.cell.2019.01.031 30827681 PMC6515904

[R34] Velmeshev D., Schirmer L., Jung D., Haeussler M., Perez Y., Mayer S., Bhaduri A., Goyal N., Rowitch D. H., & Kriegstein A. R. (2019). Single-cell genomics identifies cell type-specific molecular changes in autism. Science, 364(6441), 685–689. 10.1126/science.aav8130 31097668 PMC7678724

[R35] Vendramin L., Campello R. J. G. B., & Hruschka E. R. (2010). Relative clustering validity criteria: A comparative overview. Statistical Analysis and Data Mining: The ASA Data Science Journal, 3(4), 209–235. 10.1002/sam.10080

[R36] Vestal B. E., Wynn E., & Moore C. M. (2022). lmerSeq: an R package for analyzing transformed RNA-Seq data with linear mixed effects models. BMC Bioinformatics, 23(1), 489. 10.1186/s12859022-05019-9 36384492 PMC9670578

[R37] Wang D., Hou S., Zhang L., Wang X., Liu B., & Zhang Z. (2021). iMAP: integration of multiple single-cell datasets by adversarial paired transfer networks. Genome Biol, 22(1), 63. 10.1186/s13059-021-02280-8 33602306 PMC7891139

[R38] Wang L., Hong C., Song J., & Yao J. (2024). CTEC: a cross-tabulation ensemble clustering approach for single-cell RNA sequencing data analysis. Bioinformatics, 40(4). 10.1093/bioinformatics/btae130 PMC10985676 38552307

[R39] Wang T., Johnson T. S., Shao W., Lu Z., Helm B. R., Zhang J., & Huang K. (2019). BERMUDA: a novel deep transfer learning method for single-cell RNA sequencing batch correction reveals hidden high-resolution cellular subtypes. Genome Biol, 20(1), 165. 10.1186/s13059-019-1764-6 31405383 PMC6691531

[R40] Wang X., Zhang C., Wang L., & Zheng P. (2023). Integrating Multiple Single-Cell RNA Sequencing Datasets Using Adversarial Autoencoders. Int J Mol Sci, 24(6). 10.3390/ijms24065502 PMC10056671 36982574

[R41] Wang Y., Liu T., & Zhao H. (2022). ResPAN: a powerful batch correction model for scRNA-seq data through residual adversarial networks. Bioinformatics, 38(16), 3942–3949. 10.1093/bioinformatics/btac427 35771600 PMC9364370

[R42] Wolf F. A., Angerer P., & Theis F. J. (2018). SCANPY: large-scale single-cell gene expression data analysis. Genome Biol, 19(1), 15. 10.1186/s13059-017-1382-0 29409532 PMC5802054

[R43] Wu Y., & Zhang K. (2020). Tools for the analysis of high-dimensional single-cell RNA sequencing data. Nat Rev Nephrol, 16(7), 408–421. 10.1038/s41581-020-0262-0 32221477

[R44] Yousuff M., Babu R., & Rathinam A. (2024). Nonlinear dimensionality reduction based visualization of single-cell RNA sequencing data. Journal of Analytical Science and Technology, 15(1). 10.1186/s40543-023-00414-0

[R45] Yu L., Cao Y., Yang J. Y. H., & Yang P. (2022). Benchmarking clustering algorithms on estimating the number of cell types from single-cell RNA-sequencing data. Genome Biol, 23(1), 49. 10.1186/s13059-022-02622-0 35135612 PMC8822786

[R46] Yu X., Xu X., Zhang J., & Li X. (2023a). Batch alignment of single-cell transcriptomics data using deep metric learning. Nat Commun, 14(1), 960. 10.1038/s41467-023-36635-5 36810607 PMC9944958

[R47] Yu X., Xu X., Zhang J., & Li X. (2023b). Batch alignment of single-cell transcriptomics data using deep metric learning. figshare. 10.6084/m9.figshare.20499630.v2 PMC9944958 36810607

