# scMEDAL for the interpretable analysis of single-cell transcriptomics data with batch effect visualization using a deep mixed effects autoencoder

**Published:** 2025-03-13

**Authors:** Aixa X. Andrade, Son Nguyen, Albert Montillo

## Abstract

scRNA-seq data has the potential to provide new insights into cellular heterogeneity and data acquisition; however, a major challenge is unraveling confounding from technical and biological batch effects. Existing batch correction algorithms suppress and discard these effects, rather than quantifying and modeling them. Here, we present scMEDAL, a framework for single-cell Mixed Effects Deep Autoencoder Learning, which separately models batch-invariant and batch-specific effects using two complementary autoencoder networks. One network is trained through adversarial learning to capture a batch-invariant representation, while a Bayesian autoencoder learns a batch-specific representation. Comprehensive evaluations spanning conditions (e.g., autism, leukemia, and cardiovascular), cell types, and technical and biological effects demonstrate that scMEDAL suppresses batch effects while modeling batch-specific variation, enhancing accuracy and interpretability. Unlike prior approaches, the framework's fixed- and random-effects autoencoders enable retrospective analyses, including predicting a cell's expression as if it had been acquired in a different batch via genomap projections at the cellular level, revealing the impact of biological (e.g., diagnosis) and technical (e.g., acquisition) effects. By combining scMEDAL's batch-agnostic and batch-specific latent spaces, it enables more accurate predictions of disease status, donor group, and cell type, making scMEDAL a valuable framework for gaining deeper insight into data acquisition and cellular heterogeneity.

### 1. Introduction

Data from single-cell RNA sequencing has the potential to provide key new insights into cellular heterogeneity by capturing gene expression variations at single-cell resolution. However, challenges inherent to the data including data sparsity[1], high dimensionality[2], and batch effects[3], complicate its analysis. Batch effects, which can arise from biological variability (e.g., differences in donors or patients sampled, cell cycle stage, or tissue collection) or technical factors[4,5], (e.g., library preparation, and sequencing platform) hinder the identification of biological relationships such as cell types and gene expression patterns. Meanwhile, the continual reduction in the cost of scRNA-seq acquisition has led to the growing availability of such data[6] and an increased interest in the development of batch correction methods to extract as much value as possible from the data. Autoencoder-based deep learning methods have been proposed due to their ability to handle non-linearity, flexibility, and scalability by transforming data into a batch-invariant space. These methods aim to learn a reduced dimensional representation through a form of self-supervised learning, and notable examples include BERMUDA[7], DESC[8], scVAE[9], and AutoClass[10].

Generative adversarial networks (GANs) have also been employed for batch correction, where multiple neural networks compete to explain the data. A generator network learns to produce batch-invariant data, and a discriminator distinguishes between real and fake data. The combination of GANs with autoencoders and mutual nearest-neighbor (MNN) pairing has been proposed. For instance, the iMAP approach uses an adversarial network with MNN pairing to align datasets to an anchor batch[11], while the ResPAN approach introduces a residual autoencoder with skip connections, utilizing MNN without relying on an anchor[12]. Recently proposed methods further build upon the adversarial framework by incorporating batch and cell-type classifiers into adversarial autoencoders. For example, scDREAMER

integrates a batch classifier and, optionally, a cell-type classifier for supervised batch correction[13]. Similarly, IMAAE employs an anchor batch GAN approach combined with a cell-type classifier[14]. The Autoencoder-based Batch Correction (ABC) method introduces two cell-type classifiers applied to the latent space and reconstructed data[15]. Meanwhile, the Dynamic Batching Adversarial Autoencoder (DB-AAE) presents a novel strategy by varying batch sizes during training to reduce noise in scRNA-seq data[16].

However, the aforementioned batch correction methods focus on removing batch effects without explicitly modeling them, potentially leading to overcorrection or loss of biological information—especially when signals are intertwined with batch effects[17]. Even though methods incorporate cell-type labels (e.g., ABC, AutoClass) or impose similarity constraints (e.g., ResPAN, Adversarial Information Factorization (AIF)) to enhance cell-type signals, balancing effective batch correction with the retention of biological variation remains challenging. We hypothesize that characterizing batch variability will provide multiple benefits, including an improved understanding of scRNA-seq data and enabling accurate predictions of biological states at the cellular level, including diagnoses and cell types. Therefore, this work develops a novel batch suppression framework, scMEDAL, which is the first to separately characterize and model batch-invariant and batch-specific variations using a mixed effects deep learning approach. This framework, called scMEDAL (***s**ingle-**c**ell **M**ixed **E**ffects **D**eep **A**utoencoder **L**earning*), is based upon the mathematical foundation of the statistical mixed effects model and is implemented with two complementary and parallel deep autoencoder neural networks. One network is based on adversarial deep learning and learns the batch-invariant scRNA-seq representation (i.e., the fixed effects), while the other is a *Bayesian* neural network autoencoder which learns the batch-specific scRNA-seq probabilistic representation (i.e., the random effects) through variational inference. Unlike other batch correction methods (e.g., iMAP[11], ResPAN[12], scDREAMER[13], IMAAE[14], ABC[15], DB-AAE[16]) that focus on creating batch-invariant spaces without including a random-effects subnetwork or explicitly modeling batch distributions, scMEDAL explicitly learns batch distributions, which we show are necessary for recovering meaningful signals that might otherwise be discarded by fixed-effects only approaches.

The scMEDAL framework is comprehensively evaluated across diverse single-cell and single-nucleus RNA-seq datasets spanning multiple health conditions, cell types, and batch effects. These datasets include data from cardiovascular health (Healthy Heart), Autism Spectrum Disorder (ASD), and Acute Myeloid Leukemia (AML), spanning over 50 different cell types, and incorporating technical batch effects (e.g., sequencer and batch preparation) and biological effects (e.g., disease status and donor group). In all cases, scMEDAL demonstrates effective batch effect suppression while capturing batch-specific variability, enhancing accuracy and interpretability. By leveraging the generative nature of the framework's fixed- and random-effects autoencoder networks, scMEDAL can answer retrospective, "What if?" analyses including predicting a cell's expression under different batches through genomap projections at the cellular level. This reveals the impact of biological batch effects (e.g., the diagnosis) on cellular heterogeneity and the impact of technical batch effects on the gene expression profile, as well as the potential impact of further steps taken to control such variability. Additionally, by combining scMEDAL's complementary batch-agnostic and batch-specific latent spaces, more accurate predictions of disease status, donor group, and cell type are enabled than when using any singular latent space. Furthermore, when cell-type information is available, we demonstrate that the scMEDAL framework can be seamlessly extended to incorporate this information through a cell-type classifier in the fixed-effects subnetwork to further improve cell-type preservation, as shown in Uniform Manifold Approximation and Projection (UMAP)[18] visualizations.

## 2. Results

### 2.1 Overview of the scMEDAL framework: Capturing fixed and random effects in scRNA-seq data

The architecture of the scMEDAL framework, illustrated in **Fig**. 1, is designed for single-cell RNA sequencing (scRNA-seq) data. scMEDAL addresses batch effects—technical or biological variations that can confound analyses—by separately modeling and quantifying the fixed and random effects across the gene expression vectors of the cells. Fixed effects represent batch-invariant features, while random effects capture batch- or donor-specific variations. By separately modeling both types of effects, scMEDAL decouples batch effects from the data, enhancing explainability and preserving biologically meaningful information. The scMEDAL framework processes a gene expression count matrix $\boldsymbol{X} \in \mathbb{R}^{n \times g}$, where $n$ is the number of cells and $g$ is the number of genes. The framework learns two lower dimensional latent space representations: one encodes batch-agnostic fixed effects, referred to as the FE latent space representation, and the other captures batch-specific random effects, called the RE latent space representation. The architecture of our framework includes two parallel subnetworks, each of which is a type of autoencoder. (1) The *Fixed Effects subnetwork* (***scMEDAL-FE***) suppresses batch effects by learning a batch-invariant latent representation of the gene expression data. Its encoder and decoder each have two dense hidden layers with weight tying and are guided by the addition of an adversarial classifier that aims to predict the batch label, $\boldsymbol{z}$ (**Fig.** 1) using the intermediate outputs from the encoder layers. To the extent that it can, this classifier penalizes the encoder, thereby encouraging the learning of an embedding that is not predictive of the batch but is still informative for gene expression reconstruction. (2) The *Random Effects subnetwork* (***scMEDAL-RE***) is a *Bayesian* autoencoder, which learns a batch-specific representation. A Bayesian network replaces the traditional point estimate weights with weight distributions to model the RE as random variables. It is efficiently trained via variational inference. This generative model can be used to understand how gene expression profiles vary across batches and thereby provide further insight into their technical or biological underpinnings.

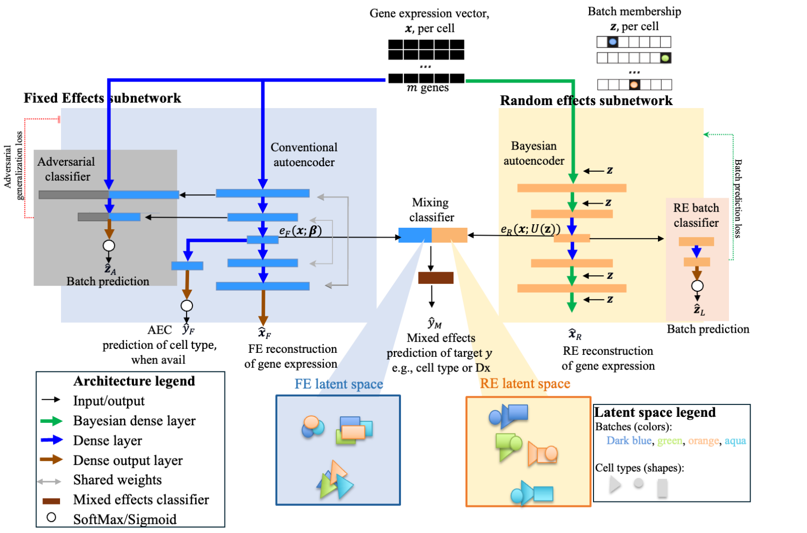

**Fig. 1. Overview and architecture of the scMEDAL framework**. The inputs to the model are the gene expression vector $\boldsymbol{x}$ and the one-hot encoded batch vector $\boldsymbol{z}$ per cell. Both the fixed- and random-effects subnetworks aim to learn a latent representation of the input data which can be used to produce a reconstruction $\hat{\boldsymbol{x}}$ of the input gene expression. The *Fixed Effects subnetwork (scMEDAL-FE)* shown in the blue block, comprises a conventional autoencoder augmented with an adversarial classifier (gray block), which attempts to predict $\boldsymbol{z}$ using features from the AE. To the extent that it can, this classifier penalizes the conventional AE for using such features, encouraging the AE to learn a batch-invariant latent space that preserves reconstruction ability and, hence, cell-type signal $y$. The *Random Effects subnetwork (scMEDAL-RE)* shown in the orange block, includes an autoencoder (orange AE, left) and a parallel MLP classifier designed to predict the batch, $\boldsymbol{z}$. This MLP encourages the scMEDAL-RE to capture batch variability in its latent representation by modeling the effects associated with each batch. Overall, the fixed-effects latent space representation is batch-invariant, while the random-effects latent space captures and enhances batch-specific variability. A classifier, depicted in brown, integrates these latent spaces to predict the labels of interest $\hat{y}$ (e.g., diagnosis or cell type). This enables an assessment of how well the latent spaces preserve biological signals for accurate cell type classification.

By integrating both the FE and RE subnetworks, the scMEDAL framework enables more accurate modeling of biological signals while mitigating the information loss typically incurred by batch correction methods that entirely suppress batch effects. To assess the complementary nature of the latent representations from the subnetworks, we employ a Random Forest classifier[19] to predict target labels, including labels for cell type and for diagnosis (such as ASD *vs.* Typically Developing for the ASD dataset). Additionally, a variation of our framework is developed to exploit any available cell-type labels. This variation replaces the autoencoder network with an autoencoder classifier, which we denote as the *Fixed Effects Classifier subnetwork* (***scMEDAL-FEC***), and which predicts cell type to further enhance the cell-type signal.

To evaluate cell type separability, batch correction, and batch modeling capabilities, we adopt three clustering metrics: the Average Silhouette Width (ASW)[20-22], Calinski-Harabasz (CH) index[21-24], and

Davies-Bouldin (DB) index[21,25]. ASW is our primary metric because its values are bounded from -1 to 1, and it is widely used in scRNA-seq analysis[13,17,26-28], facilitating comparisons across methods and datasets. Additionally, ASW is the most robust of the three metrics, as it effectively captures variations in cluster density, shape, and size. DB is included as it excels at identifying well-separated clusters, while CH is particularly useful for evaluating clustering structure when the number of clusters is well-defined[29].

Once the latent spaces are learned, UMAP[18] is used to assess qualitatively and in an interpretable manner, the extent to which the spaces preserve biologically meaningful information. These projections complement the quantitative metrics discussed above. Additionally, genomaps are used to provide a 2D visualization of individual cell gene expression based on gene-gene interactions. The scMEDAL framework is comprehensively evaluated using three diverse single-cell and single-nucleus RNA-seq datasets, spanning multiple disease conditions, a wide variety of cell types and batch variations. This assessment includes datasets from the cardiovascular system (Healthy Heart), ASD, and AML (Supplementary **Table** S1). Data preprocessing follows standard scRNA-seq practices[28] including UMI count filtering and normalization, log transformation, and selection of highly variable genes. The datasets are then partitioned using five-fold cross-validation, stratified by batch and cell type (see Data preprocessing section 2.2 in the Supplementary information).

### 2.2 scMEDAL subnetworks create complementary batch-invariant and batch-specific latent spaces in the Healthy Heart dataset

The first dataset, the *Healthy Heart dataset*[28,30] consists of scRNA-seq data from tissues of the heart from healthy individuals. It contains multiple sources of batch variability, including two different sequencing protocols (technical effects), samples from various donors, and different tissues within the heart (biological effects). This dataset allows us to assess scMEDAL's ability to handle a high number of batches and complex batch effects. To rigorously evaluate scMEDAL's performance, we compared it with widely used methods for preprocessing scRNA-seq data, including Principal Component Analysis (PCA)[31] and the Autoencoder (AE) neural network[32].

To qualitatively compare the latent spaces learned by these approaches, we apply UMAP[18] to visualize the distribution of 44,987 cells from each model's latent space(s) (**Fig.** 2a). In the first row, which visualizes cell-type separability, both the AE and scMEDAL-FE models display multiple discernible cell types, that are significantly more visible than in PCA's latent space, confirming their efficacy in increasing cell-type separability. When the UMAP is colored by batch, as shown in the second row of **Fig.** 2a, scMEDAL-FE exhibits the most uniform distribution of batch colors, whereas the AE shows many discernible batches (colored regions), confirming its reduced batch separability. The cells labeled as "Not Assigned"—indicating they were too heterogeneous to be assigned to any known singular cell type[33]—are dispersed in the scMEDAL-FE latent representation (**Fig.** 2a, third column, black-colored cells), reflecting their heterogeneity. In contrast, these unassigned cells form a definitive cluster (circled red) in the AE and PCA representations (**Fig.** 2a, first and second columns of first row), which is likely a false positive, since they are not expected to cluster together. This observation indicates that while AE and PCA can amplify signal, they may also amplify noise. Additionally, when using the scMEDAL-FE subnetwork, atrial cardiomyocytes are dispersed alongside ventricular cardiomyocytes. We attribute this to the suppression of batch effects—a combination of variations due to tissue type, donor, and protocol. By eliminating some of the tissue variability information, both types of cardiomyocytes appear to merge. By comparing the AE representation to the scMEDAL-FE representation, we can identify which cell types are batch-invariant, thereby further enhancing our understanding of the cell variability. The findings in both rows of **Fig.** 2a align with the quantitative findings tabulated in **Fig.** 2b and described next.

Our quantitative comparison of the methods has two assessments. In the first assessment, we compare the methods based on batch separability. PCA provides a baseline ASW of −0.48. The proposed scMEDAL framework effectively suppresses batch separability, with the scMEDAL-FE subnetwork attaining an ASW of −0.50 (more negative values indicate less separability), and with the scMEDAL-RE demonstrating its ability to model the batch effect, achieving an ASW of +0.37 (**Fig.** 2b, left columns). Results using additional clustering metrics (CH and DB, Supplementary **Table** S7) further confirm the efficacy of the proposed approach, as do the UMAP visualizations, which show high separability of the batches of scMEDAL-RE (Supplementary **Fig.** S3). These results demonstrate scMEDAL's ability to reduce dimensionality while both suppressing and modeling the batch effects, unlike other approaches, such as the AE, which solely reduce dimensionality. Notably, scMEDAL-FE (−0.50) suppresses batch effects more effectively than the AE (−0.45). In the second assessment, we evaluate the methods based on cell-type separability. The PCA approach yields a baseline ASW score of −0.05. Both the AE and scMEDAL-FE substantially improve cell type separability compared to PCA (**Fig.** 2b, right columns; Supplementary **Table** S7).

**a**

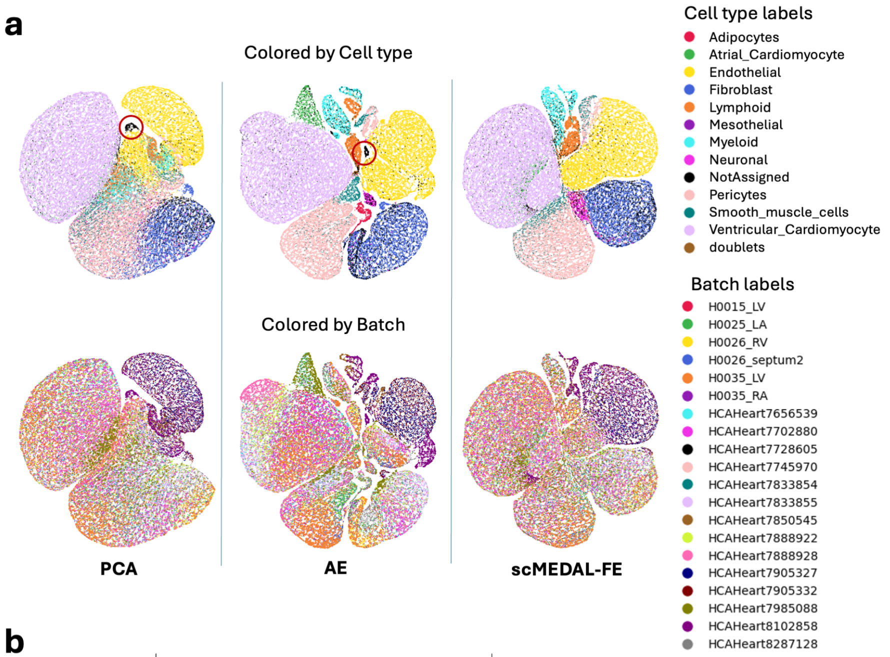

**b**

| | ASW (batch) | | | ASW (cell type) | | |
|---|---|---|---|---|---|---|
| | mean | 95% CI | | mean | 95% CI | |
| PCA (Baseline) | -0.48 | -0.51 | -0.46 | -0.05 | -0.06 | -0.05 |
| AE | -0.45 | -0.48 | -0.43 | **0.19** | **0.15** | **0.23** |
| scMEDAL-FE | **-0.50** | **-0.52** | **-0.48** | **0.16** | **0.09** | **0.23** |
| scMEDAL-RE | **0.37** | **0.16** | **0.58** | -0.28 | -0.45 | -0.11 |

**Fig. 2: scMEDAL subnetworks output complementary batch-invariant and batch-specific latent spaces in the Healthy Heart dataset. a** UMAP visualization of 44,987 cells from 20 out of 147 batches of the Healthy Heart dataset's latent spaces, generated using three models: PCA (first column), AE (second column), and scMEDAL-FE (third column). **b** Average silhouette width (ASW) scores (mean and 95% confidence interval across 5 folds) for batch and cell-type separability in the latent spaces, using the PCA, AE, scMEDAL-FE, and scMEDAL-RE models.

### 2.3 scMEDAL's components reflect disease-associated neuronal patterns in ASD

The second dataset, the *Autism Spectrum Disorder (ASD)*[34], includes single-nucleus (sn) RNA-seq data from brain samples of the prefrontal cortex (PFC) and anterior cingulate cortex (ACC). Batch effects primarily arise from donor variability between ASD and typically developing (control) subjects. There is indirect confounding between cell type and batch, as certain cell types are more affected in ASD than in controls. This dataset enables us to evaluate scMEDAL's capacity to capture donor heterogeneity and disease-related biological variations.

The scMEDAL-FE latent space demonstrates improved the cell-type separability compared to PCA, as evidenced by more clearly distinguishable colored regions (cell types), particularly for myeloid, lymphoid, pericytes, and smooth muscle cells (**Fig.** 3a, top row, first and third columns). Comparing batch separability, although the ASW batch scores of scMEDAL-FE are not more negative than those of PCA (**Fig.** 3b, left columns), we observe that, in the UMAP projection in **Fig.** 3a (bottom row), an equivalent degree of batch mixing (intermingled colors) is apparent between the two methods, confirming that the framework has formed a FE space with suppressed batch effects. Additionally, we observe that the nonlinear properties of the AE component in scMEDAL-FE best preserve the cell type signal (**Fig.** 3b, right columns). The total loss is calculated as the reconstruction loss minus the adversarial loss (as described in the *Methods* section 4.2.1); therefore, increasing the weight of the reconstruction loss reduces the adversarial contribution to the total loss, which can lower batch correction, but enhances the cell-type signal. Our framework uses an scMEDAL-FE subnetwork with a slightly higher reconstruction loss to provide a strong cell-type signal while effectively mitigating batch effects (**Fig.** 3).

Neural cell types that exhibit donor heterogeneity, including variations between ASD patients and controls, can be identified by comparing the latent spaces of the AE and the scMEDAL-FE models on the ASD dataset (**Fig.** 3a, second and third columns). For instance, parvalbumin interneurons (labeled **IN-PV**) —which Velmeshev et al., 2019[34] reported to be dysregulated in patients with ASD—form a distinct cluster (periwinkle color, top row) in the AE latent space, but become dispersed in the scMEDAL-FE representation. This dispersion suggests that the scMEDAL-FE effectively removes donor-specific effects. Similarly, certain excitatory neurons, such as layer 2/3 (labeled **L2/3**, pink color) and layer 5/6 (labeled **L5/6**, beige color) neurons—which are dysregulated in ASD compared to controls—also become dispersed among other layer neurons in the scMEDAL-FE latent space. In contrast, oligodendrocytes (coffee color) and oligodendrocyte precursor cells (labeled **OPC**, navy blue color) exhibit minimal donor heterogeneity, indicating that their latent representations are largely unaffected by donor-specific variability, and are accordingly well separated by the proposed scMEDAL-FE subnetwork. This distinction highlights how the proposed framework can be used to learn a donor-invariant latent space and enhance our understanding of neurobiology.

**a**

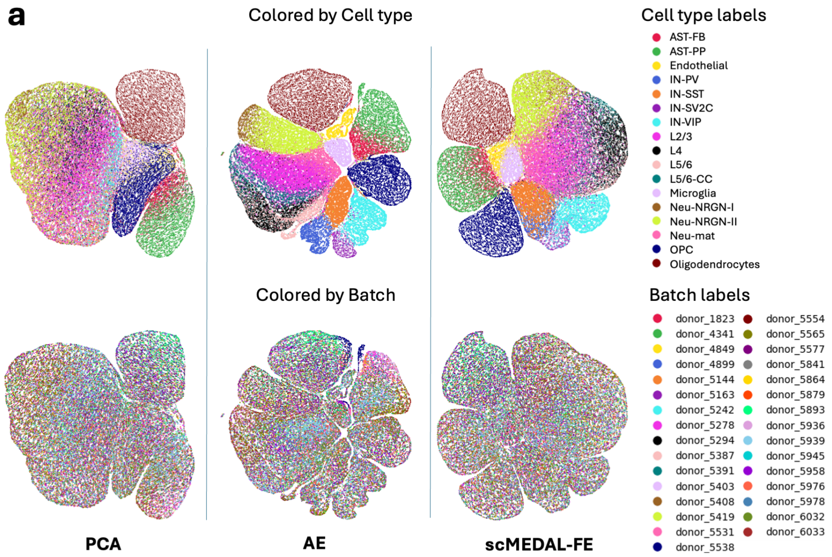

**b**

| | ASW (batch) | | | ASW (cell type) | | |
|---|---|---|---|---|---|---|
| | mean | 95% CI | | mean | 95% CI | |
| PCA (Baseline) | -0.31 | -0.33 | -0.30 | 0.07 | 0.06 | 0.08 |
| AE | -0.22 | -0.23 | -0.20 | **0.33** | **0.31** | **0.36** |
| scMEDAL-FE | -0.23 | -0.26 | -0.21 | **0.20** | **0.16** | **0.23** |
| scMEDAL-RE | **-0.11** | **-0.49** | **0.27** | -0.19 | -0.28 | -0.10 |

**Fig. 3: scMEDAL subnetworks disentangle ASD-related donor effects. a** UMAP visualizations of latent spaces derived from 62,735 cells from 31 donors in the ASD dataset, obtained using PCA (first column), AE (second column), and scMEDAL-FE (third column). **b** Average Silhouette Width (ASW) scores (mean and 95% CI across 5 folds) for batch and cell-type separability in the latent spaces, comparing PCA, AE, scMEDAL-FE, and scMEDAL-RE models.

The scMEDAL-RE shows an increase in the ASW batch separability scores compared to other models, confirming its ability to capture batch (donor) effects as desired (**Fig.** 3b, bottom row). Results using additional clustering metrics (CH and DB) are shown in Supplementary **Table** S8, which further confirm the efficacy of the proposed approach. A UMAP visualization confirming the high batch separability of scMEDAL-RE is shown in Supplementary **Fig.** S4.

### 2.4 scMEDAL balances the trade-off between batch correction and preserving cell type information in leukemia

The third dataset, the *Acute Myeloid Leukemia (AML)*[35], comprises scRNA-seq data from AML-confirmed patients, healthy control individuals, and leukemia cancer cell lines. Batch effects stem from donor variability, including differences between healthy and AML subjects, as well as cell lines. This dataset features strong confounding between cell type and batch: malignant cells are only present in diseased subjects and cell lines, while healthy cells originate from both diseased and healthy patients.

For batch separability, the baseline PCA model achieves an ASW score of -0.28. The AE model fails to suppress batch effects, attaining a less negative ASW of -0.19. Meanwhile, the proposed framework works as intended. The scMEDAL-FE component in our framework further suppresses batch separability to -0.30 (more negative), and the scMEDAL-RE component successfully captures batch variability, with a substantially increased ASW of +0.32 (**Fig.** 4b, left columns). Results using additional clustering metrics, CH and DB, further confirm the superior batch separability provided by scMEDAL-RE's latent space (see Supplementary **Table** S9).

**a**

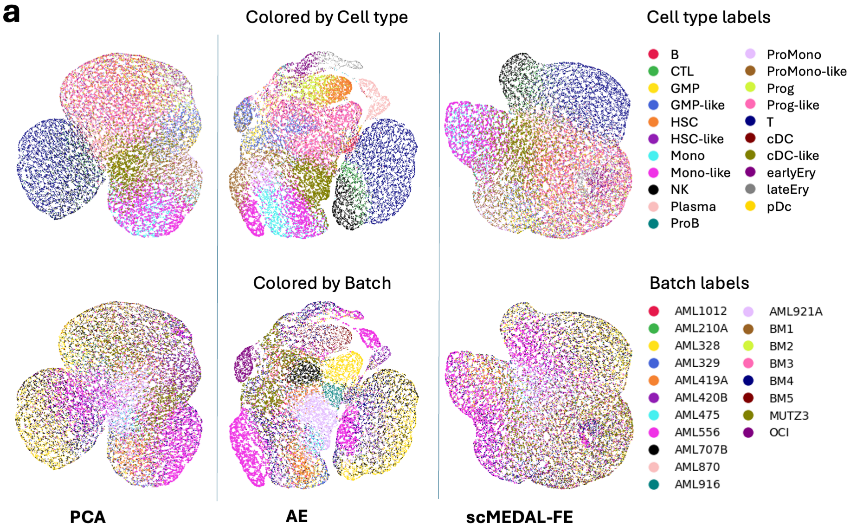

**b**

| | ASW (batch) | | | ASW (cell type) | | |
|---|---|---|---|---|---|---|
| | mean | 95% CI | | mean | 95% CI | |
| PCA (Baseline) | -0.28 | -0.29 | -0.28 | -0.09 | -0.09 | -0.08 |
| AE | -0.19 | -0.21 | -0.17 | **0.03** | **0.02** | **0.05** |
| scMEDAL-FE | **-0.30** | **-0.31** | **-0.28** | -0.05 | -0.07 | -0.04 |
| scMEDAL-RE | **0.32** | **0.23** | **0.42** | -0.36 | -0.45 | -0.28 |

**Fig. 4: scMEDAL-RE captures patient group information complementing the scMEDAL-FE. a** UMAP visualization of latent spaces derived from 23,050 cells and 19 batches in the AML dataset, obtained using PCA (first column), AE (second column), and scMEDAL-FE (third column). **b** Average Silhouette Width (ASW) scores (mean and 95% confidence intervals across 5 folds) for batch and cell-type separability in the latent spaces, comparing PCA, AE, scMEDAL-FE, and scMEDAL-RE models.

This dataset exhibits substantial variation in cell-type representation across donors, introducing a cell-type-to-donor confounding factor (Supplementary **Fig.** S2). Additionally, malignant cell types are present in donors with the disease and in the leukemia cancer cell lines. Consequently, regarding cell-type separability, even though the scMEDAL-FE component of our framework is *more* effective at recovering cell-type signals compared to PCA, it shows a slight reduction in cell-type separability *relative* to the AE (**Fig**. 4b, right columns). This reduction is attributable to the cell-type-to-donor confounding factor; removing batch information leads to the loss of some cell-type information (**Fig.** 4a, second and third columns). Upon further analysis of the latent spaces of the AE and scMEDAL-FE models, it is observed that natural killer (NK), cytotoxic T lymphocyte (CTL), and T cells exhibit greater invariance to donor heterogeneity. Notably, these cell types lack malignant counterparts, which explains their increased invariance in the scMEDAL-FE representation compared to other cell types. This observation underscores the importance of a framework like ours, with its explicit random effects quantification via the scMEDAL-RE subnetwork, which models donor variability suppressed by the fixed effects subnetwork, scMEDAL-FE. As mentioned, scMEDAL-RE effectively captures batch variability, as indicated by the ASW. The latent space of this model further reveals that patients AML328 and AML556, as well as the cell line MUTZ3, exhibit greater heterogeneity compared to other patients and healthy donors (Supplementary **Fig.** S5, shown in yellow, pink, and brown, respectively). This distinction highlights the framework's ability to identify and separate highly heterogeneous batches, thereby enhancing our understanding of the underlying variability within the dataset.

### 2.5 scMEDAL enables deeper understanding of batch effects by addressing retrospective “What if?” questions through generative modeling and visualization

The proposed scMEDAL batch suppression framework offers novel capabilities to address retrospective "What if?" questions. For example, how would a cell's gene expression profile change if it had originated from a different batch? Or, “How would the profile change if it came from a donor with a different diagnosis?”, or “from another donor with the same diagnosis?” Other questions include, “How would the profile change, if the cell came from a cell line rather than a patient?” This section demonstrates the framework's ability to answer these questions.

To highlight the generative capabilities of the scMEDAL framework in addressing critical biological and clinical "What if?" questions, unexplored by traditional methods, we visualize gene expression patterns learned by the scMEDAL-FE and scMEDAL-RE subnetworks. Both subnetworks are used to reconstruct gene expression count matrices. The FE subnetwork transforms input data into a batch-invariant space, revealing gene expression patterns common across all batches. Conversely, the RE subnetwork captures batch-specific variations, enabling the simulation of gene expression profiles as they would appear if cells originated from a different technical or biological batch.

By deliberately altering the one-hot encoded batch vectors input to the RE subnetwork, we can project cells into batch conditions distinct from their original batch (e.g., technical variations, different donors, or diagnoses). This allows exploration of batch variability across donors, diagnoses, or technical acquisition conditions. In the sections that follow, we randomly select 300 cells from specific cell types of interest, while including cells from each batch of interest for the previously described “What-if?” questions. Using the scMEDAL-FE and RE subnetworks, we project their gene expression data. Counterfactual projections of each cell into all batches are generated with scMEDAL-RE. These projections (row vectors) are concatenated (stacked vertically) with the original gene expression vectors to form an overall count matrix, which is standardized. From this matrix, we compute a genomap transform[36] and apply it to visualize gene expression as an image, with each pixel representing a gene and its intensity corresponding to the gene's expression level. An overall genomap transformation is used across all projections, ensuring consistent gene-to-pixel mappings for direct comparison, at the gene (pixel) level. Genomaps for a single cell across batches of interest are plotted to compare gene expression patterns, enabling visual inspection of the effects of batch correction, underlying biological signals, and batch-specific influences.

#### *Healthy Heart Dataset*

In the Healthy Heart dataset, to evaluate our method's effectiveness in learning a generative model of batch effects, we focus on four cell types: pericytes, endothelial cells, fibroblasts, and ventricular cardiomyocytes—across four batches. For each cell type, we select a cell at random to represent the cell type and visualize its gene expression using a genomap (**Fig.** 5, left column). In this genomap, genes (pixels) are arranged in a polar plot, where genes with equivalent gene-to-gene interactions are positioned at the same distance from the image's center (i.e., its origin with radius $\rho = 0$), and the angle $\theta$ is arbitrary. By projecting these cells into different batches, as illustrated in **Fig.** 5, columns 3-6, we examine how batch effects impact gene expression profiles and how these differences vary across cell types.

The first column of **Fig.** 5 shows the original gene expression of the selected cells for each cell type, where distinct expression patterns are observed across the cell types. The second column shows the genomap for the fixed effects reconstruction using the scMEDAL-FE subnetwork, which captures the batch-independent characteristics of the cells. The presence of these distinct gene expression patterns highlights the variability between cell types—independent of batch effects—captured by scMEDAL-FE. In the remaining columns, the following question is explored: If the cells had come from a different batch, how would their gene expression pattern differ? To address this, the cells were projected (reconstructed) as if they had originated from each of four different batches (shown in columns 3-6), including their own batch and three other batches. A red square outline indicates each cell's original (actual) batch. Here, we observe that the scMEDAL-RE subnetwork successfully learned batch-to-batch variability, as demonstrated by the visually distinct genomaps across each row for the same cell. Additionally, within each column, the variability between cell types remains apparent in the differing appearances of the genomaps. Taken together, these results demonstrate that the proposed framework captures both cell type-specific and batch-specific patterns across an array of cell types contained within the Healthy Heart dataset.

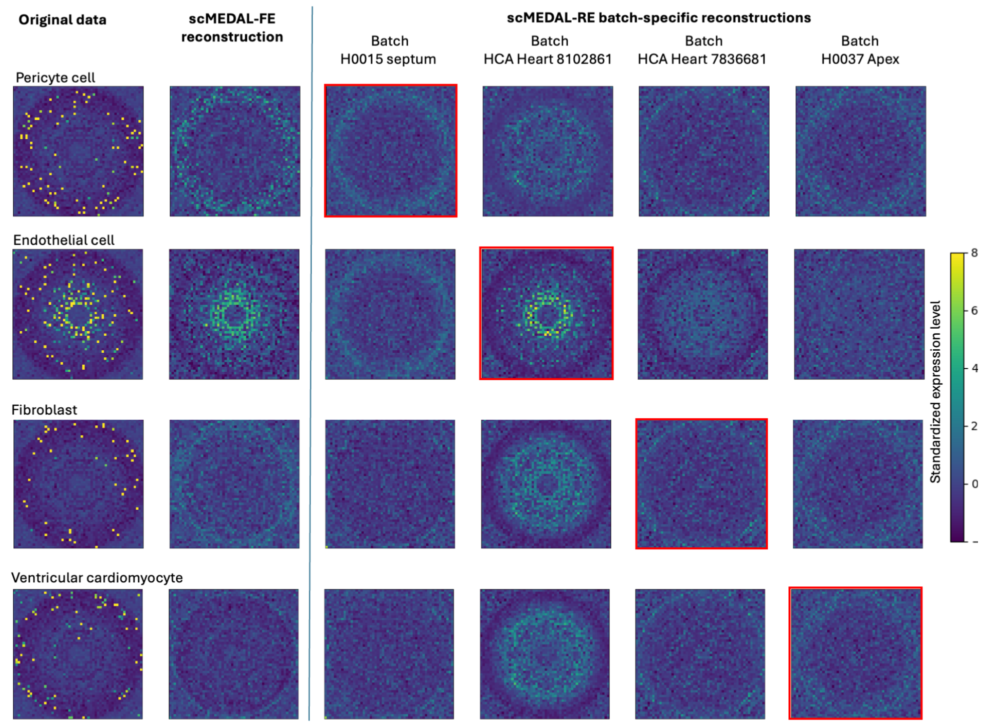

**Fig. 5: How would cells' expression be altered if the cells had originated from different batches in the Healthy Heart dataset?** Genomaps show the gene expression of 2,916 genes for four cells from different cell types across four different batches. The columns show the original expression, the fixed effects reconstruction from scMEDAL-FE, and four counterfactual reconstructions for four different batches using scMEDAL-RE. The red squares highlight the scMEDAL-RE reconstruction for each cell's original batch.

#### *ASD Dataset*

In the ASD dataset, the batch effect arises from donor-to-donor variability. The framework captures both donor-specific and donor-agnostic effects. We investigate differences between autistic and healthy individuals by visualizing L2/3 excitatory neurons from three typically developing (control) donors and three donors with a confirmed ASD diagnosis. We select L2/3 excitatory neurons due to their high number of differentially expressed genes between autistic and control subjects[34]. By projecting cells from healthy donors onto the autistic representation—and vice versa—we elucidate gene expression alterations associated with autism. This approach provides insight into disease-specific gene expression patterns while demonstrating the model's capability to capture and visualize diagnostic conditions.

We visualize gene expression profiles of L2/3 cells using genomaps (**Fig.** 6). In the first column, distinct genomap patterns between cells from control donors (**Fig.** 6, rows 1-3) and autistic donors (**Fig.** 6, rows 4-6) highlight autism-associated differences in gene expression. The second column presents the fixed effects reconstruction using the scMEDAL-FE subnetwork, capturing cell-type variability independent of donor effects. To address the question of how a cell's gene expression pattern would appear if it originated from a different donor—specifically, from a subject with autism versus a control—the cells were reconstructed as if they had come from each of several donors, including their own and others (shown in columns three through eight). We observe that scMEDAL-RE effectively captures donor-specific variability, as evidenced by the varying genomap patterns across each row for the same cell when projected onto different donors. Notably, cells projected onto autistic donors exhibit distinct gene expression patterns, including sets of more actively expressed genes (shown as more pronounced rings), compared to those cells projected onto control donors. The reconstruction corresponding to the cell's original donor is outlined with the red square. These findings demonstrate that the model captures both cell-type–specific and donor-specific patterns, enabling prediction of how gene expression would differ if a cell had come from a different donor, with the same or different diagnosis.

Using the genomap transform of gene expression values, we further analyzed the scMEDAL-RE projection of 300 L2/3 cells from 15 ASD and 16 control donors. Statistical analysis was conducted using linear mixed-effects models. A total of 230 genes were identified as significantly associated with ASD ($p < 0.05$) (SupplementaryData_1.xls, Supplementary **Table** S13). We note that among these, CNR1—encoding the cannabinoid receptor type 1— has been shown to modulate endocannabinoid signaling and impact social reward processing, which is a common deficit in ASD[37]. It has also been shown to influence neurological phenotypes[38]. Another identified gene, CYP27A1, is often mutated in Cerebrotendinous Xanthomatosis (CTX) patients who display autistic traits[39]. Overexpressed KCNJ10 was identified and it has been linked to both ASD and epilepsy[40], aligning with observations that 8 out of 15 ASD patients in the ASD dataset also have epilepsy[34]. MYO1E is also identified and has been highlighted as an ASD candidate gene[41] and THBS1 was identified, which is a gene associated with risk for ASD[42]. Several additional genes were linked to key ASD-related pathways, including NOTCH3, whose alternative signaling affects brain development and synaptic function[43], and IL1R1, which regulates microglial synapse elimination via mTOR signaling[44]. COL4A1 has been associated with epilepsy phenotypes[44], while TGM2 has been implicated in oxidative stress in ASD mouse models, including the commonly used Black and Tan Brachyury (BTBR) mouse model of ASD[45]. Finally, dysregulation of fibroblast growth factors, including the identified FGF2 gene, may drive early brain overgrowth and disrupted connectivity in autism[46].

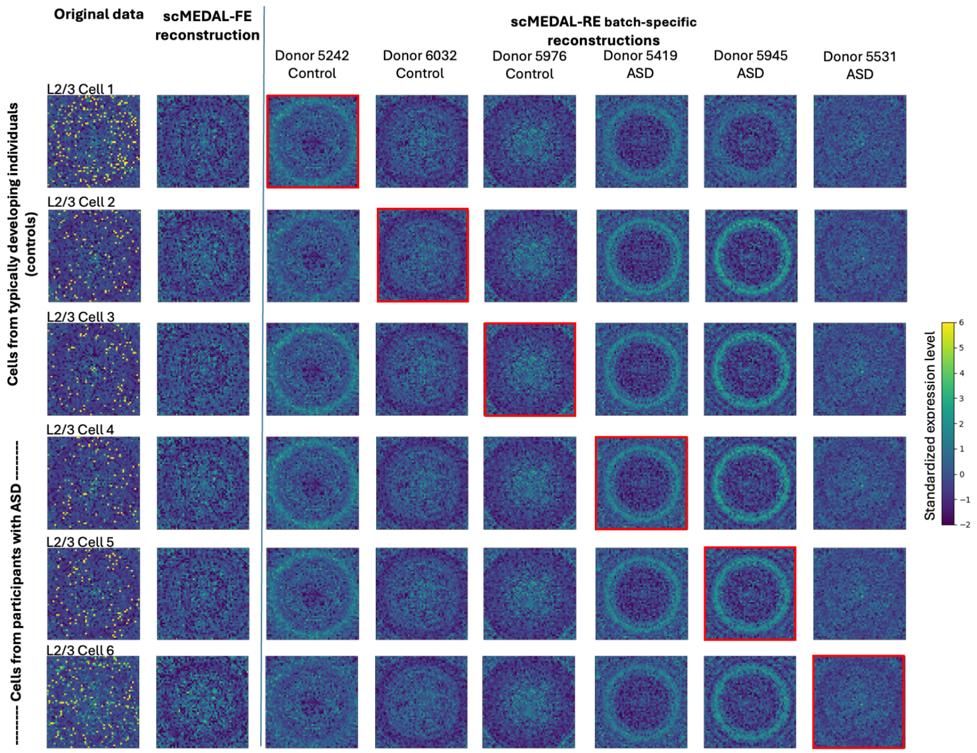

**Fig. 6: How would cells' expression be altered if the cells came from a healthy donor versus a diseased donor in the ASD dataset?** Genomaps show the gene expression of 2,916 genes for six L2/3 cells. The columns depict the cells' original expression, the fixed effects reconstruction from scMEDAL-FE, and six counterfactual reconstructions for six different donors using scMEDAL-RE. The red squares highlight the reconstruction of scMEDAL-RE for each cell's original batch.

#### *AML Dataset*

In the leukemia dataset, the batch effect, also referred to as the patient group, arises from the source of the cells, which includes both donors (diseased and controls) and cell lines. To illustrate how scMEDAL-RE captures disease-related variations, we visualize healthy and malignant monocytes from healthy donors, diseased donors, and cell lines. This ability is crucial for understanding gene expression changes associated with malignancy and for identifying potential therapeutic targets. This experiment assesses our framework's capacity to project and visualize gene expression profile changes under varying conditions, providing unique insights into biological variability and disease mechanisms that prior batch effect suppression methods do not address.

Both normal cells (**Fig.** 7, rows 2 and 4) and malignant cells (**Fig.** 7, rows 1 and 3) are randomly selected for visualization. The first column shows the original gene expression of both normal and malignant monocytes. We observe that while the overall pattern looks similar, there are numerous variations at the level of individual genes, highlighting the natural variability between donors. The second column presents the fixed effects reconstruction using the scMEDAL-FE subnetwork, which captures cell variability independent of the source (i.e., donor or cell line). There is noticeable similarity between the normal cells. In the subsequent columns, the following question is explored: How would a cell's expression pattern vary if it had come from a different donor—specifically from healthy donors, AML donors, or cell lines? To investigate this, both normal and malignant monocytes were reconstructed as if they originated from various donors and cell lines (shown in columns three through five). We observe that the scMEDAL-RE subnetwork effectively captures donor-specific and disease-specific variability, as evidenced by the varying genomap patterns across each row for the same cell, when projected for each batch (columns 3-5). Notably, gene expression patterns differ when normal cells are projected onto AML donors versus healthy donors (e.g., row 2, columns 4-5), and vice versa for malignant cells. This demonstrates the model's ability to predict how gene expression would change if a normal or malignant cell had come from a different donor or cell line. The reconstruction corresponding to the original donor or cell line of each cell is indicated by the red square outline. We observe that our framework has learned distinct gene expression patterns between healthy donors, AML donors, and cell lines. For instance, scMEDAL-RE learned variability specific to the MUTZ3 cell line. MUTZ3 cells have adapted to laboratory growth conditions, and this variability differs fundamentally from that of donor samples, explaining the visual differences observed, when normal and malignant cells from donors (rows 2-4) are projected onto the MUTZ3 cell line batch (column 3) compared to cells from the cell line (row 1). Taken together, these observations underscore the advantage of generative modeling of random effects rather than merely suppressing them, by allowing exploration of how gene expression varies between normal and malignant cells as well as across different donors and batches. Overall, the comprehensive generative scMEDAL-based modeling of both cell-type-specific and donor-specific patterns enhances our understanding of gene expression variability due to disease status and donor differences.

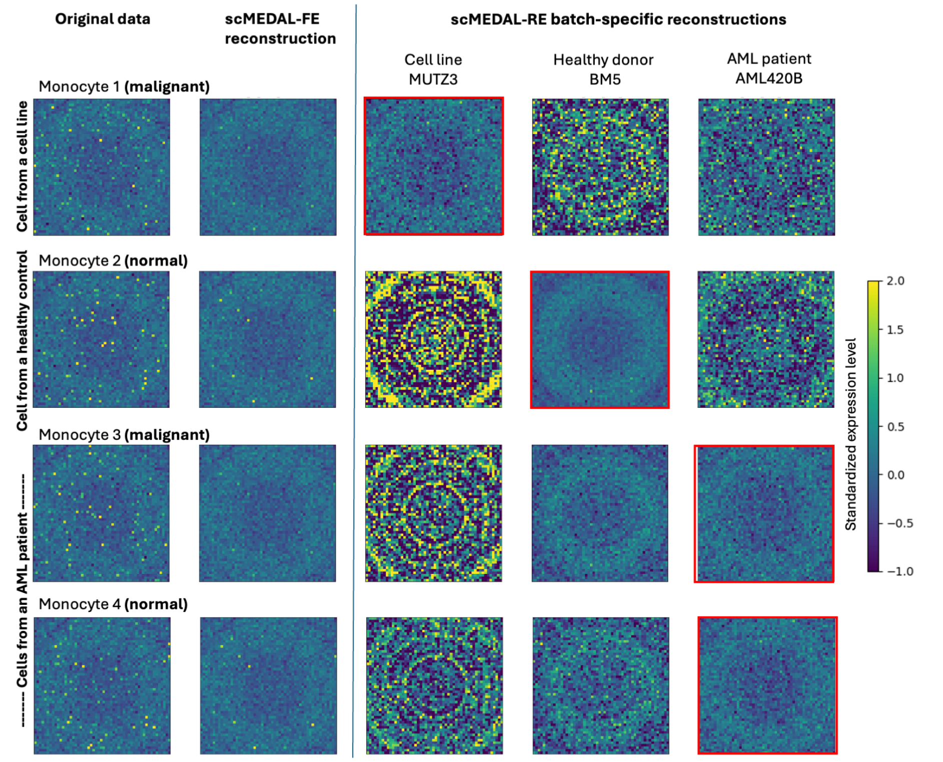

**Fig. 7: How would a cell's expression pattern differ if it had originated from healthy donors, AML donors, or cell lines in the AML dataset?** Genomaps display the gene expression levels of 2,916 genes for normal monocytes (rows 2,4) and malignant monocytes (rows 1,3) across different batches. The illustration includes cells from healthy donors, leukemia patients, and the MUTZ3 cell line. The columns show the original data, the fixed effects reconstruction from scMEDAL-FE, and three counterfactual reconstructions for three different batches using scMEDAL-RE. The red squares highlight the reconstruction of scMEDAL-RE for each cell's original batch.

To explore this capability further, after the genomap transformation, we analyze the expression profiles from 300 monocyte cells for each of the 12 AML (diseased) scMEDAL-RE projections and 5 control (healthy) scMEDAL-RE projections. A Mann-Whitney U test is used to compare diseased and healthy projection distributions, and it identifies 358 genes with significant differential expression ($p < 0.05$) (Supplementary Data 1, Supplementary **Table** S14). Among these, several genes have been established as relevant to AML. SH2B3 plays a critical role in regulating JAK2 signaling, preventing excessive activation of this pathway, which is essential for cell proliferation and differentiation[47]. Mutations in SH2B3 have been implicated in acute lymphoblastic leukemia (ALL)[48] and have also been identified in myelodysplastic syndrome (MDS) patients who have not progressed to AML[49]. The direct role of HERPUD1 in AML remains under investigation. However, it regulates the endoplasmic reticulum (ER) stress response and the unfolded protein response (UPR), processes that likely contribute to AML cell survival[50]. PRDM1, encoding Blimp-1, has been linked to increased leukemia cell counts in AML patients, suggesting its potential as an AML biomarker[51]. MKI67, a proliferation marker, is more frequently expressed in AML cells than in T cells[52]. Additionally, GATA2 deficiency has been associated with an increased risk of AML via MDS progression[53]. Elevated ABCB1 gene expression and protein activity have been identified as indicators of high-risk AML, though they are not directly correlated with drug resistance[54]. Mutations in SAMD9L have also been linked to a high risk of AML development[55]. Additionally, SERPINA1, SLC11A1, and VNN2 are identified, and these are genes associated with both normal and malignant cell types, as reported in Supplementary Table 3 of van Galen et al. (2019)[35]. These findings highlight key molecular alterations that may contribute to AML pathogenesis and provide potential biomarkers for further investigation.

### 2.6 Improved cell classification accuracy using complementary latent spaces of scMEDAL

The scMEDAL's random effects component (scMEDAL-RE) models batch effects quantitatively, rather than discarding them as other batch correction methods do. This improves the framework's ability to predict cell properties, like cell type, patient group, and disease diagnosis. To comprehensively assess the complementary nature of the fixed and random subnetworks, we conducted experiments to quantify the performance of predicting multiple targets across each of our three datasets. Our objective was to quantify the performance that can be attained using the projection from the base model (PCA), and to compare this performance to that attained using only the fixed effects part of scMEDAL, and then to the performance when combining the information from both the fixed and random effects projections of scMEDAL. In this investigation, we train a Random Forest (RF) classifier using three different input sets: (1) the latent space from scMEDAL-FE alone, (2) the latent space from the principal component analysis (PCA) baseline model, and (3) the concatenated latent spaces of scMEDAL (scMEDAL-FE and scMEDAL-RE), which combines the fixed and random effects representations. Prior to training, all latent spaces were standardized, adjusting each to have a mean of zero and a standard deviation of one, to ensure comparability of the inputs.

Regarding the prediction targets, since all three datasets[33-35] included cell type labels, we used cell type as one of our classification targets. In the AML dataset, malignant and healthy cells from the same cell type were treated as two distinct cell-type labels. Additionally, patient group—which includes diseased (AML patients), healthy donors, and cell line classes—was another target to assess the model's capability to differentiate among these groups. For the ASD dataset, we used disease diagnosis (autism spectrum disorder or control) as an additional target. To ensure consistency in our evaluation, we employed the same 5-fold cross-validation splits used to obtain scMEDAL latent spaces for all classification tasks.

We observed that when the RF predictive model of cell type uses latent spaces that *combine* both fixed and random effects of our framework, superior accuracy (ACC = 88.6%, 73.7%, and 60.4%) is achieved across all three datasets (Healthy Heart, ASD, AML) (**Table** 1: first, second, and fourth rows). This *cell-type* prediction performance surpassed that achieved when training on latent spaces derived solely from PCA (ACC = 70.8%, 49.1%, 43.4%) or fixed effects (ACC = 83.5%, 66.9%, 42.7%).

**Table 1. Combining batch-agnostic (scMEDAL-FE) and batch-specific (scMEDAL-RE) latent spaces improves the accuracy of cellular level predictions for all datasets and targets.** The table shows the mean accuracy across 5 folds and the 95% confidence intervals (CI) from the Random Forest Classifier applied using: *cell type* as the primary target (Healthy Heart, ASD, and AML datasets), and *diagnosis* as a secondary target (ASD dataset), and *patient group* as a secondary target (AML dataset). The performance metrics include accuracy, balanced accuracy, and chance accuracy.

| Dataset | Target | Latent space | Accuracy | | | Balanced Accuracy | | | Chance Accuracy | | |
|---|---|---|---|---|---|---|---|---|---|---|---|
| | | | mean | 95%CI | | mean | 95%CI | | mean | 95%CI | |
| Healthy Heart n=486,134 | Cell types (k = 13) | PCA | 70.8% | 70.6% | 70.9% | 37.2% | 37.0% | 37.5% | 16.2% | 16.1% | 16.3% |
| | | scMEDAL-FE | 83.5% | 82.2% | 84.9% | 57.4% | 54.2% | 60.6% | | | |
| | | scMEDAL (FE + RE) | **88.6%** | **88.2%** | **88.9%** | **67.3%** | **65.1%** | **69.4%** | | | |
| ASD n=104,559 | Cell types (k = 17) | PCA | 49.1% | 48.6% | 49.5% | 38.1% | 37.7% | 38.6% | 7.9% | 7.8% | 8.0% |
| | | scMEDAL-FE | 66.9% | 61.5% | 72.2% | 59.1% | 52.0% | 66.1% | | | |
| | | scMEDAL (FE + RE) | **73.7%** | **69.8%** | **77.6%** | **67.0%** | **61.6%** | **72.4%** | | | |
| | Diagnosis (k = 2) | PCA | 52.8% | 52.5% | 53.2% | 52.8% | 52.5% | 53.2% | 50.1% | 49.7% | 50.4% |
| | | scMEDAL-FE | 52.4% | 52.0% | 52.7% | 52.4% | 52.0% | 52.7% | | | |
| | | scMEDAL (FE + RE) | **94.6%** | **89.6%** | **99.6%** | **94.6%** | **89.6%** | **99.6%** | | | |
| AML n=38,417 | Cell types (k = 21) | PCA | 43.4% | 42.6% | 44.2% | 30.3% | 29.5% | 31.0% | 8.0% | 7.9% | 8.1% |
| | | scMEDAL-FE | 42.7% | 40.7% | 44.7% | 30.0% | 28.5% | 31.6% | | | |
| | | scMEDAL (FE + RE) | **60.4%** | **58.0%** | **62.8%** | **47.8%** | **45.1%** | **50.5%** | | | |
| | Patient group (k = 3) | PCA | 75.5% | 75.1% | 75.9% | 51.2% | 50.2% | 52.3% | 60.8% | 60.4% | 61.3% |
| | | scMEDAL-FE | 72.0% | 71.7% | 72.4% | 40.0% | 38.6% | 41.4% | | | |
| | | scMEDAL (FE + RE) | **94.0%** | **87.9%** | **100.0%** | **88.1%** | **76.3%** | **100.0%** | | | |

We additionally sought to determine whether *diagnosis* could be predicted more accurately using both latent spaces. For the ASD dataset, using diagnosis (typically developing *control* versus *ASD*) as the target, the Random Forest classifier achieved the highest performance (ACC = 94.6%) when trained on latent spaces fusing (concatenating) both fixed and random effects from scMEDAL. In contrast, classifiers trained on latent spaces derived from PCA or fixed effects alone barely exceeded chance accuracy, with ACC values of 52.8% and 52.4%, respectively. In the AML dataset, using *patient group* (healthy donor, leukemia donor, or cell line) as the target variable, the Random Forest classifier trained on latent spaces combining the fixed and random effects attained significantly higher performance (ACC = 94.0%), compared to those trained on latent spaces derived from PCA or fixed effects alone, which attained ACC values of 75.5% and 72.0%, respectively. Collectively, the findings of superior accuracy predicting cell type, diagnosis, and patient group targets, underscore the potential utility of scMEDAL to aid in classification tasks using the complementary information extracted from each part of the framework.

### 2.7 The AE classifier, scMEDAL-FEC, enhances cell type preservation

Many transcriptomics datasets include cell-type labels and these can be leveraged by an autoencoder classifier (AEC) to potentially preserve cell-type information in the latent representation while maintaining low reconstruction error and high classification performance. To test this, we investigated how the trade-off between batch effect suppression and cell-type separability in the scMEDAL-FE framework would be affected by replacing the AE with an autoencoder classifier for cell type, creating the scMEDAL-FEC subnetwork. The scMEDAL-FEC subnetwork retains the primary objective of constructing a latent space that is batch-agnostic while simultaneously incorporating a classifier to optimize cell-type predictability. To evaluate its impact, we compare scMEDAL-FEC against the traditional AEC model without separate FE and RE subnetworks. This enables the assessment of whether the effects on cell-type and batch separability are driven by the classifier or the adversarial component that promotes the batch-agnostic representation. Using the same 5-fold cross-validation splits as in scMEDAL, we compute mean adjusted silhouette width (ASW) scores for batch and cell-type, along with their 95% confidence intervals across the folds.

***Healthy Heart dataset***

Integrating a cell type classifier into the base models—resulting in the AEC and scMEDAL-FEC variants—leads to improved mean ASW scores for both batch effects and cell type classification within the Healthy Heart dataset (**Fig.** 8d). Specifically, the mean ASW for cell type classification increased from 0.19 (**Fig.** 2b) to 0.30 with the AEC (**Fig.** 8d, right columns), representing a substantial 58% improvement. For scMEDAL-FEC, this score also increased from 0.16 (**Fig.** 2b) to 0.28 (**Fig.** 8d, right columns), reflecting a similarly impressive 75% improvement. Additionally, although the ASW and CH clustering scores for batch and cell type suggest that the AEC has slightly higher separability scores compared with the scMEDAL-FEC portion of our proposed framework (though this difference is generally not significant in ASW and 1/DB; see **Fig.** 2b and Supplementary **Table** S10), the UMAP projections indicate that scMEDAL-FEC better preserves the biological structure. The scMEDAL-FEC latent space (**Fig.** 8a, right column) more effectively maintains cell type biology than both the AEC (**Fig.** 8a, left column) and scMEDAL-FE models (**Fig.** 2a), and it reduces the number of cells within the "Not Assigned" cluster (black color) compared to the AEC, which is also desirable, as this cell "type" is heterogeneous, not a singular type. Specifically, we observe that including a cell type classifier introduces false positive clustering, which can artificially inflate separability scores. For instance, the AEC (**Fig**. 8, left column) displays a significantly larger cluster of "Not Assigned" cells compared to the AE and PCA models (**Fig.** 2a, black cluster in red circle).

***ASD dataset***

In the autism dataset, incorporating a classifier enhanced the cell type separability of scMEDAL-FE, increasing the mean cell type ASW score from 0.20 (**Fig.** 3b, third row, right columns) to 0.27 (**Fig.** 8d, fourth row), which represents a sizeable 35% improvement in ASW and confirmatory improvements in both 1/DB and CH scores (Supplementary **Table** S8 row 3,columns 3 and 4 *vs* **Table** S11 row 2, columns 3 and 4). Although adding the cell type classifier to the AE model improved its performance, the AEC did not preserve biological features as effectively as the AE alone, as illustrated in the UMAP in **Fig.** 3a. Furthermore, while the AEC model achieves a higher overall score compared to the scMEDAL-FEC model, the proposed framework more effectively preserves biological characteristics (**Fig.** 8b). Specifically, the UMAP shows that excitatory neurons (L cells, purple, black, beige, aqua) are more distinctly defined in the scMEDAL-FEC model, and the boundaries between the L cells and NeuN-NRGN (neurogranin-expressing; brown, lime, pink) cells are more pronounced.

***AML dataset***

For the leukemia dataset, embedding an autoencoder classifier did not change the ASW scores appreciably. For example, the ASW by cell type remained the same at 0.03 and -0.05 for the AE/AEC and scMEDAL-FE/FEC, respectively, as shown in **Fig.** 8d (rows 5 and 6, right columns) versus **Fig.** 4b (rows 2 and 3, right columns). There was some improvement in the CH score for the scMEDAL-FEC, with CH=2065.85, over the scMEDAL-FE with CH=1493.43 (Supplementary **Table** S9 row 3 of column 4 *vs* **Table** S12 row 2, column 4). The ASW scores for batch separability stayed the same for the scMEDAL-FEC at -0.30, while the AEC batch separability improved to about the PCA baseline, though with notably higher variance across cross-validation folds than when using the plain AE. The UMAP projections tell a more nuanced story. Comparing **Fig.** 8c to **Fig.** 4a (right two columns), we observe that there is a noticeable improvement in the cell type separability of both scMEDAL-FEC compared to scMEDAL-FE

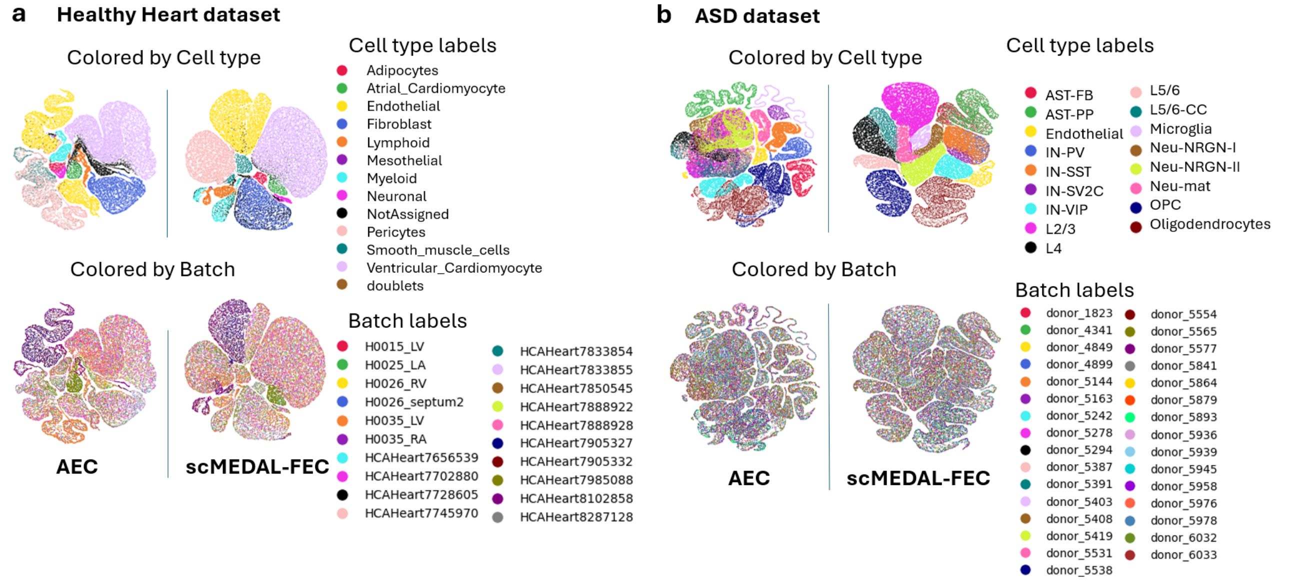

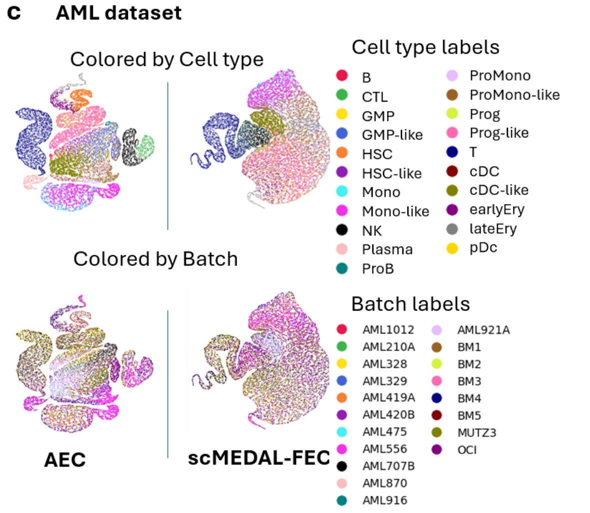

**d Silhouette score analysis**

| dataset | model | ASW (batch) | | | ASW (cell type) | | |
|---|---|---|---|---|---|---|---|
| | | mean | 95% CI | | mean | 95% CI | |
| Healthy Heart | AEC | -0.59 | -0.65 | -0.54 | **0.30** | 0.25 | 0.36 |
| | scMEDAL-FEC | -0.55 | -0.57 | -0.52 | 0.28 | 0.18 | 0.39 |
| ASD | AEC | -0.26 | -0.30 | -0.22 | **0.36** | 0.29 | 0.43 |
| | scMEDAL-FEC | -0.22 | -0.26 | -0.19 | 0.27 | 0.21 | 0.33 |
| AML | AEC | -0.29 | -0.34 | -0.24 | **0.03** | -0.04 | 0.10 |
| | scMEDAL-FEC | -0.30 | -0.37 | -0.24 | -0.05 | -0.08 | -0.02 |

**Fig. 8. Using cell-type labels further improves the preservation of biological information in the scMEDAL-FEC latent space. a-c** UMAP visualizations of gene expression in the latent spaces across different datasets from the AEC and scMEDAL-FEC models. **a** UMAP visualization from 44,987 cells across 20 batches of the Healthy Heart dataset. **b** UMAP visualization computed from 62,735 cells in the ASD dataset. **c** UMAP visualization computed from 23,050 cells in the AML dataset. **d** Avg. Silhouette Width (ASW) scores (mean & 95% confidence intervals across 5 folds) for batch and cell-type separability in the latent spaces of Healthy Heart, ASD, and AML datasets, comparing AEC and scMEDAL-FEC.

and of the AEC compared to the AE, as the cell type colors are more noticeably separated in **Fig.** 8c (top row) compared to **Fig.** 4a (top row, right two columns). This can be understood, in part, because this dataset contains high-quality (high certainty) cell type labels, enabling an embedded AEC to faithfully preserve additional cell type signal. Within the AEC latent space, in particular, clusters representing monocytes (Mono), progenitor cells (Prog), hematopoietic stem cells (HSC), granulocyte-macrophage progenitors (GMP), and conventional dendritic cells (CDC) from both healthy cells and their malignant counterparts (designated with a "-like" suffix, such as Mono-like) are in proximity and, in some instances, overlap (**Fig.** 8c). This result is reasonable, as these cell types are largely similar in their gene expression, differing only in cancer related gene expression. Additionally, the UMAPs (**Fig.** 8c, bottom row) also show that the scMEDAL-FEC method exhibits a superior ability to suppress batch effects compared to the AE and AEC. This improvement is traded off for cell type separability due to confounding between cell type and donor information (Supplementary **Table** S6).

## 3. Discussion

A primary contribution of this work is the development of a novel batch suppression and modeling framework, scMEDAL, which is the first for scRNA-seq analysis to separately characterize and model batch-invariant and batch-specific variations using a mixed effects deep learning approach, complete with modular fixed and random-effects subnetworks. Applying scMEDAL to three datasets with biologically informative batches, we demonstrate effective batch effect suppression while capturing batch-specific variability, enhancing accuracy and interpretability. Analyzing the latent spaces of scMEDAL subnetworks reveals that using only the fixed-effects subnetwork leads to a trade-off between suppressing batch effects and preserving cell-type signals. The random-effects subnetwork mitigates this trade-off by capturing information discarded by the fixed-effects subnetwork, such as batch effects related to tissue, diagnosis, or donor. This dual approach reduces cell-type information loss due to batch-cell-type confounding, while modeling valuable batch effects.

Unlike other batch correction methods (e.g., iMAP[11], ResPAN[12], scDREAMER[13], IMAAE[14], ABC[15], DB-AAE[16]) that focus on creating batch-invariant spaces without including a random-effects subnetwork or explicitly modeling batch distributions, the scMEDAL framework explicitly learns batch distributions via its random-effects subnetwork. The results show that learning batch distributions is necessary to recover information discarded by the fixed-effects subnetwork. Additionally, comparing our fixed-effects subnetwork with an autoencoder allowed introspective analysis of latent spaces, identifying cell types with the most heterogeneity across batches. In the Healthy Heart dataset, the fixed-effects subnetwork prevented misleading clustering of "not assigned" cell types seen in PCA and autoencoders, thereby avoiding false clustering and misidentification.

A second contribution of this work is enabling the answering of retrospective "What if?" analyses by leveraging the generative nature of the framework's fixed and random effects subnetworks to predict a cell's expression under different batches or diagnoses, in addition to the batch agnostic expression. Using the learned, modeled random effects, we reconstructed a cell's gene expression under different conditions to answer “What if?” questions. For example, we predicted how a cell's profile would change if it originated from a different batch, highlighting variability within and between cell types. In the ASD and AML datasets, we simulated how cells would appear from donors with different diagnoses, providing insights into disease and donor variability. Our model's ability to project cells across batches represents a significant advancement in personalized single-cell modeling. Models like AIF[56] incorporate batch modeling but do not explicitly separate batch effects. By isolating batch variability, our approach enhances explainability and facilitates the disentanglement of batch effects. The scMEDAL framework is the first to exploit cell projections for addressing such retrospective scenarios (“What if?” questions) and to separate fixed and random effects in scRNA-seq data using deep learning.

Historically, evaluation of batch correction methods has focused on clustering metrics[4,5,57,58] rather than classification performance. However, we hypothesized that predictive models, which learn complementary batch-agnostic and batch-specific latent spaces, would enable more accurate cell-level predictions. Our third contribution is demonstrating that the proposed framework provides substantial classification improvement by combining the fixed and random effects representations. This combination of latent space information outperformed both PCA latent space representations, as well as the fixed-effects latent space alone, across all datasets tested and across multiple targets including cell-type and diagnosis classification. These results confirmed that the fixed-effects and random-effects latent spaces contain complementary information which can improve cell-level classification accuracy. The fixed effects subnetwork, scMEDAL-FE, provides our fourth contribution: preventing false-positive cell clustering, which is typically observed in PCA and traditional autoencoder-based processing in the presence of label uncertainty.

Our fifth contribution is preserving cell-type biology in the latent space by adding a cell-type classifier to the fixed-effects subnetwork, for those cases when cell-type labels are available. We incorporated this classifier into scMEDAL-FE to maintain cell-type signals within the batch-invariant space. Methods like AutoClass[10], scDREAMER[13], and ABC[15] include cell-type classifiers in their supervised approaches for batch effect suppression and preservation of biology information. However, using a cell-type classifier requires accurate labels, and may overlook annotation errors. Low-quality or uncertain labels can lead to spurious clustering and incorrect interpretations, as we observed in the AEC

model, but mitigated through our supervised subnetwork, scMEDAL-FEC. We note that preserving cell-type signals remains challenging in settings where cell-type and batch effect are strongly confounded (e.g., in the AML dataset; see Supplemental **Fig**. S2), even when using scMEDAL-FEC. In such scenarios, explicitly modeling batch variability with a random effects subnetwork is essential for retaining information that would otherwise be lost through batch suppression.

One limitation of this work is the use of a two-dimensional latent space; assessing how performance varies with the use of higher-dimensional projections remains a topic for future work. Additionally, our framework requires hyperparameter optimization, such as for the weights assigned to loss function terms for the subnetworks; however, in practice we have found that strategies such as grid search suffice to readily find weights that balance the loss terms well.

The scMEDAL framework is modular, allowing for independent training of both the fixed (batch effect suppression) and Bayesian random effects subnetworks. This allows users to selectively replace parts of our framework with their own batch effects suppression approach, such as AutoClass[10], DESC[21], scDREAMER[13], while retaining and leveraging scMEDAL's overall framework, random effects subnetwork, and unique visualization capabilities. Users can incorporate different fixed-effects networks, allowing integration with other batch-invariant models while pairing with the scMEDAL random-effects subnetwork. This flexibility could further enhance performance and is an avenue for future exploration. The above contributions, together with the comprehensive framework evaluation across diverse single cell and single nucleus RNA-seq datasets spanning multiple health conditions, cell-types, and batch effects—including datasets from the cardiovascular system (Healthy Heart), ASD, and AML, underscore the value of separately modeling and quantifying fixed and random (batch) effects through generative deep learning for interpretable transcriptomics analysis.

## 4. Methods

### 4.1 Mathematical foundation of the scMEDAL framework

When data is confounded by batch effects, as commonly observed in single-cell RNA sequencing (scRNA-seq) data, it is essential to employ methods that account for the confounding, otherwise cell types can become mislabeled and transcriptomic impacts of diseases can be misconstrued. The scMEDAL framework addresses this need to mitigate batch confounding. The framework is built upon the mathematical foundation of the linear mixed-effects (LME) model [59]. The LME model is defined as:

$$y_i = \beta_0 + \boldsymbol{x}_i^T\boldsymbol{\beta}_1 + u_{j,0} + \boldsymbol{x}_i^T\boldsymbol{u}_j + \epsilon_i \qquad (1)$$

where $y_i$ is the target prediction for the $i^{th}$ sample which is a cell in our application, $x_i^T$ is the observed gene expression vector of the $i^{th}$ cell, while $\beta_0$ and $\boldsymbol{\beta}_1$ denote the fixed effects scalar intercept and slope vector, respectively, which capture batch invariant trends across all samples. Batches (technical and biological) cluster the cells' expression, causing greater correlation among cells within the same batch than between batches. In the mixed effects model, the $i^{th}$ cell comes from the $j^{th}$ batch. The random effects intercept and slope vector are denoted $u_0$ and $\boldsymbol{u}_{j,0}$ respectively, and are assumed to follow a random distribution, most often modeled as a multivariate normal distribution with mean 0 i.e., $\boldsymbol{u} \sim N(0, \boldsymbol{\Sigma}_j)$. The residual error term of the $i^{th}$ sample is denoted $\epsilon_i$. The proposed scMEDAL framework implements all the components of the mixed effects foundation using neural networks, resulting in a novel model capable of learning both linear and nonlinear relationships between $\boldsymbol{x}_i^T$ and $y_i$. Additionally, it mitigates the confounding batch (clustering) effects by separately quantifying both fixed and random effects, enhancing model interpretability, data understanding, and cell-level predictions.

### 4.2 The Bayesian neural network architecture of the scMEDAL model

The following sections present a description of the scMEDAL framework components, which quantitatively and separately model both fixed and random effects within gene expression vectors of individual cells. The framework's architecture is comprised of two parallel autoencoder subnetworks. The

*fixed effects subnetwork* includes the first autoencoder, a conventional autoencoder neural network (**Fig.** 1, blue area) and an adversarial classifier $A$, (**Fig.** 1, gray area) and together they learn the batch-invariant features. The *random effects subnetwork* includes the second autoencoder, a *Bayesian* autoencoder with probabilistic weights, (**Fig.** 1, orange area) which learns the batch-dependent features. The framework also includes a learned mixing function $f_M$ (denoted mixed effects classifier) that combines the fixed and random effects for prediction. The weights within scMEDAL are learned from the training data partition, which includes a gene expression count matrix and a batch design matrix. The gene expression count matrix is $\boldsymbol{X} \in \mathbb{R}^{n \times g}$, where $n$ is the number of cells in the training partition, and $g$ is the number of genes. The $i^{th}$ row of $\boldsymbol{X}$ contains the gene expression, $\boldsymbol{x}_i$, of the $i^{th}$ cell. To encode batch membership information for the $n$ cells and $K$ batches, we introduce a one-hot encoded design matrix $\boldsymbol{Z} \in \mathbb{R}^{n\times K}$, where $\boldsymbol{Z}_{i,j} = 1$ if sample $i$ belongs to cluster $j$ and $\boldsymbol{Z}_{i,j} = 0$ otherwise. The one hot encoding of the $i^{th}$ cell is the $i^{th}$ row, $\boldsymbol{z}_i$, from matrix $\boldsymbol{Z}$. Each subnetwork learns a lower dimensional latent representation of $\boldsymbol{x}_i$ and outputs a gene expression $\hat{\boldsymbol{x}}_i$ reconstructed from its latent space representation.

#### 4.2.1 Fixed Effects Subnetwork

We start by describing the architecture of the conventional autoencoder (**Fig.** 1, blue area), which contains an encoder to compress a gene expression vector $\boldsymbol{x}$ into a latent, **F**ixed effects representation $e_F(\boldsymbol{x};\boldsymbol{\beta})$, where $\boldsymbol{\beta}$ contains all learned weights up to and including the latent representation. A decoder then reconstructs the gene expression vector $\hat{\boldsymbol{x}}_F$. The encoder comprises three blocks, each containing two dense layers. The output of the final encoder block is passed through a dense layer with two neurons, generating the compressed latent representation. All layers, except the decoder's output layer, use the Scaled Exponential Linear Unit (SELU) activation function. The decoder follows an architecture symmetric to the encoder, also consisting of three layers, with a linear activation applied in the output dense layer. For the supervised fixed effects subnetwork *variant*, we assume cell type information is available for training and test its efficacy. In this scenario, to simultaneously perform cell type classification, we introduce an auxiliary classifier subnetwork (**Fig.** 1, middle of blue area) which predicts the cell type, $\hat{y}_F$, from the latent representation. This auxiliary classifier takes the encoder's latent representation as input and contains a dense hidden layer and a softmax output layer.

To create the fixed effects subnetwork, which is a domain adversarial autoencoder that we denote as scMEDAL-FE, we add an adversarial classifier $A$, (**Fig**. 1, gray area), to predict each cell's batch $\hat{\boldsymbol{z}}_A$ from the layer activations of the encoder. To the extent that it can predict the batch (i.e., $\hat{\boldsymbol{z}}_A = \boldsymbol{z}$) it penalizes the FE autoencoder for using batch-dependent features. Thus through a generalization loss, the autoencoder is penalized for learning features that allow accurate batch prediction. Note that the encoder and decoder weights are tied so that this penalty affects both modules of the autoencoder. The adversary uses the same architecture as the encoder, with the addition of a final softmax output layer. The overall loss function $\mathcal{L}_{FE}$ of the fixed effects subnetwork consists of this categorical cross entropy penalty term and a reconstruction error term which is expressed as:

$$\mathcal{L}_{FE}(\boldsymbol{X},\boldsymbol{Z}) = \lambda_{recon,F}\mathcal{L}_{MSE}\big(\boldsymbol{X},\widehat{\boldsymbol{X}}\big) - \lambda_A\mathcal{L}_{CCE}\big(\boldsymbol{Z},\widehat{\boldsymbol{Z}}\big) \qquad (2)$$

where the hyperparameters $\lambda_{MSE}$ and $\lambda_A$ control the relative weight of the reconstruction and generalization loss, respectively. The reconstruction error is measured as the mean squared error (MSE):

$$\mathcal{L}_{MSE}\big(\boldsymbol{X},\widehat{\boldsymbol{X}}\big) = \frac{1}{n}\sum_{i=1}^{n}\big|\big|\boldsymbol{x}_i - \hat{\boldsymbol{x}}_i\big|\big|^2 \qquad (3)$$

where $\boldsymbol{x}_i$ and $\hat{\boldsymbol{x}}_i$ are the original and reconstructed gene expression vectors of cell $i$. The categorical cross-entropy (CCE) quantifies the adversarial classifier's performance:

$$\mathcal{L}_{CCE}\big(\boldsymbol{Z},\widehat{\boldsymbol{Z}}\big) = -\frac{1}{n}\sum_{i=1}^{n}\sum_{k=1}^{K}\boldsymbol{z}_{i,k}\log\hat{\boldsymbol{z}}_{i,k} + \big(1-\boldsymbol{z}_{i,k}\big)\log\left(1-\hat{\boldsymbol{z}}_{i,k}\right) \qquad (4)$$

where $\boldsymbol{z}_{i,k}$ is the true label (one-hot encoded) and $\hat{\boldsymbol{z}}_{i,k}$ is the predicted probability for class $k$ for cell $i$. Minimizing the fixed effects loss, $\mathcal{L}_{FE}$, enables this subnetwork to suppress batch-specific variations and capture batch-agnostic fixed effects.

#### 4.2.2 Random Effects Bayesian Subnetwork

The random effects subnetwork (scMEDAL-RE) specifically models the batch-dependent effects. Because gene expression batch effects are believed to pervade all levels of the feature hierarchy, including lower gene-level features as well as higher-level gene abstraction features, we construct the random effects subnetwork (**Fig.** 1, orange area), as a second, autoencoder whose depth mirrors the FE conventional autoencoder. However, this second autoencoder, is a *Bayesian* network which we denote as scMEDAL-RE, and thus it has probabilistic weights. This Bayesian autoencoder consists of dense hidden layers in its encoder and decoder components (**Fig.** 1, left side of orange box). The input consists of the standardized expression matrix, $\boldsymbol{X}$, for cells in the training set, and the label matrix for the cells indicating their batch, $\boldsymbol{Z}$, with additional connections providing $\boldsymbol{Z}$ directly to each layer. The autoencoder learns a latent representation which can be used to faithfully reconstruct the input as its output. The RE subnetwork also includes a batch classifier network (**Fig.** 1, right side of orange box) which predicts the batch label $\boldsymbol{z}$ for each cell using the latent representation of the RE autoencoder. To the extent that it can predict the batch (i.e., $\hat{\boldsymbol{z}}_L = \boldsymbol{z}$) it rewards the RE autoencoder weights that construct that latent representation.

The Bayesian random effects subnetwork is a Bayesian autoencoder, $\hat{\boldsymbol{x}}_R = h_R(\boldsymbol{x}; U(\boldsymbol{z}))$, which encodes a given cell's gene expression, $\boldsymbol{x}$, into a lower dimensional, batch effect-laden, latent representation, $e_R(\boldsymbol{x}; U(\boldsymbol{z}))$, and then decodes that representation back to a reconstruction, $\hat{\boldsymbol{x}}_R$, which closely approximates $\boldsymbol{x}$. Collectively we represent the probabilistic weights of all the layers the autoencoder as $U(\boldsymbol{z})$ and we note that these weights in general, depend on the batch from which the cell came, $\boldsymbol{z}$. The Bayesian approach entails finding these weights, $U$, that are most likely given the observed genomic data $\boldsymbol{X}$. This entails finding the weights that maximize the posterior distribution $p(U \mid \boldsymbol{X})$, for all the cells in the training set, $\boldsymbol{X}$. In scMEDAL, it is learned through variational inference (VI), which casts the probabilistic Bayesian modeling as an optimization problem that can be solved efficiently through gradient descent.[60] The goal of VI is to find an approximation to $p(U \mid \boldsymbol{X})$, which is called a surrogate posterior, $q(U)$. We want $q(U)$, which we model as a multivariate normal distribution, to be as close as possible to $p(U \mid \boldsymbol{X})$ while still being computationally tractable. Closeness between two distributions can be measured with the Kullback-Leibler (KL) divergence:

$$D_{\mathrm{KL}}(q(U) \parallel p(U \mid \boldsymbol{X})) = \mathbb{E}_{q(U)}\left[\log\frac{q(U)}{p(U|\boldsymbol{X})}\right] \qquad (5)$$

Using Bayes rule we can expand the posterior in the expectation, $p(U \mid \boldsymbol{X}) = \frac{p(X|U)p(U)}{p(\boldsymbol{X})}$, however we cannot compute all its factors, because of the intractable marginalization $p(\boldsymbol{X})$ which requires integrating over all possible values of $U$. VI addresses this issue by maximizing the Evidence Lower Bound (ELBO), which provides an approximation to the true posterior $p(U \mid \boldsymbol{X})$. After expansion and regrouping terms, the ELBO constraint is derived and it identifies the surrogate posterior which maximizes the log-likelihood of the data, and contains entirely tractable terms:

$$\mathrm{ELBO} = \mathbb{E}_q[\log\, p(\boldsymbol{X} \mid U)] - D_{\mathrm{KL}}(q(U) \parallel p(U)) \qquad (6)$$

where the first right-hand term is the expected log-likelihood of the gene expression data and the second term is the KL divergence between the surrogate posterior and the prior over model weights.[60] The scMEDAL framework is a deep learning neural network, which is optimized via gradient *descent*. To walk downhill, we minimize the negative ELBO. The first term is the data fidelity term, $E_{q(U)}[log\ p(\boldsymbol{X}|U)]$, which measures how well the model fits the data, which we measure as the reconstruction error, $\mathcal{L}_{MSE}(\boldsymbol{X}, \widehat{\boldsymbol{X}})$. After substitution, the objective loss function for the RE subnetwork is:

$$\mathcal{L}_{recon,R}(\boldsymbol{X}, \widehat{\boldsymbol{X}}) + \lambda_K D_{\mathrm{KL}}(q(U) \parallel p(U)) \qquad (7)$$

where $\lambda_K$ is a hyperparameter that controls the degree of regularization from the second term.

The random effect weights of scMEDAL-RE are learned layer-wise using Bayesian, random effects dense (REDEN) blocks (Supplementary **Fig.** S1). REDEN blocks supplant the layers of the original autoencoder. They begin with a dense layer, which takes as input the learned representation output from the previous layer $\boldsymbol{x}_{l-1}$ and output the learned representation $\boldsymbol{d}_l$. Then a learned batch-specific random slope, $\gamma(\boldsymbol{z})$, and batch-specific bias, b($\boldsymbol{z}$), is applied to $\boldsymbol{d}_l$, effectively rescaling and shifting the dense layer outputs in a batch-dependent manner, into a new transformed learned representation, $\boldsymbol{x}_l$. These batch-specific slopes $\gamma(\boldsymbol{z})\sim N(0, \sigma_\gamma)$ and biases b($\boldsymbol{z}$)$\sim N(0, \sigma_b)$ are regularized to follow normal distributions, with mean zero, and learned variances $\sigma_\gamma$ and $\sigma_b$, respectively.

To further enforce the learning of batch-specific features in scMEDAL-RE, we add a RE batch classifier (Fig. 1 orange area, right side) which predicts cluster membership $\hat{\boldsymbol{z}}_L$ from the latent representation. By minimizing the prediction error the classifier, scMEDAL-RE is encouraged to produce latent representations and hence reconstructions that characterize the batch effects. Combining these components, the total loss function for the random effects subnetwork $\mathcal{L}_{RE}$ becomes:

$$\mathcal{L}_{RE}(\boldsymbol{x}, \widehat{\boldsymbol{x}}_R, \boldsymbol{z}, \hat{\boldsymbol{z}}_L,) = \lambda_{recon,R}\mathcal{L}_{MSE}(\boldsymbol{x}, \widehat{\boldsymbol{x}}_R) + \lambda_L\mathcal{L}_{CCE}(\boldsymbol{z}, \hat{\boldsymbol{z}}_L) + \lambda_{KL}D_{KL}(q(U) \parallel p(U)) \qquad (8)$$

where $\lambda_{MSE}$, $\lambda_{CCE_z}$and $\lambda_{KL}$ are hyperparameters weighting each loss term. The first term encourages accurate reconstruction of the data, the second term ensures that the latent space captures batch-specific information, and the third term regularizes the model by aligning the learned distribution $q(U)$ with the prior $p(U)$, thereby minimizing overfitting.

#### 4.2.3 Overall framework

The overall framework, scMEDAL, integrates the fixed effects subnetwork (scMEDAL-FE) and the random effects subnetwork (scMEDAL-RE). In section 2.5, we demonstrate the complementarity of the latent spaces learned by these models by concatenating the latent representations $e_F(\boldsymbol{x};\beta)$ and $e_R(\boldsymbol{x}; U(\mathbf{z}))$ into a single vector and used this as input to a separate classifier trained to infer a specific target label, $\hat{y}_M$, e.g., the cell type or the diagnosis (malignant or benign) for the cell. The objective of the overall scMEDAL framework is:

$$\begin{aligned} &\lambda_{recon,F}\mathcal{L}_{MSE}(\boldsymbol{x}, \hat{\boldsymbol{x}}_F) + \lambda_{recon,R}\mathcal{L}_{MSE}(\boldsymbol{x}, \hat{\boldsymbol{x}}_R) \\ &\quad + \lambda_y\mathcal{L}_{CCE}(y, \hat{y}) \\ &\quad + \lambda_K D_{KL}(q(U) \parallel p(\boldsymbol{U})) \\ &\quad + \lambda_L\mathcal{L}_{CCE}(\boldsymbol{z}, \hat{\boldsymbol{z}}_L) \\ &\quad - \lambda_A\mathcal{L}_{CCE}(\boldsymbol{z}, \hat{\boldsymbol{z}}_A) \end{aligned} \qquad (9)$$

where the *first line* in the overall objective contains the two data fidelity terms for the FE and RE subnetworks which characterize the reconstruction loss and ensure that the latent spaces have information which can be used to faithfully reconstruct the gene expression data. We use the mean squared error (MSE) between the input cell $\boldsymbol{x}$ and its reconstruction $\hat{\boldsymbol{x}}$. The *second line* contains the cell type classification loss (categorical cross entropy) between the true and predicted cell type labels $y$ and $\hat{y}$, for section 2.6 when cell type labels are assumed available. The *third line* is the KL divergence regularization term for Bayesian layers which comes from the derivation of the ELBO constraint of variational inference. The fourth *line* contains the categorical cross entropy between the true cluster label

$\boldsymbol{z}$ and the cluster labels predicted by the latent cluster classifier $\hat{\boldsymbol{z}}_L$ which encourages the RE latent space representation to be predictive of the batch. The *last line* contains the cluster generalization loss which encourages the fixed effects subnetwork to learn features that prevent the adversary from predicting batch labels $\hat{\boldsymbol{z}}_A$ and thereby enabling the FE subnetwork to learn a batch agnostic latent representation. The scMEDAL hyperparameters are listed in Supplementary **Tables** S2–S4. All AEC models were trained with the Adam optimizer with a learning rate of 0.0001 for 500 epochs. Early stopping, based on the reconstruction mean squared error (MSE) on validation data, was applied with a patience of 30 epochs as a regularization strategy to prevent overfitting.

### 4.3 Experiments and model comparisons

We rigorously evaluate the performance of the scMEDAL framework by comparing it to widely used models in scRNA-seq analysis, as well as to variations of the scMEDAL framework itself. The evaluations span conditions (autism, leukemia, cardiovascular), cell types, as well as technical and biological effects, providing a comprehensive assessment of the proposed framework. The model we first compare against is the principal component analysis PCA[31], a standard and widely-used, linear method for dimensionality reduction in scRNA-seq analysis, whose properties are well understood, and it therefore served as a solid baseline for comparison. We then compare against a conventional autoencoder (AE)[32], which is a nonlinear dimensionality reduction technique widely applied in transcriptomics. The AE both complements the PCA method and serves as an ablation test for our autoencoder-based scMEDAL-FE model. To effectively determine how well the scMEDAL subnetworks learn complementary batch-invariant (scMEDAL-FE) and batch-specific (scMEDAL-RE) representations, we conduct multiple experiments described in the following sections.

#### 4.3.1 Experiment 1: Characterize cell type separability, batch correction and visualize the learned batch effects

To evaluate the performance and interpretability of the scMEDAL framework, quantitative and qualitative assessments of the learned representations of the two subnetworks are conducted. In the *quantitative* assessment, we measure cell type separability and batch correction by determining how well each subnetwork transforms input gene express data into batch-invariant and batch-specific latent spaces, representations formed in the bottleneck layer of each subnetwork. To evaluate the batch-invariant space from the fixed effects subnetwork, we compute its clustering *by cell-type* using three measures: ASW[20],CH[23] and the reciprocal of DB[25]. Higher clustering scores indicate better cell type separability. We assess the suppression of batch effects by computing the latent space's clustering *by batch*, where lower scores denote more effective suppression of batch effects. In contrast, to evaluate the batch-specific latent space of the random effects subnetwork, we compute this latent space's clustering by batch, where an increase in clustering scores indicates successful modeling of the batch effects.

In the *qualitative* assessment, we characterize the batch-invariant and batch-specific latent spaces through 2D UMAP visualizations of the cells projected into each space. Coloring by batch or cell-type allows us to directly assess the quality of the clustering through visual inspection. We also characterize the latent spaces by generating 2D images, called genomaps, which depict gene expression for individual cells, with pixel intensity corresponding to gene expression level. Images are reconstructed from the batch-invariant latent space of scMEDAL-FE, and additional genomap images are constructed by leveraging the generative properties of the autoencoder, from the batch-specific latent spaces of scMEDAL-RE. Since this subnetwork learns to model the batch effects for each batch, we can use it to address retrospective "What if?" questions regarding what a cell's gene expression profile would look like if it had originated from a different batch, i.e., a different technical batch or biological donor. By intentionally setting the one-hot encoded batch vectors in the RE subnetwork, we project cells into different batches conditions. This projection capability enables exploration of the variability between different donors, or diagnoses, or technical data acquisition conditions. The results of this assessment are described in Section 2.5.

#### 4.3.2 Experiment 2: Evaluate how the complementary nature of scMEDAL's latent representations enhances prediction performance

The aim of this experiment is to evaluate the complementary nature of scMEDAL's latent representations and its ability to improve label prediction accuracy. This experiment explores the extent to which quantitatively modeling batch effects, rather than discarding them as other batch correction methods do, can improve predictions about the cells. To assess the complementary nature of the fixed and random effects latent spaces, we utilize the latent representations from scMEDAL-FE and scMEDAL-RE obtained in Experiment 1. Then we train a Random Forest classifier[19] using three different input sets: (1) the latent space from scMEDAL-FE, (2) the latent space from the principal component analysis (PCA) baseline model, and (3) the concatenated latent spaces of scMEDAL-FE and scMEDAL-RE, which combines the fixed and random effects representations. The Random Forest model was chosen because it is interpretable, effectively handles both linear and nonlinear relationships, and it is not prone to overfitting. We used the same 5-fold cross-validation partitioning scheme described for preprocessing the data input for the scMEDAL subnetworks. The PCA latent space was also utilized as a comparative baseline. This approach enabled us to assess how the integration of both fixed and random effects enhances classification performance compared to using fixed effects alone. Since all three datasets[33-35] included cell type labels, we used cell type as the primary target for classification. In the AML dataset, we also conducted experiments using patient group as a target, while in the ASD dataset we used disease diagnosis: autism spectrum disorder or typically developing (control) as an additional target.

#### 4.3.3 Experiment 3: Quantify the impact of an embedded cell type classifier on batch and cell type separability

Many transcriptomics datasets include cell type labels from an external source. When such labels are available, this information can be leveraged by an *autoencoder classifier* (AEC) to further ensure preservation of cell type information. We construct the scMEDAL-FEC subnetwork, which predicts the one-hot encoded cell type labels $y$ by incorporating a categorical cross-entropy term $\mathcal{L}_{CCE}(y, \hat{y})$ into the fixed effects loss, weighted by $\lambda_y$. The updated fixed effects loss function $\mathcal{L}_{FEC}$ becomes:

$$\mathcal{L}_{FEC}(\boldsymbol{X}, \boldsymbol{Z}, y) = \lambda_{recon,F}\mathcal{L}_{MSE}(\boldsymbol{X}, \widehat{\boldsymbol{X}}) - \lambda_A \mathcal{L}_{CCE}(\boldsymbol{z}, \hat{\boldsymbol{z}}) + \lambda_y \mathcal{L}_{CCE}(y, \hat{y}) \quad (10)$$

We hypothesize that the autoencoder-based cell type classifier will induce a trade-off between the suppression of batch effects and cell type separability. To investigate this, we replace the AE from Experiment 1 with an Autoencoder Classifier (AEC)  designed for cell type classification. with an architecture similar to AutoClass[10] was included as an ablation test for the scMEDAL-FEC model. This facilitated the evaluation of the specific contribution of the classifier to cell type signal preservation and batch correction. In this setup, we evaluate not only how the latent space classifier influences cell type and batch separability, but also whether these effects are primarily driven by the classifier itself or by the adversarial component.

### 4.4 Performance Metrics

The core metric used to guide our scMEDAL training is the validation total loss for each of the subnetworks which is described in equations 2, 8, and 10. Additionally, clustering metrics were used to evaluate the biological separability, batch correction, and batch modeling.

#### 4.4.1 Clustering performance metrics

To evaluate cell type separability, batch correction, and batch modeling capabilities[21,22,24], we utilize three clustering metrics: the Average Silhouette Width (ASW)[20], Calinski-Harabasz (CH) index[23], and Davies-Bouldin (DB) index[25]. The ASW is a widely used metric[13,17,26-28] that quantifies how well each cell is assigned to its cluster compared to other clusters. The DB index quantifies how much clusters overlap (lower values indicate less overlap), while the CH index measures between-cluster *versus* within-cluster variation (higher values suggest better-defined clusters). Unlike the Calinski-Harabasz (CH) index and the Davies-Bouldin (DB) index—which assume convex or spherical clusters and focus on cluster variance structure and overlap, respectively—ASW does not rely on such assumptions.[29] Therefore in this work we choose ASW to serve as our primary metric because it is more robust to clusters of varying shapes compared to CH and DB[29] and its values range from -1 to 1, facilitating comparisons across methods and datasets. We still including the CH and DB indices, thereby ensuring a comprehensive evaluation from different perspectives: CH captures variance structure and compactness, while DB highlights the degree of overlap between clusters and to facilitate comparison to other studies. See Supplementary information section 2.6 for the mathematical formulation of each metric.

#### 4.4.2 Classification performance metrics

To evaluate the classification performance of predicting labels from the scMEDAL latent spaces, we calculated accuracy, balanced accuracy, and chance accuracy. For chance accuracy, we employed scikit-learn's *Dummy Classifier* with the *stratified* strategy, which generates random predictions while maintaining the original class distribution in the dataset.[31] This provides a more realistic baseline for comparison, as it accounts for the imbalanced nature of the classes rather than assuming uniform distribution, thus offering a more appropriate measure of chance-level performance. All these metrics range between 0 and 100%. Balanced accuracy accounts for class imbalance, which is crucial in our datasets due to multiple cell type classes with varying abundances. In the results section, we report results including ASW, classification accuracy, balanced accuracy and chance accuracy on the test data, which is not used for model training nor model selection.

### 4.5 Hyperparameter optimization

There are three types of hyperparameters that shape the proposed framework, including: (1) dimensions of the autoencoder neural networks (e.g., the fixed and random effects subnetworks), (2) choices that govern the optimization of the models' loss function, and (3) the weights that balance the individual terms of the overall loss function. The proposed framework does not require precise tuning of all these design decisions. In fact, reasonable estimates were made for the first two types of hyperparameters, and this sufficed to obtain good performance and convergence properties for all five of the autoencoder models (AE, AEC, scMEDAL-FE, scMEDAL-FEC, scMEDAL-RE) across all three datasets. This underscores the appropriateness of the chosen approach for scRNA-seq analysis. All the autoencoder models, for all datasets, employed three hidden layers with 512 neurons in the first layer, 132 in the second layer, and two neurons in the bottleneck (third) layer. Weight tying between the encoder and decoder components was used for all autoencoders except for scMEDAL-RE, where it was not needed. For the loss function optimization, all models used the Adam optimizer with a fixed learning rate of $lr = 0.0001$. Additionally, for all datasets and models, early stopping based on validation loss was utilized as a convenient and reliable approach for regularization and the prevention of overfitting, ensuring robust and generalizable models. Taken together, this suggests the broad generalizability of the proposed approach and attests to the fairness of the comparison between the proposed approach and the AE and AEC models, as all models employed the same network depth.

In practice we find that only the scaling weights of the terms in the loss function, needs to be optimized per dataset (see Supplementary **Tables** S2–S4 for the values employed) and this optimization is straightforward using the approach we will now describe. We begin by partitioning the data into training, validation and test sets using a 5-fold, multiple hold out, cross-validation strategy. Proper optimization is then achieved by ensuring that the loss terms, such as the reconstruction loss, have roughly the same order of magnitude as the other loss terms during the initial training epochs. Whether the terms are balanced can be readily inspected by constructing training curves with separate curves plotted for the contribution of each loss term, as well as a curve for the total loss function (See Supplementary **Figs**. S6-S11). Weights can then be readily adjusted so that each of the terms (adversarial, cell type classifier, batch classifier, and KL losses) is roughly the same magnitude as the reconstruction loss during the initial epochs of training. Maintaining this balance facilitates proper optimization across all components throughout training. Note that when constructing the AEC and scMEDAL-FEC models, additional information is available in the form of cell type labels. In that scenario it is prudent to additionally monitor cell type separability in the latent spaces as measured by cell type ASW to further optimize weight selection. For model selection, the hyperparameter weights that attained the highest performance on the validation folds are chosen. To minimize inflation bias, the final model's performance is evaluated on the held-out test folds that were not used in training or validation. In general, using the strategy described above, the weights for the loss terms for all models are readily tuned to balance the terms, leading to the rapid convergence of the model weights through total loss function optimization.

### 4.6 Visualization methods

To provide a comprehensive evaluation of our model's performance, two visualization techniques were employed: Uniform Manifold Approximation and Projection (UMAP)[18] and genomaps[36]. The UMAP approach is used to visualize learned latent spaces for an intuitive understanding of the spaces and as a complement to the quantitative evaluation metrics: DB index, CH index, and ASW. This combined approach allows us to better assess both cell-type signal enhancement and batch separability. The genomap approach was used to construct intuitive 2D image representations of each cell's gene expression profile. This approach arranges gene expression levels into a 2D polar coordinate grid according to their gene-to-gene interactions. In this coordinate space, the origin of the image is at the center of the image. Genes are placed with a distance $\rho$ from the image origin (center of image) proportional to their level of interaction with other genes. Thus, genes positioned at the same distance, $\rho$, from the center have equivalent levels of gene-to-gene interaction. The angular component, $\theta$, is arbitrary. The intensity of the pixel in the image representation corresponds to the gene expression level. Hence, if there are many highly expressed genes with the same gene-to-gene interaction, a bright annulus will appear in the image, whose radius corresponds to the common level of interaction. Islam and Xing et al. (2023) demonstrated that genomaps can create gene expression patterns specific to cell types[36]. Importantly, genomap representations preserves the original gene expression values while organizing the genes spatially, allowing for a clear visualization of the relationships between genes and providing insights into batch effects when combined with our generative modeling framework. We use genomap visualization to gain greater understanding of cell-to-cell differences (e.g., malignant vs healthy cells) in the original data and in the batch agnostic representation learned by the fixed effects portion (scMEDAL-FE) of our framework and batch-to-batch variability learned by the random effects portion (scMEDAL-RE) of our framework.

## 5. Code and data availability

Upon acceptance of this publication in a journal, all code for this paper including interpretable scRNA-seq analysis, ablation tests and reproducibility scripts, will be released on GitHub: https://github.com/DeepLearningForPrecisionHealthLab/scMEDAL_for_scRNAseq. Gene expression matrices and cell annotations for the AML dataset were obtained from the Gene Expression Omnibus (GEO) under accession number GSE116256[35]. The Healthy Heart dataset was acquired from Yu et al. (2023) through figshare (DOI: https://doi.org/10.6084/m9.figshare.20499630.v2)[33], and it may also be obtained from the Heart Cell Atlas (https://www.heartcellatlas.org/)[30]. The ASD dataset was obtained from the UCSC Cell Browser (https://autism.cells.ucsc.edu )[34,61]

## 6. Acknowledgements

This study was supported by the NIH R01 Grant "Correcting Biases in Deep Learning Models" (5R01GM144486-02). We also acknowledge the BioHPC supercomputing facility at the Lyda Hill Department of Bioinformatics, UT Southwestern Medical Center, TX, for providing computational resources (URL: https://portal.biohpc.swmed.edu). Special thanks to Dr. Prapti Mody for her invaluable feedback on this work.

## References

1 Bouland, G. A., Mahfouz, A. & Reinders, M. J. T. Consequences and opportunities arising due to sparser single-cell RNA-seq datasets. *Genome Biol.* **24**, 86 (2023).

2 Wu, Y. & Zhang, K. Tools for the analysis of high-dimensional single-cell RNA sequencing data. *Nat. Rev. Nephrol.* **16**, 408-421 (2020).

3 Tung, P. Y., Blischak, J. D., Hsiao, C. J., Knowles, D. A., Burnett, J. E., Pritchard, J. K. & Gilad, Y. Batch effects and the effective design of single-cell gene expression studies. *Sci. Rep.* **7**, 39921 (2017).

4 Haghverdi, L., Lun, A. T. L., Morgan, M. D. & Marioni, J. C. Batch effects in single-cell RNA-sequencing data are corrected by matching mutual nearest neighbors. *Nat. Biotechnol.* **36**, 421-427 (2018).

5 Luecken, M. D. & Theis, F. J. Current best practices in single-cell RNA-seq analysis: a tutorial. *Mol. Syst. Biol.* **15**, e8746 (2019).

6 Hwang, H., Jeon, H., Yeo, N. & Baek, D. Big data and deep learning for RNA biology. *Exp. Mol. Med.* **56**, 1293-1321 (2024).

7 Wang, T., Johnson, T. S., Shao, W., Lu, Z., Helm, B. R., Zhang, J. & Huang, K. BERMUDA: a novel deep transfer learning method for single-cell RNA sequencing batch correction reveals hidden high-resolution cellular subtypes. *Genome Biol.* **20**, 165 (2019).

8 Li, X., Wang, K., Lyu, Y., Pan, H., Zhang, J., Stambolian, D., Susztak, K., Reilly, M. P., Hu, G. & Li, M. Deep learning enables accurate clustering with batch effect removal in single-cell RNA-seq analysis. *Nat. Commun.* **11**, 2338 (2020).

9 Gronbech, C. H., Vording, M. F., Timshel, P. N., Sonderby, C. K., Pers, T. H. & Winther, O. scVAE: variational auto-encoders for single-cell gene expression data. *Bioinformatics* **36**, 4415-4422 (2020).

10 Li, H., Brouwer, C. R. & Luo, W. A universal deep neural network for in-depth cleaning of single-cell RNA-Seq data. *Nat. Commun.* **13**, 1901 (2022).

11 Wang, D., Hou, S., Zhang, L., Wang, X., Liu, B. & Zhang, Z. iMAP: integration of multiple single-cell datasets by adversarial paired transfer networks. *Genome Biol.* **22**, 63 (2021).

12 Wang, Y., Liu, T. & Zhao, H. ResPAN: a powerful batch correction model for scRNA-seq data through residual adversarial networks. *Bioinformatics* **38**, 3942-3949 (2022).

13 Shree, A., Pavan, M. K. & Zafar, H. scDREAMER for atlas-level integration of single-cell datasets using deep generative model paired with adversarial classifier. *Nat. Commun.* **14**, 7781 (2023).

14 Wang, X., Zhang, C., Wang, L. & Zheng, P. Integrating multiple single-cell RNA sequencing datasets using adversarial autoencoders. *Int. J. Mol. Sci.* **24**, 5502 (2023).

15 Danino, R., Nachman, I. & Sharan, R. Batch correction of single-cell sequencing data via an autoencoder architecture. *Bioinform. Adv.* **4**, vbad186 (2024).

16 Ko, K. D. & Sartorelli, V. A deep learning adversarial autoencoder with dynamic batching displays high performance in denoising and ordering scRNA-seq data. *iScience* **27**, 109027 (2024).

17 Luecken, M. D., Buttner, M., Chaichoompu, K., Danese, A., Interlandi, M., Mueller, M. F., Strobl, D. C., Zappia, L., Dugas, M., Colome-Tatche, M. & Theis, F. J. Benchmarking atlas-level data integration in single-cell genomics. *Nat. Methods* **19**, 41-50 (2022).

18 McInnes, L., Healy, J. & Melville, J. UMAP: Uniform Manifold Approximation and Projection for Dimension Reduction. Journal of Open Source Software 3(29) (2018).

19 Breiman, L. Random Forests. *Mach. Learn.* **45**, 5-32 (2001).

20 Rousseeuw, P. J. Silhouettes: A graphical aid to the interpretation and validation of cluster analysis. *J. Comput. Appl. Math.* **20**, 53-65 (1987).

21 Li, X., Zhang, S. & Wong, K. C. Deep embedded clustering with multiple objectives on scRNA-seq data. *Brief. Bioinform.* **22**, bbab090 (2021).

22 Ning, Z., Dai, Z., Zhang, H., Chen, Y. & Yuan, Z. A clustering method for small scRNA-seq data based on subspace and weighted distance. *PeerJ* **11**, e14706 (2023).

23 Calinski, T. & Harabasz, J. A dendrite method for cluster analysis. *Commun. Stat. Theory Methods* **3**, 1-27 (1974).
24 Wang, L., Hong, C., Song, J. & Yao, J. CTEC: a cross-tabulation ensemble clustering approach for single-cell RNA sequencing data analysis. *Bioinformatics* **40**, btae130 (2024).
25 Davies, D. L. & Bouldin, D. W. A Cluster Separation Measure. *IEEE Trans. Pattern Anal. Mach. Intell.* **1**, 224-227 (1979).
26 Yu, L., Cao, Y., Yang, J. Y. H. & Yang, P. Benchmarking clustering algorithms on estimating the number of cell types from single-cell RNA-sequencing data. *Genome Biol.* **23**, 49 (2022).
27 Hu, Y., Xie, M., Li, Y., Rao, M., Shen, W., Luo, C., Qin, H., Baek, J. & Zhou, X. M. Benchmarking clustering, alignment, and integration methods for spatial transcriptomics. *Genome Biol.* **25**, 212 (2024).
28 Yu, X., Xu, X., Zhang, J. & Li, X. Batch alignment of single-cell transcriptomics data using deep metric learning. *Nat. Commun.* **14**, 960 (2023).
29 Vendramin, L., Campello, R. J. G. B. & Hruschka, E. R. Relative clustering validity criteria: A comparative overview. *Stat. Anal. Data Min.* **3**, 209-235 (2010).
30 Litvinukova, M., Talavera-Lopez, C., Maatz, H., Reichart, D., Worth, C. L., Lindberg, E. L., Kanda, M., Polanski, K., Heinig, M., Lee, M., Nadelmann, E. R., Roberts, K., Tuck, L., Fasouli, E. S., DeLaughter, D. M., McDonough, B., Wakimoto, H., Gorham, J. M., Samari, S., Mahbubani, K. T., Saeb-Parsy, K., Patone, G., Boyle, J. J., Zhang, H., Viveiros, A., Oudit, G. Y., Bayraktar, O. A., Seidman, J. G., Seidman, C. E., Noseda, M., Hubner, N. & Teichmann, S. A. Cells of the adult human heart. *Nature* **588**, 466-472 (2020).
31 Pedregosa, F., Eickenberg, M., Ciuciu, P., Thirion, B. & Gramfort, A. Data-driven HRF estimation for encoding and decoding models. *Neuroimage* **104**, 209-220 (2015).
32 Rumelhart, D. E., Hinton, G. E. & Williams, R. J. Learning representations by back-propagating errors. *Nature* **323**, 533-536 (1986).
33 Yu, X., Xu, X., Zhang, J. & Li, X. Batch alignment of single-cell transcriptomics data using deep metric learning. figshare https://doi.org/10.6084/m9.figshare.20499630.v2 (2023).
34 Velmeshev, D., Schirmer, L., Jung, D., Haeussler, M., Perez, Y., Mayer, S., Bhaduri, A., Goyal, N., Rowitch, D. H. & Kriegstein, A. R. Single-cell genomics identifies cell type-specific molecular changes in autism. *Science* **364**, 685-689 (2019).
35 van Galen, P., Hovestadt, V., Wadsworth Ii, M. H., Hughes, T. K., Griffin, G. K., Battaglia, S., Verga, J. A., Stephansky, J., Pastika, T. J., Lombardi Story, J., Pinkus, G. S., Pozdnyakova, O., Galinsky, I., Stone, R. M., Graubert, T. A., Shalek, A. K., Aster, J. C., Lane, A. A. & Bernstein, B. E. Single-Cell RNA-Seq Reveals AML Hierarchies Relevant to Disease Progression and Immunity. *Cell* **176**, 1265-1281.e1224 (2019).
36 Islam, M. T. & Xing, L. Cartography of Genomic Interactions Enables Deep Analysis of Single-Cell Expression Data. *Nat. Commun.* **14**, 679 (2023).
37 Chakrabarti, B., Persico, A., Battista, N. & Maccarrone, M. Endocannabinoid Signaling in Autism. *Neurotherapeutics* **12**, 837-847 (2015).
38 Smith, D. R., Stanley, C. M., Foss, T., Boles, R. G. & McKernan, K. Rare genetic variants in the endocannabinoid system genes CNR1 and DAGLA are associated with neurological phenotypes in humans. *PLoS One* **12**, e0187926 (2017).
39 Stelten, B. M. L., Bonnot, O., Huidekoper, H. H., van Spronsen, F. J., van Hasselt, P. M., Kluijtmans, L. A. J., Wevers, R. A. & Verrips, A. Autism spectrum disorder: an early and frequent feature in cerebrotendinous xanthomatosis. *J. Inherit. Metab. Dis.* **41**, 641-646 (2018).
40 Sicca, F., Ambrosini, E., Marchese, M., Sforna, L., Servettini, I., Valvo, G., Brignone, M. S., Lanciotti, A., Moro, F., Grottesi, A., Catacuzzeno, L., Baldini, S., Hasan, S., D'Adamo, M. C., Franciolini, F., Molinari, P., Santorelli, F. M. & Pessia, M. Gain-of-function defects of astrocytic Kir4.1 channels in children with autism spectrum disorders and epilepsy. *Sci Rep* **6**, 34325 (2016).
41 Du, Y., Li, Z., Liu, Z., Zhang, N., Wang, R., Li, F., Zhang, T., Jiang, Y., Zhi, X., Wang, Z. & Wu, J. Nonrandom occurrence of multiple de novo coding variants in a proband indicates the existence of an oligogenic model in autism. *Genet. Med.* **22**, 170-180 (2020).

42 Lu, L., Guo, H., Peng, Y., Xun, G., Liu, Y., Xiong, Z., Tian, D., Liu, Y., Li, W., Xu, X., Zhao, J., Hu, Z. & Xia, K. Common and rare variants of the THBS1 gene associated with the risk for autism. *Psychiatr. Genet.* **24**, 235-240 (2014).

43 Sun, Y., Yao, X., March, M. E., Meng, X., Li, J., Wei, Z., Sleiman, P. M. A., Hakonarson, H., Xia, Q. & Li, J. Target Genes of Autism Risk Loci in Brain Frontal Cortex. *Front. Genet.* **10**, 707 (2019).

44 Borreca, A., Mantovani, C., Desiato, G., Corradini, I., Filipello, F., Elia, C. A., D'Autilia, F., Santamaria, G., Garlanda, C., Morini, R., Pozzi, D. & Matteoli, M. Loss of interleukin 1 signaling causes impairment of microglia- mediated synapse elimination and autistic-like behaviour in mice. *Brain Behav. Immun.* **117**, 493-509 (2024).

45 Uddin, M. N., Mondal, T., Yao, Y., Manley, K. & Lawrence, D. A. Oxidative stress and neuroimmune proteins in a mouse model of autism. *Cell Stress Chaperones* **28**, 201-217 (2023).

46 Vaccarino, F. M., Grigorenko, E. L., Smith, K. M. & Stevens, H. E. Regulation of cerebral cortical size and neuron number by fibroblast growth factors: implications for autism. *J. Autism Dev. Disord.* **39**, 511-520 (2009).

47 Koren-Michowitz, M., Gery, S., Tabayashi, T., Lin, D., Alvarez, R., Nagler, A. & Koeffler, H. P. SH2B3 (LNK) mutations from myeloproliferative neoplasms patients have mild loss of function against wild type JAK2 and JAK2 V617F. *Br. J. Haematol.* **161**, 811-820 (2013).

48 Perez-Garcia, A., Ambesi-Impiombato, A., Hadler, M., Rigo, I., LeDuc, C. A., Kelly, K., Jalas, C., Paietta, E., Racevskis, J., Rowe, J. M., Tallman, M. S., Paganin, M., Basso, G., Tong, W., Chung, W. K. & Ferrando, A. A. Genetic loss of SH2B3 in acute lymphoblastic leukemia. *Blood* **122**, 2425-2432 (2013).

49 Montoro, M. J., Acha, P., Meggendorfer, M., Al Ali, N. H., Navarro, V., Pierce, S., Bravo-Perez, C., Migeon, M. A., Julia, M., Metzeler, K. H., Wulfert, M., Jerez, A., Díaz-Beyá, M., Betz, B., Clappier, E., Tormo, M., Pomares, H., Del Rey, M., Saumell, S., Barbero, L., Rivero, M. E., Diez-Campelo, M., Sallman, D., Ezponda, T., Chen, W., Xie, Z., Bosch, F., Germing, U., Platzbecker, U., Sole, F., Fenaux, P., Maciejewski, J., Garcia-Manero, G., Komrokji, R. S., Haferlach, T. & Valcarcel, D. The Presence of SH2B3 mutations Identifies a Distinct Subtype of Myelodysplastic Neoplasms. *Blood* **144**, 3214-3214 (2024).

50 Feral, K., Jaud, M., Philippe, C., Di Bella, D., Pyronnet, S., Rouault-Pierre, K., Mazzolini, L. & Touriol, C. ER Stress and Unfolded Protein Response in Leukemia: Friend, Foe, or Both? *Biomolecules* **11** (2021).

51 Zhu, L., Kong, Y., Zhang, J., Claxton, D. F., Ehmann, W. C., Rybka, W. B., Palmisiano, N. D., Wang, M., Jia, B., Bayerl, M., Schell, T. D., Hohl, R. J., Zeng, H. & Zheng, H. Blimp-1 impairs T cell function via upregulation of TIGIT and PD-1 in patients with acute myeloid leukemia. *J. Hematol. Oncol.* **10**, 124 (2017).

52 Upadhyay, P., Beales, J., Shah, N. M., Gruszczynska, A., Miller, C. A., Petti, A. A., Ramakrishnan, S. M., Link, D. C., Ley, T. J. & Welch, J. S. Recurrent transcriptional responses in AML and MDS patients treated with decitabine. *Exp. Hematol.* **111**, 50-65 (2022).

53 Kotmayer, L., Romero-Moya, D., Marin-Bejar, O., Kozyra, E., Catala, A., Bigas, A., Wlodarski, M. W., Bodor, C. & Giorgetti, A. GATA2 deficiency and MDS/AML: Experimental strategies for disease modelling and future therapeutic prospects. *Br J Haematol* **199**, 482-495 (2022).

54 Boyer, T., Gonzales, F., Barthelemy, A., Marceau-Renaut, A., Peyrouze, P., Guihard, S., Lepelley, P., Plesa, A., Nibourel, O., Delattre, C., Wetterwald, M., Pottier, N., Plantier, I., Botton, S., Dombret, H., Berthon, C., Preudhomme, C., Roumier, C. & Cheok, M. Clinical Significance of ABCB1 in Acute Myeloid Leukemia: A Comprehensive Study. *Cancers (Basel)* **11** (2019).

55 Wong, J. C., Bryant, V., Lamprecht, T., Ma, J., Walsh, M., Schwartz, J., Del Pilar Alzamora, M., Mullighan, C. G., Loh, M. L., Ribeiro, R., Downing, J. R., Carroll, W. L., Davis, J., Gold, S., Rogers, P. C., Israels, S., Yanofsky, R., Shannon, K. & Klco, J. M. Germline SAMD9 and SAMD9L mutations are associated with extensive genetic evolution and diverse hematologic outcomes. *JCI Insight* **3** (2018).

56 Monnier, L. & Cournede, P. H. A novel batch-effect correction method for scRNA-seq data based on Adversarial Information Factorization. *PLoS Comput. Biol.* **20**, e1011880 (2024).

57 Tran, H. T. N., Ang, K. S., Chevrier, M., Zhang, X., Lee, N. Y. S., Goh, M. & Chen, J. A benchmark of batch-effect correction methods for single-cell RNA sequencing data. *Genome Biol.* **21**, 12 (2020).
58 Buttner, M., Miao, Z., Wolf, F. A., Teichmann, S. A. & Theis, F. J. A test metric for assessing single-cell RNA-seq batch correction. *Nat. Methods* **16**, 43-49 (2019).
59 Vestal, B. E., Wynn, E. & Moore, C. M. lmerSeq: an R package for analyzing transformed RNA-Seq data with linear mixed effects models. *BMC Bioinformatics* **23**, 489 (2022).
60 Blei, D. M., Kucukelbir, A. & McAuliffe, J. D. Variational Inference: A Review for Statisticians. *J. Am. Stat. Assoc.* **112**, 859-877 (2017).
61 Speir, M. L., Bhaduri, A., Markov, N. S., Moreno, P., Nowakowski, T. J., Papatheodorou, I., Pollen, A. A., Raney, B. J., Seninge, L., Kent, W. J. & Haeussler, M. UCSC Cell Browser: visualize your single-cell data. *Bioinformatics* **37**, 4578-4580 (2021).

## scMEDAL for the interpretable analysis of single-cell transcriptomics data with batch effect visualization using a deep mixed effects autoencoder

Aixa X. Andrade[1], Son Nguyen[1] and Albert Montillo[1]*
[1]Lyda Hill Department of Bioinformatics
University of Texas Southwestern Medical Center
Dallas, TX 75390, USA
*Corresponding author: Albert Montillo[1], Ph.D.; Email: albert.montillo@utsouthwestern.edu

## Supplementary information

### Table of contents

**1. Supplementary materials** .......... **2**

**2. Supplementary methods** .......... **2**

***2.1. Supplementary figures of the scMEDAL architecture*** .......... *2*
**Random effects *Bayesian* layer block** .......... 2

***2.2. Data preprocessing*** .......... *3*

***2.3. Hyperparameters used to build the AE, AEC, scMEDAL-FE, scMEDAL-FEC, and scMEDAL-RE models*** .......... *3*

***2.4. UMAP visualization parameters*** .......... *4*

***2.5. Hardware and software used for model implementation and training*** .......... *4*

***2.6. Clustering metric definitions*** .......... *4*

**3. Supplementary results** .......... **6**

***3.1. Confounding within the AML dataset*** .......... *6*

***3.2. Hyperparameter Optimization (HPO) for AEC and scMEDAL-FEC models*** .......... *7*

***3.3. Supplementary results for scMEDAL-FE and scMEDAL-RE models*** .......... *7*
**3.3.1. CH and 1/DB scores** .......... 7
**3.3.2. Random Effects subnetwork (scMEDAL-RE) latent spaces** .......... 8
**3.3.3. Training and validation curves** .......... 10

***3.4. Supplementary results for scMEDAL-FEC and AEC models*** .......... *13*
**3.4.1. CH and 1/DB scores** .......... 13
**3.4.2. Average training and validation curves for AEC and scMEDAL-FE across 5 folds in the Healthy Heart, ASD, and AML datasets** .......... 14

***3.5. Significant genes from the genomaps for AML versus controls and ASD versus controls*** .......... *17*

**4. References** .......... **19**

## 1. Supplementary materials

**Table** S1 shows the details of the datasets used to evaluate scMEDAL subnetworks as well as the baseline PCA and the AE and AEC models. Data processing steps are described in supplemental section 2.2

**Table S1**. Datasets used to evaluate scMEDAL models.

| | *Healthy Heart*[1,2] | *ASD*[3] | *AML*[4] |
|---|---|---|---|
| Description | Heart cells from multiple tissues | ASD and TD (controls) | AML and healthy subjects |
| Data type | Single cell | Single nucleus | Single cell |
| Total cells (pre-filtering) | 486,134 | 104, 559 | 41,090 |
| Total cells (post-filtering) | 486,134 | 104, 559 | 38,417 |
| Total genes | 33,538 | 36,501 | 27,899 |
| Highly variable genes | 3,000 | 2,916 | 2,916 |
| Number of cell types | 12 + Not Assigned cell type | 17 | 6 malignant + 15 healthy |
| Batch effect type | Technical batch (z =147) | Biological donor (z = 31) | Technical batch (z=19) |

## 2. Supplementary methods

### 2.1. Supplementary figures of the scMEDAL architecture

#### Random effects *Bayesian* layer block

**Fig.** S1 depicts the architecture of the Random Effects Dense (REDEN) Bayesian layer block, which is used to construct the Bayesian layers of the scMEDAL-RE subnetwork.

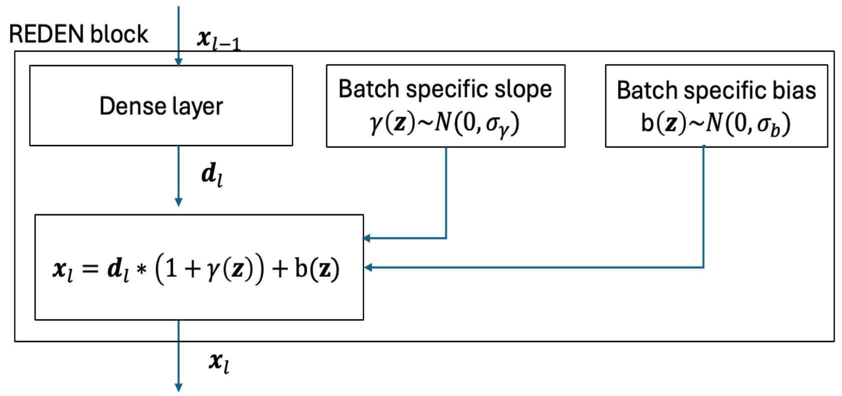

**Fig. S1:** Random effects dense (REDEN) block used in the random effects autoencoder (scMEDAL-RE). A dense layer with $n_l$ neurons transforms an input representation from the preceding layer, $x_{l-1}$, into an intermediate output tensor, $d_l$. A batch-specific scale *γ(**z**)* and bias *b(**z**)* are then applied to each of the *f* feature maps. These batch-specific slopes and biases are regularized to follow normal distributions, with mean zero, and learned variances $\sigma_\gamma$ and $\sigma_b$, respectively.

### 2.2. Data preprocessing

We used standard preprocessing steps from Yu et al. , 2023[5] which included: 1) filtering out cells exhibiting expression in fewer than ten genes and removal of genes detected in fewer than three cells, 2) normalizing total Unique Molecular Identifiers (UMI) counts per cell to 10,000, 3) log-transforming the data to stabilize variance $\log(X+1)$, and 4) selecting the top highly variable genes (HVGs).

Since the ASD and Healthy Heart datasets had previously undergone quality control, no additional cells were removed during the filtering step. For the AML dataset, additional quality control measures were implemented. Cells with undefined or missing cell type annotations were excluded. Furthermore, samples identified by Dai et al., (2021) as having ambiguous annotations—specifically AML314, AML371, AML722B, and AML997[6]—were removed from the original 16 AML donors to ensure the dataset's integrity, resulting in 12 AML donors available for analysis. Including 5 healthy donors and 2 cell lines, the dataset comprised a total of 19 batches. The ASD dataset had previously undergone a log2 transformation as part of the original preprocessing by van Galen et al. (2019). To maintain consistency across all datasets, this transformation was reversed by applying an exponential function (base 2) to the data after adding 1 to each value, thereby returning the data to its original scale. Following this reversal, the standard preprocessing pipeline was applied.

The datasets were then partitioned through 5 fold cross-validation, stratified by batch and cell type in all three datasets. For each split, three folds were used for training, one for validation, and one for testing. The data were loaded for each subset and scaled using min-max scaling before model fitting. To reduce compute time for the ASW calculations, we used a random sample of 10,000 cells when datasets exceeded this size in training, validation, or testing subsets.

### 2.3. Hyperparameters used to build the AE, AEC, scMEDAL-FE, scMEDAL-FEC, and scMEDAL-RE models

Section 4.5 of the main paper describes the hyperparameter optimization process for the overall objective function in equation 9. In the following **Tables** S2-S4, we describe the values of the hyperparameter that are found to optimize the AE, AEC, scMEDAL-FE, scMEDAL-FEC, and scMEDAL-RE models in three datasets: Healthy Heart, the Autism Spectrum Disorder (ASD), and Acute Myeloid Leukemia (AML).

**Table S2.** Hyperparameters used for training PCA, AE and scMEDAL subnetworks in the **Healthy Heart dataset**.

| Hyperparameter | AE | AEC | scMEDAL-FE | scMEDAL-FEC | scMEDAL-RE |
|---|---|---|---|---|---|
| $\lambda_{recon,F}$ | 1 | 81 | 5400 | 9450 | - |
| $\lambda_{recon,R}$ | - | - | - | - | 110 |
| $\lambda_y$ | - | 0.1 | - | 1 | - |
| $\lambda_A$ | - | - | 1 | 1 | - |
| $\lambda_L$ | - | | | | 0.1 |
| $\lambda_K$ | - | | | | 1.00E-05 |

**Table S3.** Hyperparameters used for training PCA, AE and scMEDAL subnetworks in the **ASD dataset**.

| Hyperparameter | AE | AEC | scMEDAL-FE | scMEDAL-FEC | scMEDAL-RE |
|---|---|---|---|---|---|
| $\lambda_{recon,F}$ | 1 | 1 | 1000 | 1000 | - |
| $\lambda_{recon,R}$ | - | - | - | - | 110 |
| $\lambda_y$ | - | 0.1 | - | 1 | - |
| $\lambda_A$ | - | | 1 | 1 | - |
| $\lambda_L$ | - | | | | 0.1 |
| $\lambda_K$ | - | | | | 1.00E-05 |

| Hyperparameter | AE | AEC | scMEDAL-FE | scMEDAL-FEC | scMEDAL-RE |
|---|---|---|---|---|---|
| $\lambda_{recon,F}$ | 1 | 100 | 4000 | 1500 | - |
| $\lambda_{recon,R}$ | - | - | - | - | 110 |
| $\lambda_{y}$ | - | 0.1 | - | 1 | - |
| $\lambda_{A}$ | - | - | 1 | 1 | - |
| $\lambda_{L}$ | - | | | | 0.1 |
| $\lambda_{K}$ | - | | | | 1.00E-05 |

**Table S4.** Hyperparameters used for training PCA, AE and scMEDAL subnetworks **AML dataset**.

### 2.4. UMAP visualization parameters

**Table** S5 describes the minimum distance and nearest neighbor's choices for UMAP visualizations presented in the main paper in sections 2.2, 2.3, 2.4, and 2.7.

**Table S5.** UMAP Visualization parameters.

| Dataset | UMAP parameters | AE | AEC | scMEDAL-FE | scMEDAL-FE | scMEDAL-RE |
|---|---|---|---|---|---|---|
| Healthy Heart | Min distance | 0.5 | | | | 0 |
| | Nearest Neighbors | 15 | | | | 1000 |
| ASD | Min distance | 0.5 | | | | |
| | Nearest Neighbors | 15 | | | | |
| AML | Min distance | 0.5 | | | | |
| | Nearest Neighbors | 15 | | | | |

### 2.5. Hardware and software used for model implementation and training

All deep learning models were developed and trained on Nvidia Tesla V100 GPUs with 32 GB of memory and Tesla P4 GPUs with 8 GB of memory. The software stack for model training included Python 3.8.5, TensorFlow 2.3[7], TensorFlow Probability 0.11.1[7-9], scikit-learn 0.23.2, and Scanpy 1.6.0. Performance measures, including ASW, DB, CH, accuracy, balanced accuracy, and chance accuracy, were computed using the Scikit-learn library[10]. The PCA model was also implemented through the Scikit-learn library. ScRNA-seq preprocessing and UMAP computations[11] were performed using Python 3.8.18 with the Scanpy 1.9.8 library[12]. Genomaps were generated using genomap 1.3.6 and the Mann-Whitney U test was implemented using the using the *stats* module from SciPy 1.10.1[13] and the linear mixed-effects models were implemented in the Statsmodels 0.10.0 library[14]. For more details on the software used for model training, plot creation, and generation of visualizations, please refer to the code repository described in the *Code and data availability* section 5.

### 2.6. Clustering metric definitions

We use the following metrics to quantify the clustering (separability) of the cells: the Average Silhouette Width (ASW)[15], Calinski-Harabasz (CH) index[16], and Davies-Bouldin (DB) index[17]. The *Silhouette Width* quantifies how well each cell fits within its assigned cluster compared to the other clusters. It is computed using two distances: the within-cluster distance ($a_i$) and the between-cluster distance ($b_i$). The within-cluster distance, $a_i$, represents the average distance between a cell $i$ and all other cells in its cluster. This distance is defined as:

$$a_i = \frac{1}{|C_i| - 1} \sum_{\{j \in C_i, j \neq i\}} d(i,j)$$

where $C_i$ is the set of cells in the cluster of cell $i$, $|C_i|$ is the total number of cells in this cluster, and $d(i,j)$ is the distance between cell $i$ and cell $j$ within the cluster. In contrast, the between-cluster distance, $b_i$, is the minimum of the average distance between cell $i$ and cells from other clusters. It is defined as:

$$b_i = min_{\{k \neq i\}} \frac{1}{|C_k|} \sum_{\{j \in C_k\}} d(i,j)$$

where $C_k$ is the set of cells assigned to cluster $k$ which is different from the cluster of cell $i$, while $|C_k|$ is the total number of cells in cluster $k$. The silhouette coefficient for each cell $i$ is calculated as:

$$s_i = \frac{b_i - a_i}{max(b_i, a_i)}$$

where a higher $s_i$ value (closer to 1.0) indicates that the cell is well-clustered, being more similar to cells within its own cluster than to those in any other cluster. The *ASW* is defined as the average of $s_i$ over all cells and provides an overall measure of clustering quality.

The *Davies–Bouldin* (DB) *index* quantifies the extent to which clusters overlap. For each cluster, we compare its average within-cluster dispersion to the distance between its centroid and the centroid of the most similar (closest) cluster. Consequently, a lower DB index value indicates less overlap and better separation between clusters. The DB index is computed as:

$$DB = \left(\frac{1}{K}\right) \sum_{\{k=1\}}^{K} D_k$$

where $K$ is the number of clusters, and $D_k$ for cluster $k$ is the ratio, $R_{kl}$ for the most similar cluster $l$ to $k$, $(l \neq k)$. $D_k$ is expressed as:

$$D_k = max_{\{l \neq k\}} R_{kl}$$

where the ratio $R_{kl}$ measures how similar are the clusters $k$ and $l$. $R_{kl}$ is computed as:

$$R_{kl} = \frac{(q_k + q_l)}{M_{kl}}$$

where $q_k$ is the is the average distance from each cell in cluster $k$ to the centroid of cluster $k$, $q_l$ is the corresponding average distance for cluster $l$, and $M_{kl}$ is the distance between the centroids of clusters $k$ and $l$. A larger $M_{kl}$ indicates better separation between the two clusters.

The *Calinski-Harabasz* (CH) index measures the ratio of between-cluster variance to within-cluster variance, with higher values indicating more distinct and cohesive clusters. It is calculated as

$$CH = \frac{\sum_{\{k=1\}}^{K} n_k * || \mu_k - \mu ||^2}{\sum_{\{k=1\}}^{K} \sum_{\{i=1\}}^{n_k} || p_i - \mu_k ||^2} * \left[\frac{N - K}{K - 1}\right]$$

where $n_k$ is the number of points in the $k^{th}$ cluster, $\mu_k$ is the centroid of the $k^{th}$ cluster, $\mu$ is the centroid of the entire data (all the cells), $p_i$ is the position (coordinates) of the cell $i$, $N$ is the total number of cells and $K$ is the number of clusters.

To align the interpretation of the DB index with the CH index and ASW, we used the reciprocal (1/DB), ensuring that higher scores consistently indicate better clustering quality. When measuring the clustering by cell type, higher scores signify improved separability in the latent space, enhancing the cell type signal—an objective of batch correction. Conversely, when measuring the clustering by batch label, lower scores indicate more effective batch correction, as it indicates that the batches become less distinguishable, which is another objective of batch correction. Additionally, higher batch scores in the batch-specific latent space of the random effects subnetwork indicates effective modeling of the batch effects and capture of the between-batch variance in gene expression data.

## 3. Supplementary results

### 3.1. Confounding within the AML dataset

In **Fig**. S2 the representation of donors across cell types in AML is shown graphically. We observe that not all donor samples contain cells from every cell type, making this dataset a particularly challenging and interesting one for batch correction due to potential confounding between donor identity and cell type.

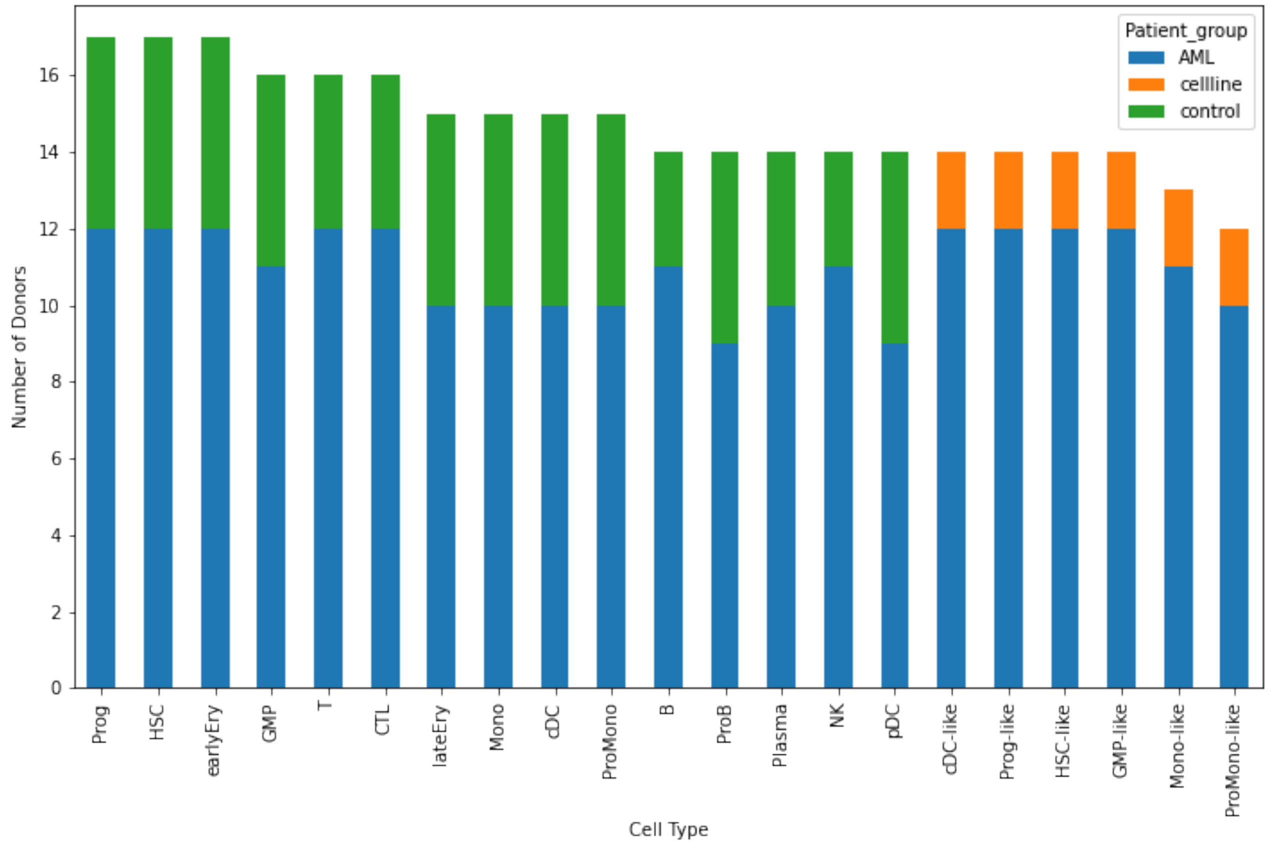

**Fig. S2: AML dataset demonstrates intrinsec cell type-donor confounding.** This stacked bar plot shows the number of donors for each cell type (y-axis), grouped by patient group, with cell types displayed along the x-axis. The colors indicate the donor patient group.

### 3.2. Hyperparameter Optimization (HPO) for AEC and scMEDAL-FEC models

Hyperparameter optimization (HPO) was conducted per dataset to tune the parameters using the held-out validation data (not used for model training). This tuning adjusts the weights of the reconstruction, adversarial, classification (cell type for scMEDAL-FE and batch for scMEDAL-RE), and Kullback–Leibler divergence loss function terms so that they are on a similar scale, allowing all terms to guide the model fitting, and optimizing performance on the validation data. The winning parameters are shown in **Tables** S2-S4. However, when cell type information is available from an external source (as described in Section 2.7 of the main paper) this can be used to further guide hyperparameter tuning. In this scenario, the ASW for cell type can be used to further refine the loss function weights. Both batch and cell-type ASW on the AEC and scMEDAL-FEC models can be evaluated during the training for 500 epochs with early stopping, prioritizing the cell-type ASW. **Table** S6 illustrates how the ASW scores for both batch and cell type separability can vary somewhat for different values of the weights assigned to the reconstruction loss (measured with mean squared error), adversarial loss, and classification loss of the cell type labels $y$, while the selected (winning) parameters are in boldface.

**Table S6.** Average Silhouette Width (ASW) scores for batch and cell type separability of the AML dataset (mean across 5 folds). Hyperparameter Optimization (HPO) selection of reconstruction loss $\lambda_{recon}$ and classification loss $\lambda_y$ weights using validation data. Adversarial loss weight $\lambda_A = 1$ for all models.

| | $\lambda_{recon}$ | $\lambda_y$ | ASW (batch) | | | ASW (cell type) | | |
|---|---|---|---|---|---|---|---|---|
| | | | mean | 95% CI | | mean | 95% CI | |
| AEC | **1** | **0.1** | -0.43 | -0.52 | -0.34 | -0.15 | -0.23 | -0.06 |
| | 10 | | -0.37 | -0.44 | -0.31 | -0.11 | -0.20 | -0.02 |
| | **100** | | **-0.28** | **-0.34** | **-0.23** | **0.03** | **-0.04** | **0.10** |
| scMEDAL-FEC | 500 | 1 | -0.33 | -0.35 | -0.31 | -0.10 | -0.13 | -0.06 |
| | **1500** | **1** | **-0.30** | **-0.37** | **-0.24** | **-0.05** | **-0.08** | **-0.02** |
| | 1500 | 2 | -0.33 | -0.40 | -0.26 | -0.07 | -0.11 | -0.04 |
| | 2000 | 2 | -0.31 | -0.33 | -0.28 | -0.07 | -0.13 | -0.01 |

### 3.3. Supplementary results for scMEDAL-FE and scMEDAL-RE models

#### 3.3.1. CH and 1/DB scores

In **Tables** S7-S9, scores are shown from the Calinski-Harabasz (CH) clustering index and the reciprocal of the Davies-Bouldin (DB) clustering index (1/DB), as these complement the primary metric, ASW, used in the main paper. These scores confirm the ASW results. In particular, the scMEDAL-RE subnetwork consistently captures batch specific information as designed, demonstrating significantly more batch information (higher scores) than any of the other models, as measured with both the CH and 1/DB indices, across all three datasets (4th row, first 2 columns of **Tables** S7-S9). Meanwhile, the scMEDAL-FE subnetwork substantially suppresses batch specific information, demonstrating much lower CH scores (less batch contamination) than the baseline model (PCA) in all three datasets (3rd row, 2nd column of **Tables** S7-S9). In terms of preserving cell type information, across all datasets, the scMEDAL-FE subnetwork outperforms the baseline model (PCA) and scMEDAL-RE (by design) with higher scores in the 1/DB metric (rows 1,3,4 of 3rd column) and performs comparably well to the AE model in the CH metric (rows 2,3 of 4th column of **Tables** S7-S9).

**Table S7.** 1/DB and CH scores (Mean and 95% CI across 5 folds) for batch and cell type separability in the latent spaces of the **Healthy Heart dataset**, using PCA, AE, scMEDAL-FE, and scMEDAL-RE models.

| | batch | | | | | | cell type | | | | | |
|---|---|---|---|---|---|---|---|---|---|---|---|---|
| | 1/DB | | | CH | | | 1/DB | | | CH | | |
| | mean | 95% CI | | mean | 95% CI | | mean | 95% CI | | mean | 95% CI | |
| PCA (Baseline) | 0.02 | 0.01 | 0.03 | 148.65 | 145.33 | 151.98 | 0.13 | 0.10 | 0.15 | 1502.74 | 1456.79 | 1548.69 |
| AE | 0.02 | 0.02 | 0.02 | 70.49 | 51.09 | 89.90 | 0.22 | 0.10 | 0.33 | 1712.76 | 1405.70 | 2019.83 |
| scMEDAL-FE | 0.02 | 0.01 | 0.02 | 61.38 | 38.74 | 84.03 | 0.19 | 0.13 | 0.24 | 1984.01 | 1078.08 | 2889.95 |
| scMEDAL-RE | 0.56 | 0.17 | 0.94 | 2555.27 | 544.02 | 4566.51 | 0.06 | 0.04 | 0.08 | 224.76 | 66.19 | 383.34 |

**Table S8.** 1/DB and CH scores (Mean and 95% CI across 5 folds) for batch and cell type separability in the latent spaces of the **ASD dataset**, using PCA, AE, scMEDAL-FE, and scMEDAL-RE models.

| | batch | | | | | | cell type | | | | | |
|---|---|---|---|---|---|---|---|---|---|---|---|---|
| | 1/DB | | | CH | | | 1/DB | | | CH | | |
| | mean | 95% CI | | mean | 95% CI | | mean | 95% CI | | mean | 95% CI | |
| PCA (Baseline) | 0.03 | 0.02 | 0.03 | 32.67 | 32.06 | 33.28 | 0.18 | 0.15 | 0.21 | 9089.94 | 8906.86 | 9273.02 |
| AE | 0.02 | 0.02 | 0.03 | 23.72 | 20.74 | 26.69 | 0.78 | 0.71 | 0.85 | 9863.14 | 8673.06 | 11053.22 |
| scMEDAL-FE | 0.02 | 0.02 | 0.03 | 19.43 | 14.12 | 24.74 | 0.32 | 0.15 | 0.49 | 9631.75 | 8052.25 | 11211.25 |
| scMEDAL-RE | 0.16 | -0.09 | 0.40 | 2423.99 | -3510.80 | 8358.77 | 0.03 | 0.01 | 0.04 | 749.41 | -261.64 | 1760.45 |

**Table S9.** 1/DB and CH scores (Mean and 95% CI across 5 folds) for batch and cell type separability in the latent spaces of the **AML dataset**, using PCA, AE, scMEDAL-FE, and scMEDAL-RE models.

| | batch | | | | | | cell type | | | | | |
|---|---|---|---|---|---|---|---|---|---|---|---|---|
| | 1/DB | | | CH | | | 1/DB | | | CH | | |
| | mean | 95% CI | | mean | 95% CI | | mean | 95% CI | | mean | 95% CI | |
| PCA (Baseline) | 0.06 | 0.05 | 0.07 | 156.52 | 154.20 | 158.84 | 0.14 | 0.12 | 0.16 | 2560.96 | 2482.14 | 2639.79 |
| AE | 0.07 | 0.07 | 0.08 | 189.78 | 169.23 | 210.32 | 0.28 | 0.24 | 0.32 | 1956.48 | 1779.39 | 2133.58 |
| scMEDAL-FE | 0.07 | 0.06 | 0.09 | 80.63 | 69.88 | 91.37 | 0.19 | 0.09 | 0.29 | 1493.43 | 1353.85 | 1633.01 |
| scMEDAL-RE | 0.37 | 0.07 | 0.67 | 63228.21 | 9245.17 | 117211.25 | 0.07 | 0.05 | 0.09 | 114.73 | 69.53 | 159.94 |

#### 3.3.2. Random Effects subnetwork (scMEDAL-RE) latent spaces

**Figs.** S3–S5 show the scMEDAL-RE latent spaces through UMAP visualizations. We observe that the batches (colored) are visibly apparent across all datasets. This further confirms the high degree of batch separability attainable by the scMEDAL-RE subnetwork, which is as designed, because its purpose is to model the batch effects.

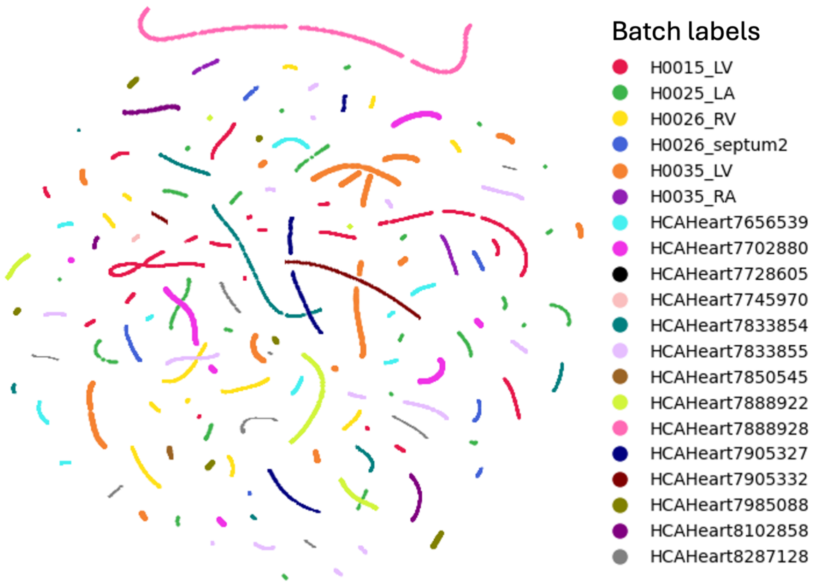

**Fig. S3: scMEDAL-RE separates cells from different batches in the Healthy Heart dataset.** UMAP visualization of scMEDAL-RE latent components, representing 44,987 cells from 20 selected batches (out of 147 total). Colors indicate different batches, demonstrating the separation of cells by batch in the scMEDAL-RE latent space.

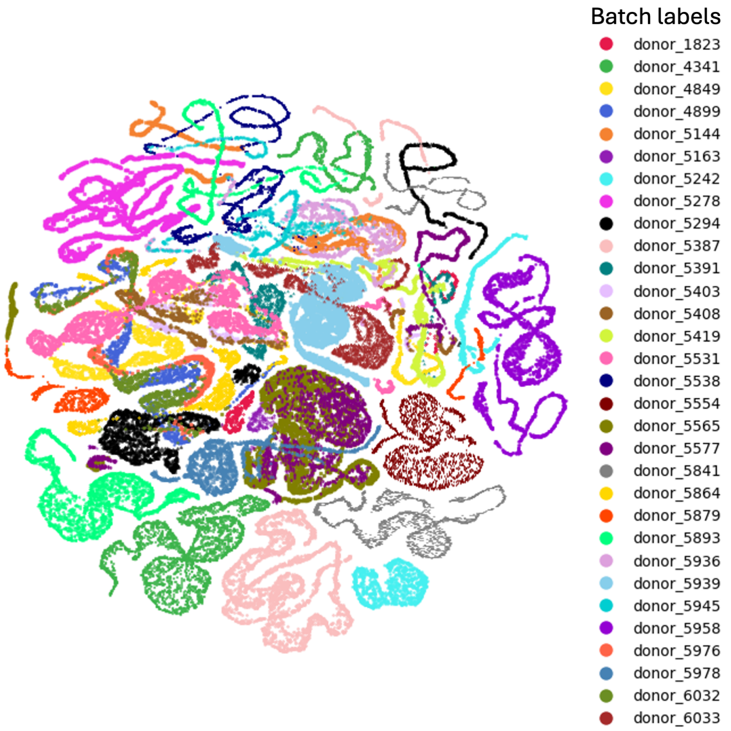

**Fig. S4: scMEDAL-RE separates cells from different donors in the ASD dataset.** UMAP visualization of scMEDAL-RE latent components, representing 62,735 cells from 31 donors. Colors indicate different donors, demonstrating the separation of cells by donors in the scMEDAL-RE latent space.

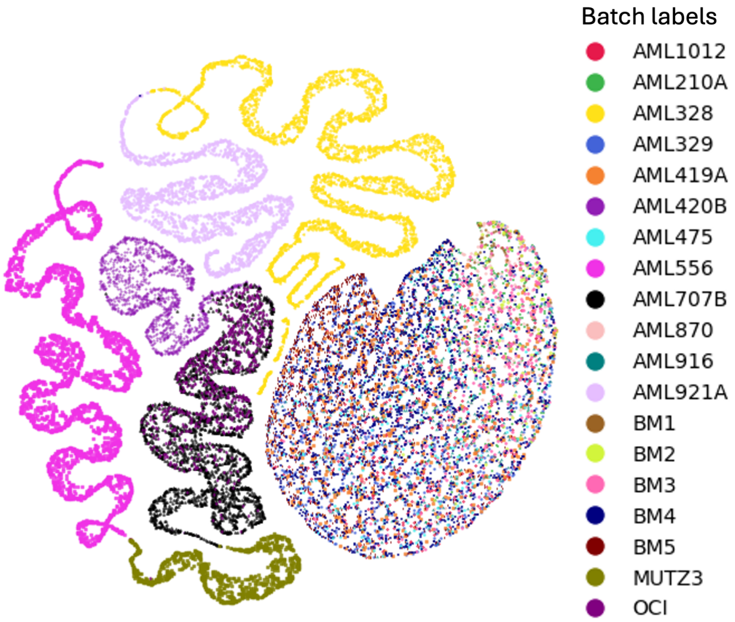

**Fig. S5: scMEDAL-RE separates cells from cellines, AML and healthy donors in the AML dataset.** Latent spaces from 23,050 cells from the AML dataset latent spaces obtained with scMEDAL-RE. UMAP applied to the scMEDAL-RE latent space.

#### 3.3.3. Training and validation curves

**Figs.** S6-S8 present the training and validation curves, for the AE, scMEDAL-FE, and scMEDAL-RE models in all three datasets. For scMEDAL-FE, the total loss is calculated as the reconstruction loss minus the adversarial loss, with each component scaled by its respective weight. In the scMEDAL-RE curves, the left axis displays the reconstruction loss and total loss, while the right axis shows: the latent cluster loss classifying a cell's batch and the Kullback-Leibler divergence (KLD) loss. Also shown is the total loss, which is computed as the sum of the reconstruction loss, latent cluster loss, and KLD loss, each adjusted by their respective weights. We observe that through all models, no one term dominates. Instead the individual terms have values in similar ranges and can contribute effectively to the overall loss, thereby guiding the model fitting.

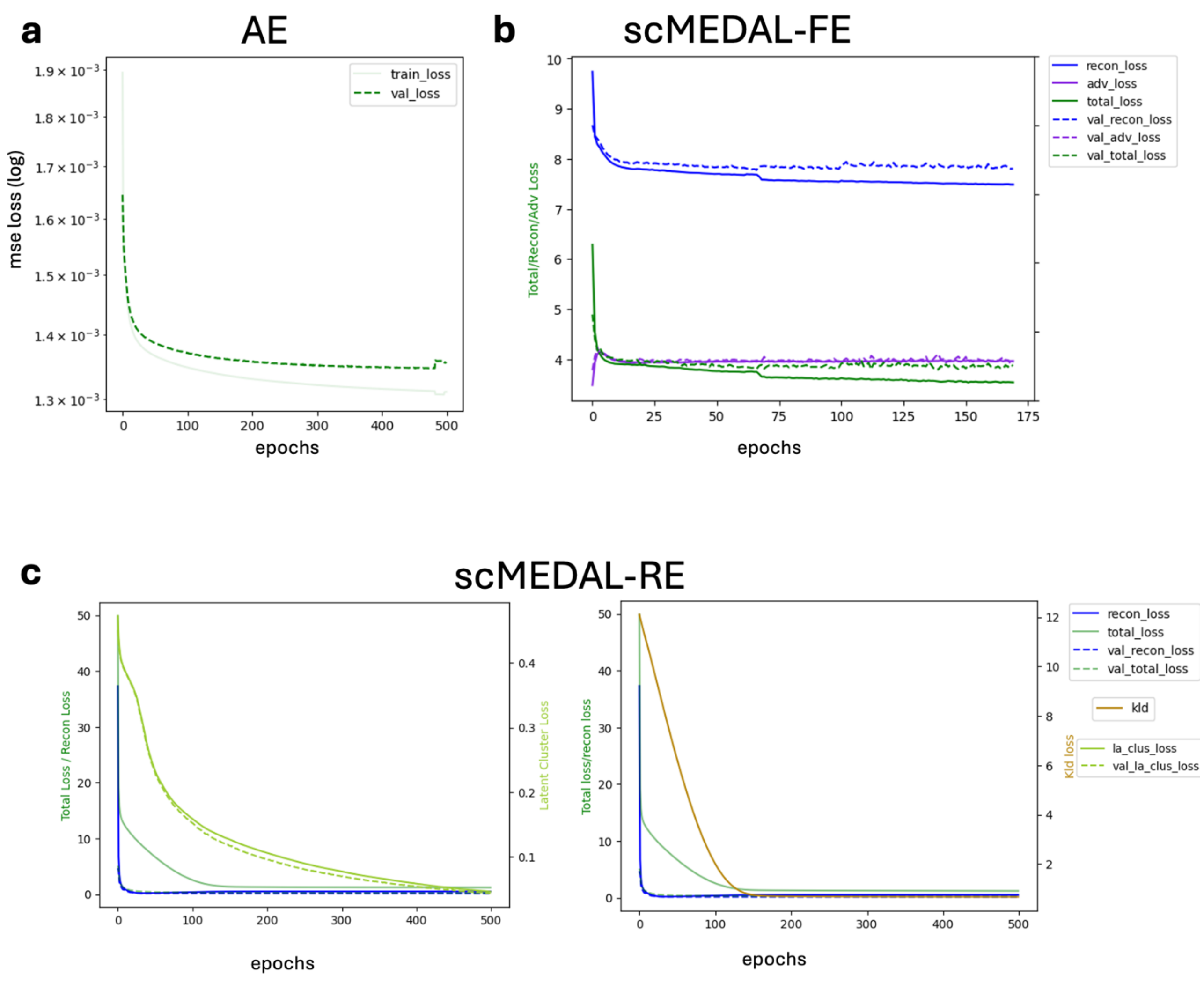

**Fig. S6: Training and validation curves for the Healthy Heart dataset.** Individual loss terms (see legend) maintain a similar scale throughout training. Training and validation curves adjusted by weights for **a** AE, **b** scMEDAL-FE (total loss = reconstruction loss - adversarial loss), and **c** scMEDAL-RE (total loss = reconstruction loss + KLD loss + latent cluster loss).

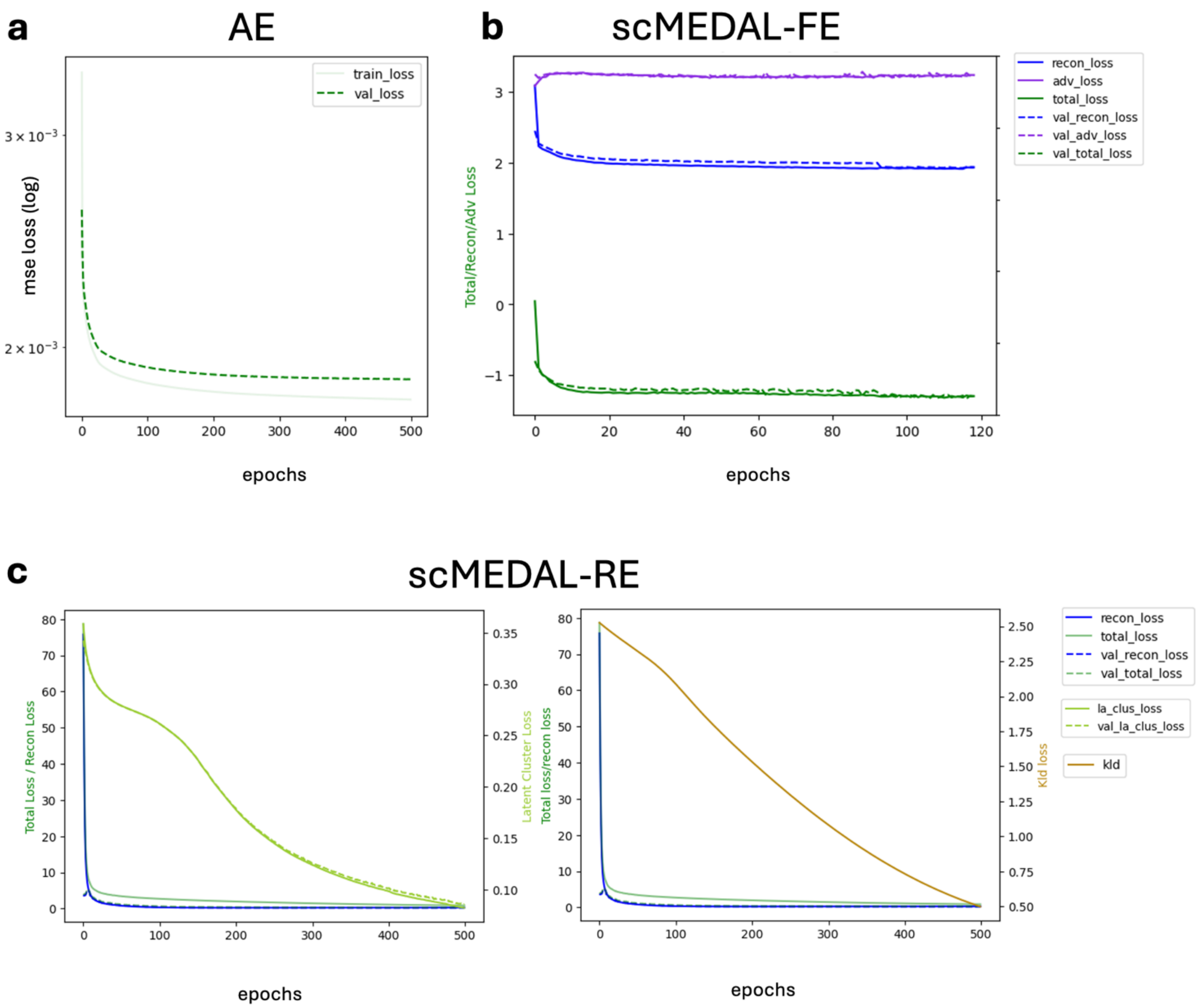

**Fig. S7: Training and validation curves for the ASD dataset.** Individual loss terms (see legend) maintain a similar scale throughout training. Training and validation curves adjusted by weights for **a** AE, **b** scMEDAL-FE (total loss = reconstruction loss - adversarial loss), and **c** scMEDAL-RE (total loss = reconstruction loss + KLD loss + latent cluster loss)

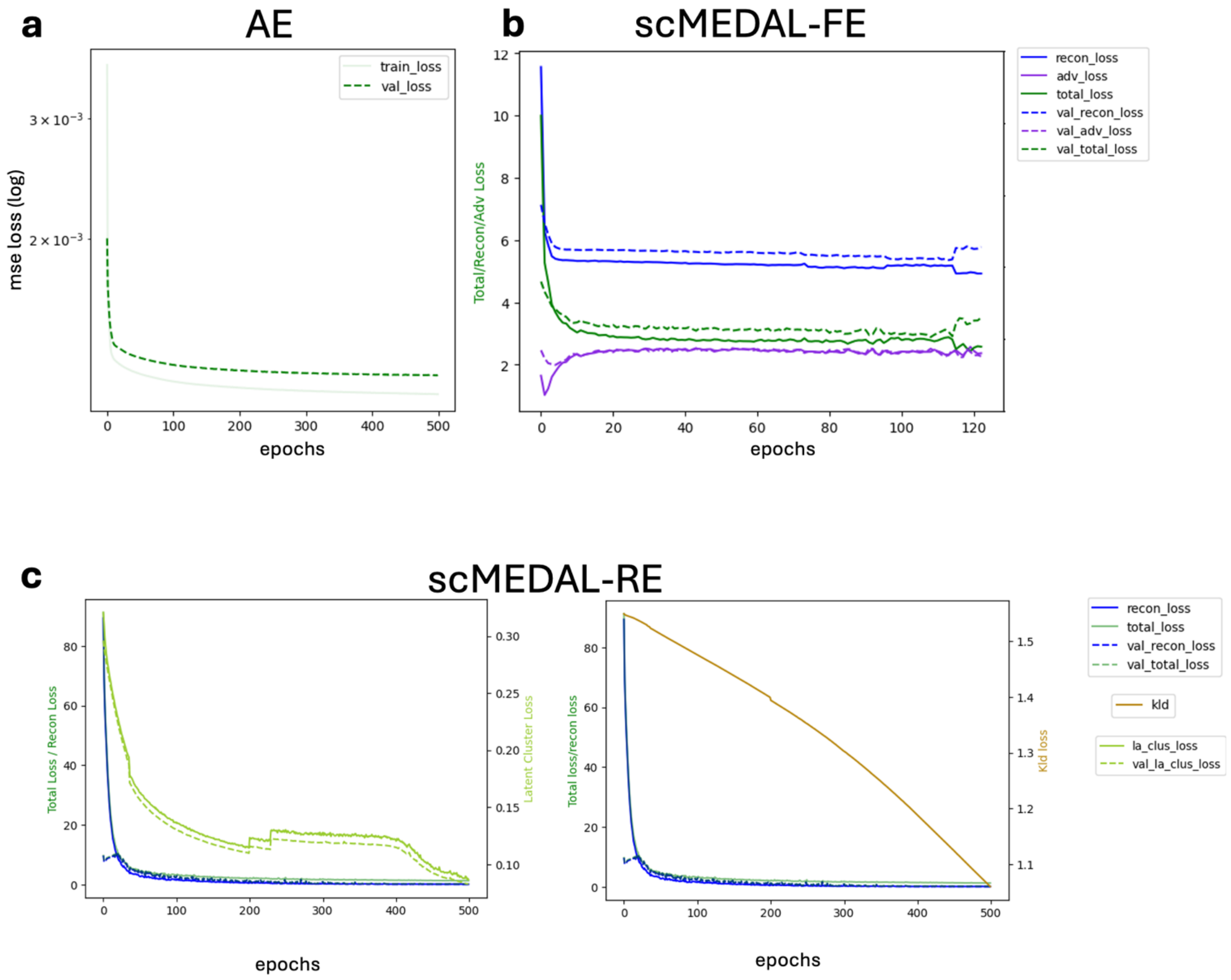

**Fig. S8: Training and validation curves for the AML dataset.** Individual loss terms (see legend) maintain a similar scale throughout training. Training and validation curves adjusted by weights for **a** AE, **b** scMEDAL-FE (total loss = reconstruction loss - adversarial loss), and **c** scMEDAL-RE (total loss = reconstruction loss + KLD loss + latent cluster loss)

### 3.4. Supplementary results for scMEDAL-FEC and AEC models

#### 3.4.1. CH and 1/DB scores

In **Tables** S10-S12 scores that complement the primary metric, ASW, are shown, including the Calinski-Harabasz (CH) clustering index and the reciprocal of the Davies-Bouldin (DB) clustering index (1/DB). These results further corroborate the ASW results in Section 2.7 of the main paper. The scMEDAL-FEC subnetwork substantially suppresses batch specific information, demonstrating lower CH scores (less batch contamination) than the AEC in all three datasets (rows 1,2 of the 2nd column of **Tables** S10-S12). In terms of preserving cell type information, both models perform admirably and similarly, including in their CH scores for the ASD and AML datasets (4th column of **Tables** S11-S12) and the 1/DB score for the Healthy Heart dataset (3rd column of **Table** S10). As described in the main paper (Section 2.7) the UMAP latent space visualizations provide a more nuanced view including additional comparative performance benefits of scMEDAL-FEC over the AEC model across datasets.

**Table S10.** 1/DB and CH scores (Mean and 95% CI across 5 folds) for batch and cell type separability in the latent spaces of the **Healthy Heart dataset**, using AEC and scMEDAL-FEC models.

| | batch | | | | | | cell type | | | | | |
|---|---|---|---|---|---|---|---|---|---|---|---|---|
| | 1/DB | | | CH | | | 1/DB | | | CH | | |
| | mean | 95% CI | | mean | 95% CI | | mean | 95% CI | | mean | 95% CI | |
| AEC | 0.02 | 0.01 | 0.03 | 48.29 | 15.26 | 81.32 | 0.23 | 0.06 | 0.41 | 5563.96 | 2833.93 | 8293.99 |
| scMEDAL-FEC | 0.02 | 0.01 | 0.02 | 41.39 | 27.01 | 55.77 | 0.21 | 0.04 | 0.38 | 3750.68 | 2318.16 | 5183.19 |

**Table S11.** 1/DB and CH scores (Mean and 95% CI across 5 folds) for batch and cell type separability in the latent spaces of the **ASD dataset**, using AEC and scMEDAL-FEC models.

| | batch | | | | | | cell type | | | | | |
|---|---|---|---|---|---|---|---|---|---|---|---|---|
| | 1/DB | | | CH | | | 1/DB | | | CH | | |
| | mean | 95% CI | | mean | 95% CI | | mean | 95% CI | | mean | 95% CI | |
| AEC | 0.02 | 0.01 | 0.03 | 20.06 | 14.48 | 25.64 | 0.73 | 0.51 | 0.95 | 10631.01 | 5846.41 | 15415.61 |
| scMEDAL-FEC | 0.02 | 0.01 | 0.02 | 12.72 | 7.40 | 18.03 | 0.34 | 0.18 | 0.50 | 9911.04 | 8726.06 | 11096.02 |

**Table S12.** 1/DB and CH scores (Mean and 95% CI across 5 folds) for batch and cell type separability in the latent spaces of the **AML dataset**, using AEC and scMEDAL-FEC models.

| | batch | | | | | | cell type | | | | | |
|---|---|---|---|---|---|---|---|---|---|---|---|---|
| | 1/DB | | | CH | | | 1/DB | | | CH | | |
| | mean | 95% CI | | mean | 95% CI | | mean | 95% CI | | mean | 95% CI | |
| AEC | 0.08 | 0.04 | 0.12 | 142.75 | 81.95 | 203.54 | 0.17 | 0.09 | 0.25 | 2159.15 | 1216.68 | 3101.61 |
| scMEDAL-FEC | 0.06 | 0.04 | 0.07 | 87.24 | 70.03 | 104.45 | 0.18 | 0.10 | 0.25 | 2065.85 | 1301.67 | 2830.04 |

#### 3.4.2. Average training and validation curves for AEC and scMEDAL-FE across 5 folds in the Healthy Heart, ASD, and AML datasets

**Figs.** S9-S11 present the training and validation curves, for the AEC and scMEDAL-FEC models in the three datasets. For the AEC model, the total loss is calculated as the sum of the reconstruction loss and the cell type classification loss. For scMEDAL-FEC, the total loss is calculated as the sum of the reconstruction loss and the cell type classification loss minus the adversarial loss. We observe that in all cases the individual terms have similar value ranges and contribute effectively to the total loss.

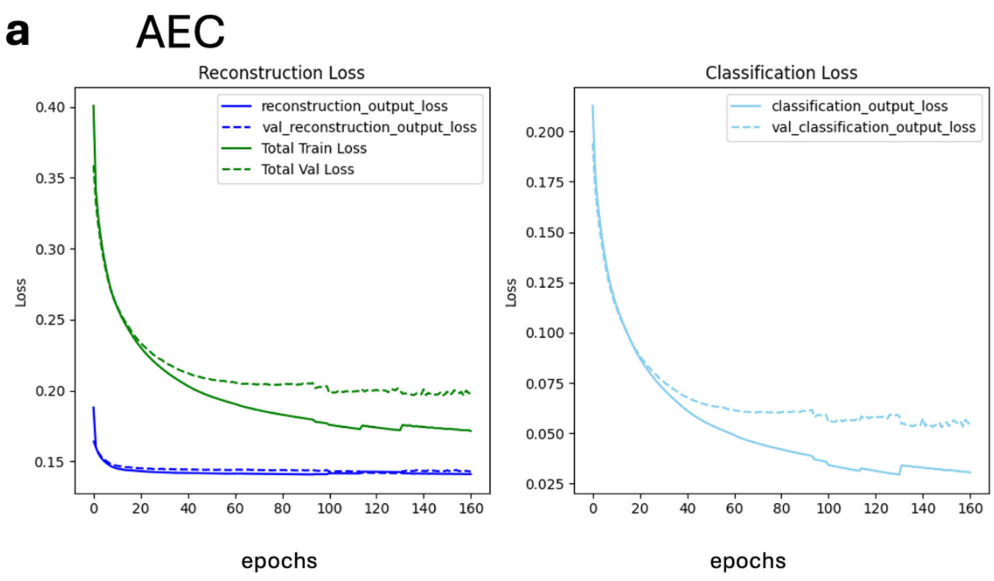

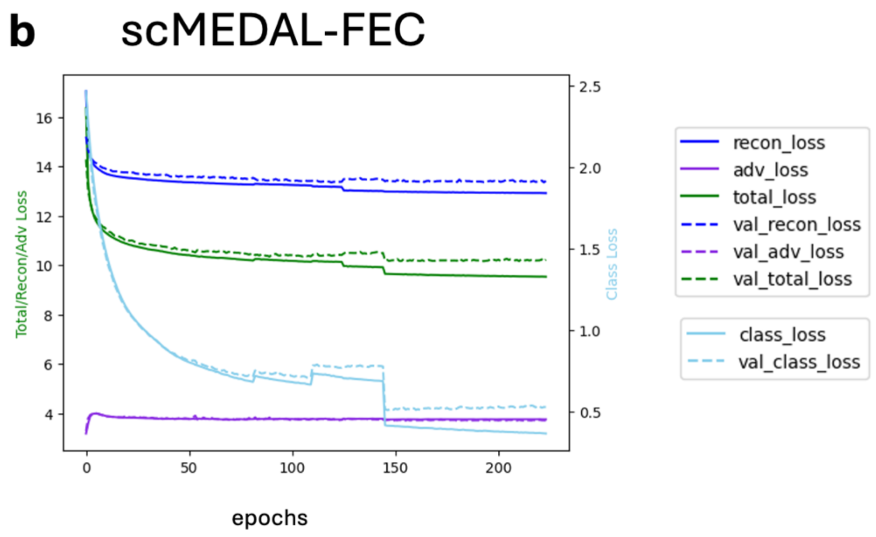

**Fig. S9: The training and validation curves for the Healthy Heart dataset demonstrate that the weight-adjusted individual loss terms effectively balance their contributions to the total loss.** Training and validation curves for the Healthy Heart dataset for **a** AEC and **b** scMEDAL-FEC models.

**a** AEC

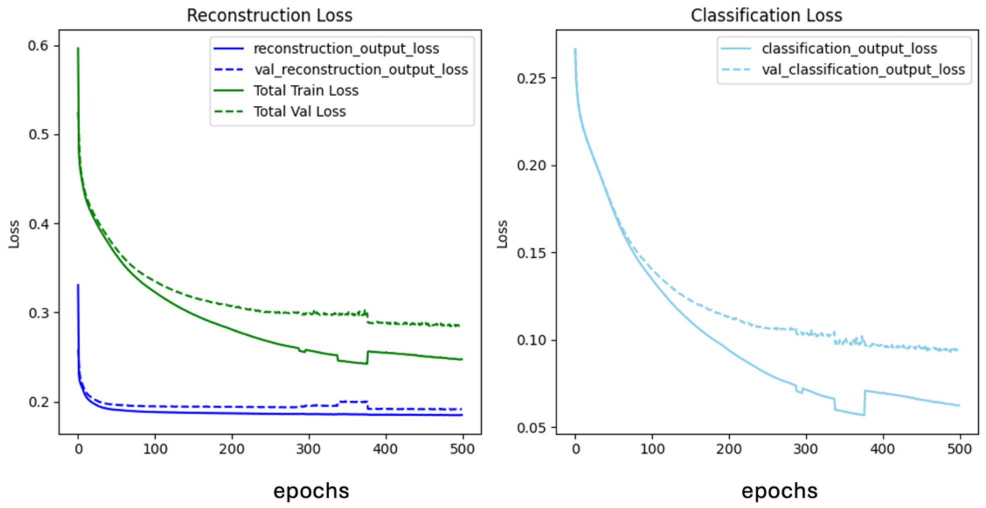

**b** scMEDAL-FEC

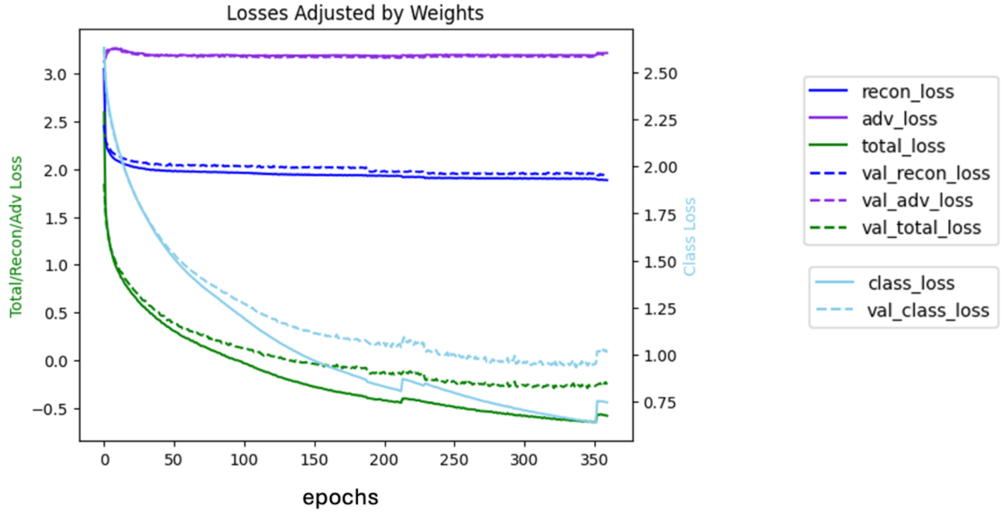

**Fig. S10: The training and validation curves for the ASD dataset demonstrate that the weight-adjusted individual loss terms effectively balance their contributions to the total loss.** Training and validation curves for the ASD dataset for **a** AEC and **b** scMEDAL-FEC models.

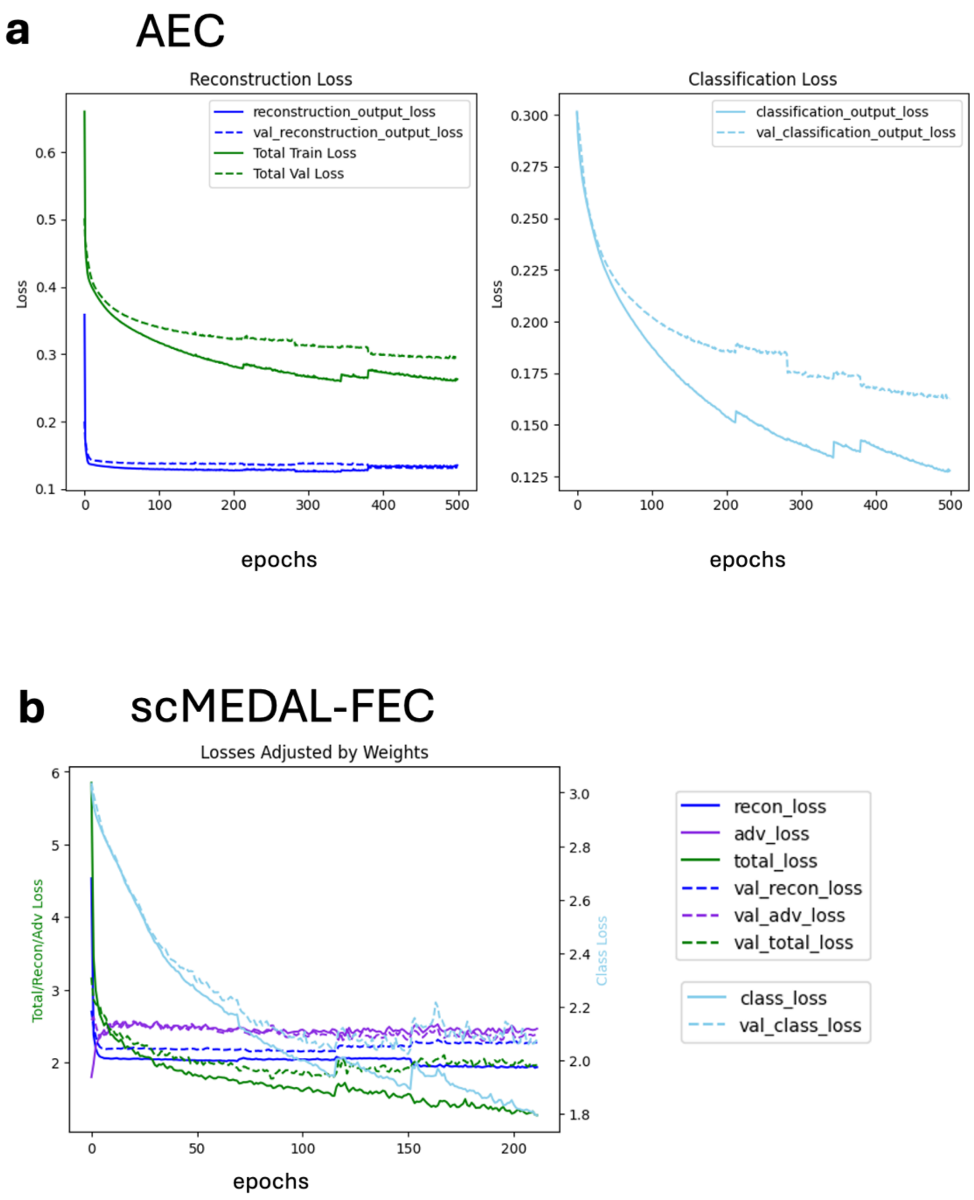

**Fig. S11: The training and validation curves for the AML dataset demonstrate that the weight-adjusted individual loss terms effectively balance their contributions to the total loss.** Training and validation curves for the AML dataset for **a** AEC and **b** scMEDAL-FEC models.

### 3.5. Significant genes from the genomaps for AML *versus* controls and ASD *versus* controls

**Table** S13 presents the statistics for significant genes related to ASD, identified using the scMEDAL-RE projection of the same 300 L2/3 cells across 15 ASD donors and then across 16 control (typically developing) donors. Statistical analysis of the gene expression between these groups was conducted using linear mixed-effects models implemented in Statsmodels[14]. A total of 230 genes were identified as significantly associated with ASD ($p_{slope} < 0.05$). Statistics for these ASD-relevant genes are illustrated below. The full list of all ASD mapped genomap genes is available in Supplementary Data 1.

**Table S13.** Statistics for relevant genes associated with ASD. These genes are identified as significant through linear mixed-effects modeling comparing scMEDAL-RE projections of the same 300 L2/3 cells across 15 ASD and 16 control donors.

| genes | pixel_i | pixel_j | intercept | p-val intercept | 95% CI (intercept) | | slope (control) | p-val slope | 95% CI (slope) | |
|---|---|---|---|---|---|---|---|---|---|---|
| CNR1 | 1 | 1 | 0.3946 | 0.0048 | 0.1206 | 0.6686 | -0.7758 | 0.0001 | -1.1572 | -0.3944 |
| MYO1E | 5 | 15 | -0.2923 | 0.0752 | -0.6144 | 0.0297 | 0.6037 | 0.0083 | 0.1554 | 1.052 |
| CYP27A1 | 4 | 27 | -0.2552 | 0.0801 | -0.541 | 0.0306 | 0.5158 | 0.0111 | 0.118 | 0.9136 |
| KCNJ10 | 48 | 15 | -0.3308 | 0.1317 | -0.761 | 0.0993 | 0.6454 | 0.0346 | 0.0466 | 1.2441 |
| THBS1 | 17 | 34 | -0.1595 | 0.0946 | -0.3465 | 0.0275 | 0.2753 | 0.0382 | 0.015 | 0.5356 |
| NOTCH3 | 5 | 34 | -0.259 | 0.147 | -0.6091 | 0.0911 | 0.5295 | 0.0332 | 0.0422 | 1.0167 |
| IL1R1 | 40 | 15 | -0.1641 | 0.1696 | -0.3984 | 0.0701 | 0.3533 | 0.0337 | 0.0272 | 0.6793 |
| COL4A1 | 17 | 3 | -0.3247 | 0.0165 | -0.5901 | -0.0593 | 0.6232 | 0.0009 | 0.2538 | 0.9926 |
| TGM2 | 10 | 11 | -0.2724 | 0.1728 | -0.664 | 0.1192 | 0.5951 | 0.0324 | 0.05 | 1.1402 |
| FGF2 | 49 | 16 | -0.3256 | 0.149 | -0.7678 | 0.1167 | 0.6917 | 0.0277 | 0.0761 | 1.3072 |

**Table** S14 presents the statistics for significant genes related to AML. These are identified by a Mann-Whitney U test performed on the same set of 300 monocytes projected with scMEDAL-RE onto 12 AML donors and then onto 5 healthy control subjects. Averages from the AML subjects and the control (healthy) subjects were computed for each set of 300 monocytes, and *stats* module from SciPy[13]was used to conduct the Mann-Whitney U test to identify gene expression differences between these groups. In total, 358 genes were found to have significantly different expression levels ($p < 0.05$). The full list of all AML mapped genomap genes is available in Supplementary Data 1.

**Table S14.** Statistics for relevant genes associated with AML, identified as significant through a Mann-Whitney U test on the same 300 monocyte Genomap projections from 12 AML scMEDAL-RE analyses and 5 control scMEDAL-RE analyses.

| genes | pixel_i | pixel_j | p-val |
|---|---|---|---|
| SH2B3 | 24 | 36 | 0.0023 |
| HERPUD1 | 3 | 19 | 0.0194 |
| PRDM1 | 2 | 23 | 0.0365 |
| MKI67 | 11 | 27 | 0.0365 |
| GATA2 | 45 | 46 | 0.0365 |
| ABCB1 | 30 | 43 | 0.0485 |
| SAMD9L | 30 | 40 | 0.0194 |
| SERPINA1 | 4 | 44 | 0.0013 |
| SLC11A1 | 20 | 28 | 0.0094 |
| VNN2 | 17 | 27 | 0.0365 |

## References

1 Litvinukova, M., Talavera-Lopez, C., Maatz, H., Reichart, D., Worth, C. L., Lindberg, E. L., Kanda, M., Polanski, K., Heinig, M., Lee, M., Nadelmann, E. R., Roberts, K., Tuck, L., Fasouli, E. S., DeLaughter, D. M., McDonough, B., Wakimoto, H., Gorham, J. M., Samari, S., Mahbubani, K. T., Saeb-Parsy, K., Patone, G., Boyle, J. J., Zhang, H., Viveiros, A., Oudit, G. Y., Bayraktar, O. A., Seidman, J. G., Seidman, C. E., Noseda, M., Hubner, N. & Teichmann, S. A. Cells of the adult human heart. *Nature* **588**, 466-472 (2020).

2 Yu, X., Xu, X., Zhang, J. & Li, X. Batch alignment of single-cell transcriptomics data using deep metric learning. figshare https://doi.org/10.6084/m9.figshare.20499630.v2 (2023).

3 Velmeshev, D., Schirmer, L., Jung, D., Haeussler, M., Perez, Y., Mayer, S., Bhaduri, A., Goyal, N., Rowitch, D. H. & Kriegstein, A. R. Single-cell genomics identifies cell type-specific molecular changes in autism. *Science* **364**, 685-689 (2019).

4 van Galen, P., Hovestadt, V., Wadsworth Ii, M. H., Hughes, T. K., Griffin, G. K., Battaglia, S., Verga, J. A., Stephansky, J., Pastika, T. J., Lombardi Story, J., Pinkus, G. S., Pozdnyakova, O., Galinsky, I., Stone, R. M., Graubert, T. A., Shalek, A. K., Aster, J. C., Lane, A. A. & Bernstein, B. E. Single-Cell RNA-Seq Reveals AML Hierarchies Relevant to Disease Progression and Immunity. *Cell* **176**, 1265-1281.e1224 (2019).

5 Yu, X., Xu, X., Zhang, J. & Li, X. Batch alignment of single-cell transcriptomics data using deep metric learning. *Nat. Commun.* **14**, 960 (2023).

6 Dai, C., Chen, M., Wang, C. & Hao, X. Deconvolution of Bulk Gene Expression Profiles with Single-Cell Transcriptomics to Develop a Cell Type Composition-Based Prognostic Model for Acute Myeloid Leukemia. *Front Cell Dev Biol* **9**, 762260 (2021).

7 Abadi, M., Agarwal, A., Barham, P., Brevdo, E., Chen, Z., Citro, C., Corrado, G. S., Davis, A., Dean, J., Devin, M., Ghemawat, S., Goodfellow, I., Harp, A., Irving, G., Isard, M., Jozefowicz, R., Jia, Y., Kaiser, L., Kudlur, M., Levenberg, J., Mané, D., Schuster, M., Monga, R., Moore, S., Murray, D., Olah, C., Shlens, J., Steiner, B., Sutskever, I., Talwar, K., Tucker, P., Vanhoucke, V., Vasudevan, V., Viégas, F., Vinyals, O., Warden, P., Wattenberg, M., Wicke, M., Yu, Y. & Zheng, X. TensorFlow: Large-scale machine learning on heterogeneous systems. *Proc. of 12th USENIX Conference on Operating Systems Design and Implementation* (2016).

8 Dillon, J. V., Langmore, I., Tran, D., Brevdo, E., Vasudevan, S., Moore, D., Patton, B., Alemi, A., Hoffman, M. & Saurous, R. A. TensorFlow Distributions. arXiv:1711.10604, 2017.

9 TensorFlow. *TensorFlow Probability*, https://www.tensorflow.org/probability , last accessed 2/12/2025

10 Pedregosa, F., Eickenberg, M., Ciuciu, P., Thirion, B. & Gramfort, A. Data-driven HRF estimation for encoding and decoding models. *Neuroimage* **104**, 209-220 (2015).

11 McInnes, L., Healy, J. & Melville, J. UMAP: Uniform Manifold Approximation and Projection for Dimension Reduction. Preprint at https://doi.org/10.48550/arXiv.1802.03426 (2018).

12 Wolf, F. A., Angerer, P. & Theis, F. J. SCANPY: large-scale single-cell gene expression data analysis. *Genome Biol.* **19**, 15 (2018).

13 Virtanen, P., Gommers, R., Oliphant, T. E., Haberland, M., Reddy, T., Cournapeau, D., Burovski, E., Peterson, P., Weckesser, W., Bright, J., van der Walt, S. J., Brett, M., Wilson, J., Millman, K. J., Mayorov, N., Nelson, A. R. J., Jones, E., Kern, R., Larson, E., Carey, C. J., Polat, I., Feng, Y., Moore, E. W., VanderPlas, J., Laxalde, D., Perktold, J., Cimrman, R., Henriksen, I., Quintero, E. A., Harris, C. R., Archibald, A. M., Ribeiro, A. H., Pedregosa, F., van Mulbregt, P. & SciPy, C. SciPy 1.0: fundamental algorithms for scientific computing in Python. *Nat Methods* **17**, 261-272 (2020).

14 Seabold, S. & Perktold, J. *Statsmodels: Econometric and Modeling with Python* in *Proc. 9th Python in Science Conference.* 57-61 (2010).

15 Rousseeuw, P. J. Silhouettes: A graphical aid to the interpretation and validation of cluster analysis. *J. Comput. Appl. Math.* **20**, 53-65 (1987).

16 Calinski, T. & Harabasz, J. A dendrite method for cluster analysis. *Commun. Stat. Theory Methods* **3**, 1-27 (1974).

17 Davies, D. L. & Bouldin, D. W. A Cluster Separation Measure. *IEEE Trans. Pattern Anal. Mach. Intell.* **1**, 224-227 (1979).
