# Supplementary material for: Mixed Effects Deep Learning for the interpretable analysis of single cell RNA sequencing data by quantifying and visualizing batch effects
Source: ArXiv. 2024 Nov 13:arXiv:2411.06635v2. Preprint. [Version 2] (PMC11601787)
Supplement: Supplementary file 1 [file NIHPP2411.06635v2-supplement-1.pdf]

Mixed Effects Deep Learning for the interpretable analysis of single cell RNA sequencing data by quantifying and visualizing batch effects

Supplemental

Table of Contents

Supplemental Methods **2**

Hyperparameters used on the AE, AEC, MEDL-AE-FE, MEDL-AEC-FE, and MEDL-AE-RE models *2*

Data Preprocessing pipeline *5*

UMAP Visualization parameters *5*

Hardware *5*

Supplemental Results **6**

Supplementary Figures for Dataset Description *6*
Donor Representation Across Cell Types in AML dataset 6

Hyperparameter Optimization for Experiments 1 A) and 3 .... *7*
ASW Scores for Batch and Cell Type Separability 7

Supplementary Results for Experiment 1 A) *8*
CH and 1/DB scores 8
Random Effects Subnetwork (MEDL-AE-RE) latent spaces 9
Average Training and Validation Curves for AE, MEDL-AE-FE, and MEDL-AE-RE Across 5 Folds in the Healthy Heart, ASD, and AML Datasets 11

Supplementary Results for Experiment 3 .... *14*
CH and 1/DB scores 14
Average Training and Validation Curves for AEC and MEDL-AEC-FE Across 5 Folds in the Healthy Heart, ASD, and AML Datasets 15

References **17**

Supplemental Methods

Hyperparameters used on the AE, AEC, MEDL-AE-FE, MEDL-AEC-FE, and MEDL-AE-RE models

In Table S1-S3 there are described the Hyperparameters for data loading, model construction, compilation, and training were applied across the AE, AEC, MEDL-AE-FE, MEDL-AEC-FE, and MEDL-AE-RE models in three datasets: Healthy Heart, the Autism Spectrum Disorder (ASD) and control dataset, and Acute Myeloid Leukemia (AML) and healthy.

Table S1. Hyperparameters and parameters for data loading, model building, compilation, and training in the Healthy Heart Dataset.

Settings	Hyperparameter/Parameter	AE	AEC	MEDL-AE-FE	MEDL-AEC-FE	MEDL-AE-RE
Compile model	optimizer 1	Adam(lr=0.0001)				
	optimizer 2 (adversarial)	-	-	Adam(lr=0.0001)	Adam(lr=0.0001)	-
	reconstruction loss (X)	MSE				
	adversarial loss (z)	-	-	CCE	CCE	-
	classification loss (y)	-	CCE	-	CCE	-
	classification loss (z)	-	-	-	-	CCE
	reconstruction loss_weights (X)	1	81	5400	9450	110
	classification loss_weights (y)	-	0.1	-	1	-
	adversarial loss weights (z)	-	-	1	1	-
	cluster loss weight (z)	-				0.1
Build model	kl_weight (z)	-				1.00E-05
	post_loc_init_scale (z)	-				0.1
	prior_scale (z)	-				0.25
	n_latent_dims	2				
	layer_units	[512,132]				
	layer_units_latent_classifier	-	[2]	-	[2]	[5]
	n_clusters	-	-	147	147	147
	n_pred (y)	-	13	-	13	-
	last_activation	linear				
	use_batch_norm	TRUE				default in RE layers
	dense hidden layers activation	selu				
	classifier activation	softmax				
Load data	use_z	FALSE	FALSE	TRUE	TRUE	TRUE
	get_pred (y)	FALSE	TRUE	FALSE	TRUE	FALSE
	scaling	min_max				
	batch_size	512				
Train model	epochs	500				
	monitor_metric	val_loss	val_loss	val_total_loss	val_total_loss	val_total_loss
	patience	30				
	stop_criteria	early_stopping				

Table S2. Hyperparameters and parameters for data loading, model building, compilation, and training in the ASD Dataset.

Settings	Hyperparameter/Parameter	AE	AEC	MEDL-AE-FE	MEDL-AEC-FE	MEDL-AE-RE
Compile model	optimizer 1	Adam(lr=0.0001)				
	optimizer 2 (adversarial)	-	-	Adam(lr=0.0001)	Adam(lr=0.0001)	-
	reconstruction loss (X)	MSE				
	adversarial loss (z)	-	-	CCE	CCE	-
	classification loss (y)	-	CCE	-	CCE	-
	classification loss (z)	-	-	-	-	CCE
	reconstruction loss_weights (X)	1	1	1000	1000	110
	classification loss_weights (y)	-	0.1	-	1	-
	adversarial loss weights (z)	-	-	1	1	-
	cluster loss weight (z)	-				0.1
Build model	kl_weight (z)	-				1.00E-05
	post_loc_init_scale (z)	-				0.1
	prior_scale (z)	-				0.25
	n_latent_dims	2				
	layer_units	[512,132]				
	layer_units_latent_classifier	NA	[2]	-	[2]	[5]
	n_clusters	-	-	31	31	31
	n_pred (y)	NA	17	-	17	17
	last_activation	linear				
	use_batch_norm	TRUE				default in RE layers
	dense hidden layers activation	selu				
	classifier activation	softmax				
Load data	use_z	FALSE	FALSE	TRUE	TRUE	TRUE
	get_pred (y)	FALSE	TRUE	FALSE	TRUE	FALSE
	scaling	min_max				
	batch_size	512				
Train model	epochs	500				
	monitor_metric	val_loss	val_loss	val_total_loss	val_total_loss	val_total_loss
	patience	30				
	stop_criteria	early_stopping				

Table S3. Hyperparameters and parameters for data loading, model building, compilation, and training in the AML Dataset.

Settings	Hyperparameter/Parameter	AE	AEC	MEDL-AE-FE	MEDL-AEC-FE	MEDL-AE-RE
Compile model	optimizer 1	Adam(lr=0.0001)				
	optimizer 2 (adversarial)	-	-	Adam(lr=0.0001)	Adam(lr=0.0001)	-
	reconstruction loss (X)	MSE				
	adversarial loss (z)	-	-	CCE	CCE	-
	classification loss (y)	-	CCE	-	CCE	-
	classification loss (z)	-	-	-	-	CCE
	reconstruction loss_weights (X)	1	100	4000	1500	110
	classification loss_weights (y)	-	0.1	-	1	-
	adversarial loss weights (z)	-	-	1	1	-
	cluster loss weight (z)	-				0.1
Build model	kl_weight (z)	-				1.00E-05
	post_loc_init_scale (z)	-				0.1
	prior_scale (z)	-				0.25
	n_latent_dims	2				
	layer_units	[512,132]				
	layer_units_latent_classifier	-	[2]	-	[2]	[5]
	n_clusters	-	-	19	19	19
	n_pred (y)	-	21	-	21	-
	last_activation	linear				
	use_batch_norm	TRUE				default in RE layers
	dense hidden layers activation	selu				
	classifier activation	softmax				
Load data	use_z	FALSE	FALSE	TRUE	TRUE	TRUE
	get_pred (y)	FALSE	TRUE	FALSE	TRUE	FALSE
	scaling	min_max				
	batch_size	512				
Train model	epochs	500				
	monitor_metric	val_loss	val_loss	val_total_loss	val_total_loss	val_total_loss
	patience	30				
	stop_criteria	early_stopping				

Data Preprocessing pipeline

We used standard preprocessing steps from Yu et al. , 2023 (Yu et al., 2023): 1) filtering out cells exhibiting expression in fewer than ten genes and removed genes detected in fewer than three cells, 2) normalizing total Unique molecular identifiers (UMI) counts per cell to 10,000, 3) log-transforming the data to stabilize variance $\log(X+1)$, and 4) selecting the top highly variable genes (HVGs).

UMAP Visualization parameters

In Table S4 are described the minimum distance and nearest neighbors choices for UMAP visualizations.

Table S4. UMAP Visualization parameters.

<table>
<tr><th>Dataset</th><th>UMAP parameters</th><th>AE</th><th>AEC</th><th>MEDL-AE-FE</th><th>MEDL-AEC-FE</th><th>MEDL-AE-RE</th></tr>
<tr><td rowspan="2">Healthy Heart</td><td>Min distance</td><td colspan="4">0.5</td><td>0</td></tr>
<tr><td>Nearest Neighbors</td><td colspan="4">15</td><td>1000</td></tr>
<tr><td rowspan="2">ASD</td><td>Min distance</td><td colspan="5">0.5</td></tr>
<tr><td>Nearest Neighbors</td><td colspan="5">15</td></tr>
<tr><td rowspan="2">AML</td><td>Min distance</td><td colspan="5">0.5</td></tr>
<tr><td>Nearest Neighbors</td><td colspan="5">15</td></tr>
</table>

Hardware

Our MEDL subnetworks, particularly the Random Effects subnetwork, are complex models that require graphics processing unit (GPU) for training. We used a Tesla V100 GPU with 32GB of memory and Tesla P4 GPU with 8GB of memory to train our models. However, the Fixed Effects subnetworks and autoencoder models are less computationally demanding and can be trained on the Tesla P4 GPU with 8GB of memory.

Supplemental Results

Supplementary Figures for Dataset Description

Donor Representation Across Cell Types in AML dataset

Fig. S1 illustrates the distribution of donors across each cell type. As observed, not all donor samples contain cells from every cell type, leading to potential confounding between donor identity and cell type.

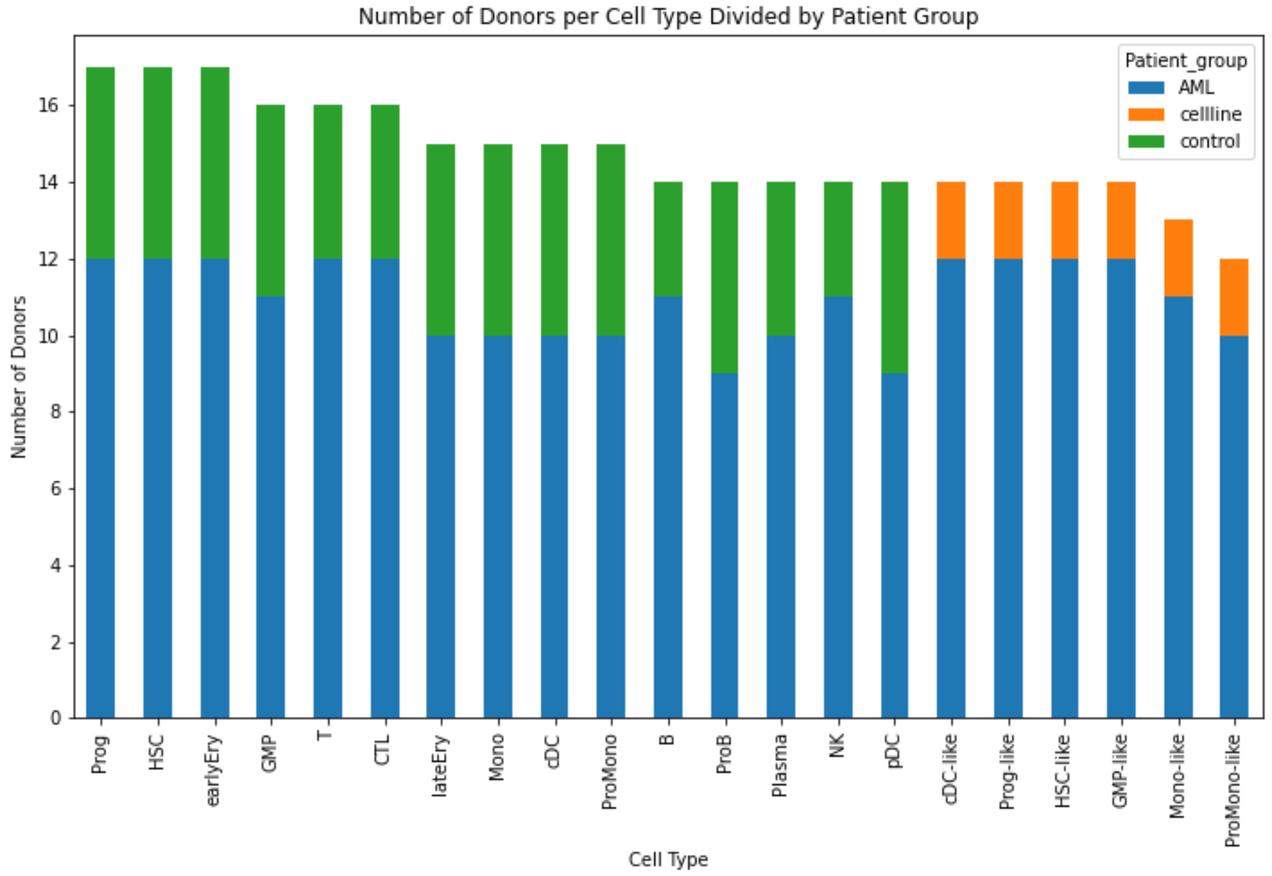

Fig. S1. AML dataset cell type-donor confounding. Number of donors per each celltype divided by patient group.

Hyperparameter Optimization for Experiments 1 A) and 3

ASW Scores for Batch and Cell Type Separability

Hyperparameter optimization (HPO) was conducted manually on a case-by-case basis to maximize the Average Silhouette Width (ASW) for cell type. Tables S5 and S6 present ASW scores for both batch and cell type separability for different values of the weights assigned to the reconstruction loss (mean squared error, MSE), adversarial loss, and classification loss of the cell type labels y. These results were derived from the validation sets of the ASD and AML datasets.

Table S5. Average Silhouette Width (ASW) Scores for Batch and Cell Type Separability of **ASD dataset** (Mean Across 5 Folds). Hyperparameter Optimization (HPO) on reconstruction loss weight λ_{MSE} on validation dataset. Adversarial loss weight $\lambda_A = 1$ for all models.

	λ_{MSE}	λ_A	ASW (batch)			ASW (cell type)		
			mean	95% CI		mean	95% CI	
MEDL-AE-FE	10	1	-0.27	-0.35	-0.19	0.01	-0.10	0.12
MEDL-AE-FE	100		-0.27	-0.32	-0.21	0.08	0.04	0.12
MEDL-AE-FE	**1000**		**-0.23**	**-0.28**	**-0.19**	**0.20**	**0.16**	**0.24**

Table S6. Average Silhouette Width (ASW) Scores for Batch and Cell Type Separability of the **AML dataset** (Mean Across 5 Folds). Hyperparameter Optimization (HPO) on reconstruction loss λ_{MSE} and classification loss λ_{CCE_y} weights for validation. Adversarial loss weight $\lambda_A = 1$ for all models.

	λ_{MSE}	λ_{CCE_y}	ASW (batch)			ASW (cell type)		
			mean	95% CI		mean	95% CI	
MEDL-AE-FE	100	NA	-0.24	-0.25	-0.23	-0.14	-0.17	-0.11
MEDL-AE-FE	500		-0.28	-0.30	-0.26	-0.12	-0.15	-0.08
MEDL-AE-FE	1000		-0.30	-0.33	-0.28	-0.12	-0.13	-0.10
MEDL-AE-FE	2000		-0.28	-0.30	-0.27	-0.10	-0.13	-0.08
MEDL-AE-FE	3000		-0.28	-0.30	-0.27	-0.06	-0.08	-0.04
**MEDL-AE-FE**	**4000**		**-0.29**	**-0.31**	**-0.28**	**-0.05**	**-0.07**	**-0.04**
**AEC**	**1**	0.1	-0.43	-0.52	-0.34	-0.15	-0.23	-0.06
AEC	10		-0.37	-0.44	-0.31	-0.11	-0.20	-0.02
AEC	100		**-0.28**	**-0.34**	**-0.23**	**0.03**	**-0.04**	**0.10**
MEDL-AEC-FE	500	1	-0.33	-0.35	-0.31	-0.10	-0.13	-0.06
**MEDL-AEC-FE**	**1500**	**1**	**-0.30**	**-0.37**	**-0.24**	**-0.05**	**-0.08**	**-0.02**
MEDL-AEC-FE	1500	2	-0.33	-0.40	-0.26	-0.07	-0.11	-0.04
MEDL-AEC-FE	2000	2	-0.31	-0.33	-0.28	-0.07	-0.13	-0.01

Supplementary Results for Experiment 1 A)

CH and 1/DB scores

In tables S7-S9, the CH and 1/DB scores are bolded when they correspond to the top-ranked model based on the ASW result, even if their values differ. The bolding indicates that, despite variations in the CH and 1/DB values, these models maintain the top performance ranking across all metrics for cell type preservation.

Table S7. 1/DB and CH scores (Mean and 95% CI across 5 folds) for batch and cell type separability in the latent spaces of the Healthy Heart Dataset, using PCA, AE, MEDL-AE-FE, and MEDL-AE-RE models.

	batch						cell type					
	1/DB			CH			1/DB			CH		
	mean	95% CI		mean	95% CI		mean	95% CI		mean	95% CI	
PCA (Baseline)	0.02	0.01	0.03	148.65	145.33	151.98	0.13	0.10	0.15	1502.74	1456.79	1548.69
AE	0.02	0.02	0.02	70.49	51.09	89.90	**0.22**	**0.10**	**0.33**	1712.76	1405.70	2019.83
MEDL-AE-FE	0.02	0.01	0.02	**61.38**	**38.74**	**84.03**	**0.19**	**0.13**	**0.24**	1984.01	1078.08	2889.95
MEDL-AE-RE	**0.56**	**0.17**	**0.94**	**2555.27**	**544.02**	**4566.51**	0.06	0.04	0.08	224.76	66.19	383.34

Table S8. 1/DB and CH scores (Mean and 95% CI across 5 folds) for batch and cell type separability in the latent spaces of the ASD Dataset, using PCA, AE, MEDL-AE-FE, and MEDL-AE-RE models.

	batch						cell type					
	1/DB			CH			1/DB			CH		
	mean	95% CI		mean	95% CI		mean	95% CI		mean	95% CI	
PCA (Baseline)	0.03	0.02	0.03	32.67	32.06	33.28	0.18	0.15	0.21	9089.94	8906.86	9273.02
AE	0.02	0.02	0.03	23.72	20.74	26.69	**0.78**	**0.71**	**0.85**	**9863.14**	**8673.06**	**11053.22**
MEDL-AE-FE	0.02	0.02	0.03	19.43	14.12	24.74	**0.32**	**0.15**	**0.49**	**9631.75**	**8052.25**	**11211.25**
MEDL-AE-RE	**0.16**	**-0.09**	**0.40**	**2423.99**	**-3510.80**	**8358.77**	0.03	0.01	0.04	749.41	-261.64	1760.45

Table S9. 1/DB and CH scores (Mean and 95% CI across 5 folds) for batch and cell type separability in the latent spaces of the AML Dataset, using PCA, AE, MEDL-AE-FE, and MEDL-AE-RE models.

	batch						cell type					
	1/DB			CH			1/DB			CH		
	mean	95% CI		mean	95% CI		mean	95% CI		mean	95% CI	
PCA (Baseline)	0.06	0.05	0.07	156.52	154.20	158.84	0.14	0.12	0.16	2560.96	2482.14	2639.79
AE	0.07	0.07	0.08	189.78	169.23	210.32	**0.28**	**0.24**	**0.32**	1956.48	1779.39	2133.58
MEDL-AE-FE	0.07	0.06	0.09	**80.63**	**69.88**	**91.37**	0.19	0.09	0.29	1493.43	1353.85	1633.01
MEDL-AE-RE	**0.37**	**0.07**	**0.67**	**63228.21**	**9245.17**	**117211.25**	0.07	0.05	0.09	114.73	69.53	159.94

Random Effects Subnetwork (MEDL-AE-RE) latent spaces

Fig. S2–S4 illustrate the MEDL-AE-RE latent spaces using two visualization methods: (A) UMAP applied to the MEDL-AE-RE latent representations and (B) the raw MEDL-AE-RE representations. The UMAP visualizations introduce artifacts due to outliers present in the batch distributions. Specifically:

- Fig. S2 shows the latent space for the Healthy Heart dataset.
- Fig. S3 presents the latent space for the ASD dataset.
- Fig. S4 depicts the latent space for the AML dataset.

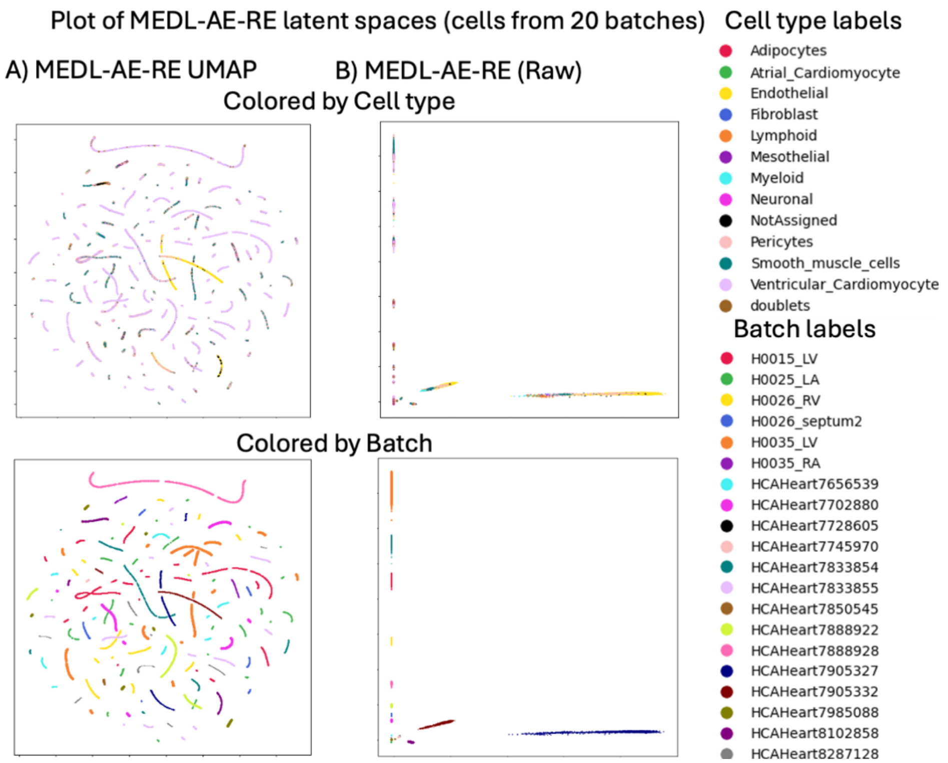

Fig. S2. Latent spaces from 44,987 cells from 20 out of 147 batches in the latent space from the Healthy Heart Dataset obtained with MEDL-AE-RE. A) UMAP applied to the MEDL-AE-RE latent space. B) Raw MEDL-AE-RE latent space.

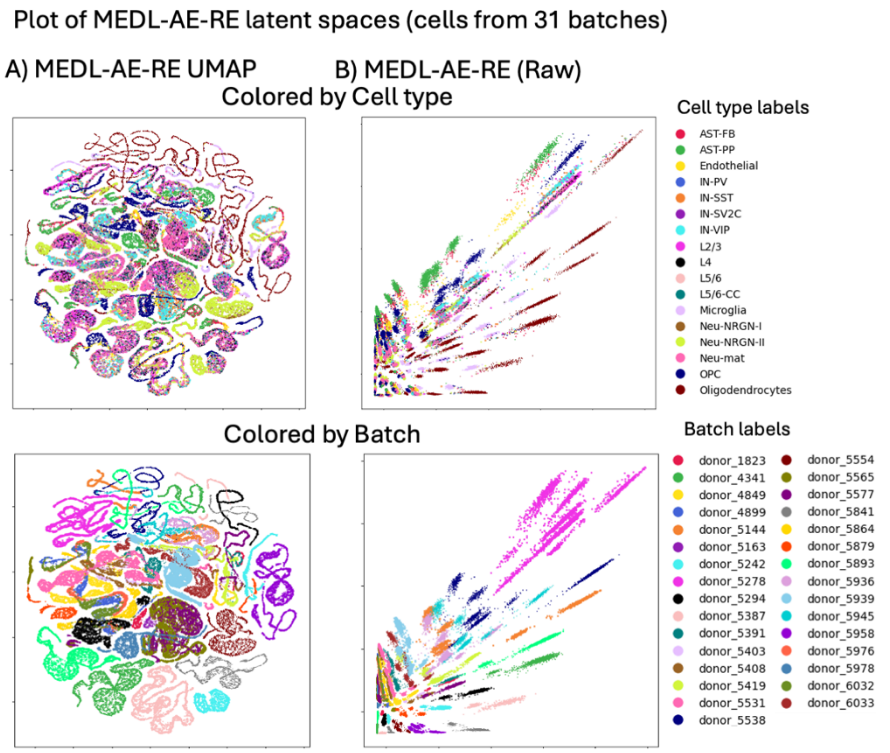

Fig. S3. Latent spaces from 62735 cells from ASD dataset latent spaces obtained with MEDL-AE-RE. A) UMAP applied to the MEDL-AE-RE latent space. B) Raw MEDL-AE-RE latent space.

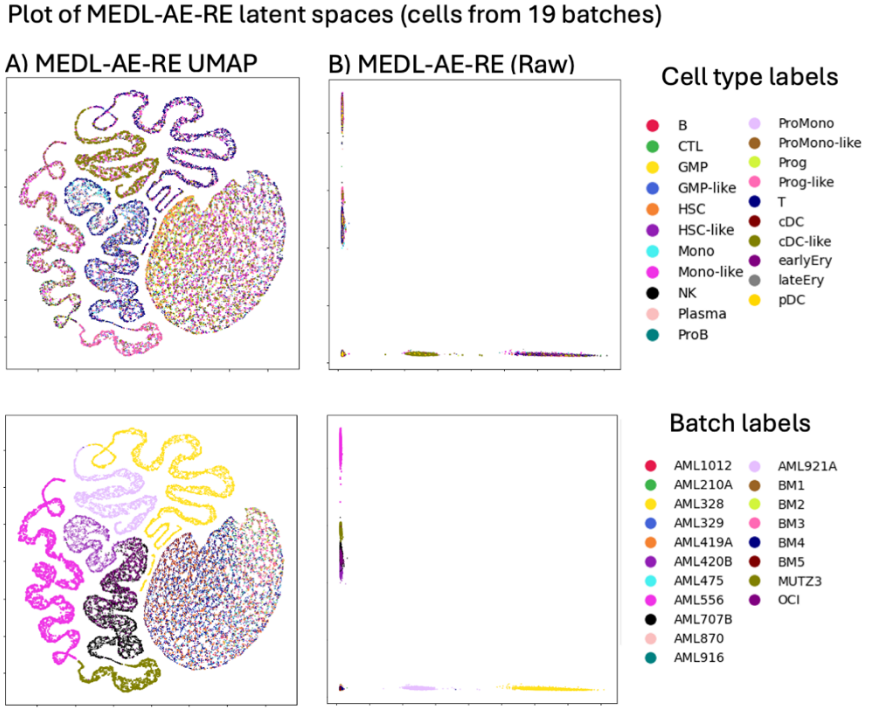

Fig. S4. Latent spaces from 23,050 cells from the AML dataset latent spaces obtained with MEDL-AE-RE. A) UMAP applied to the MEDL-AE-RE latent space. B) Raw MEDL-AE-RE latent space.

Average Training and Validation Curves for AE, MEDL-AE-FE, and MEDL-AE-RE Across 5 Folds in the Healthy Heart, ASD, and AML Datasets

Fig. S5-S7 present the training and validation curves, weighted accordingly, for the AE, MEDL-AE-FE, and MEDL-AE-RE models in all three datasets. For MEDL-AE-FE, the total loss is calculated as the reconstruction loss minus the adversarial loss, with each component adjusted by its respective weight. In the MEDL-AE-RE curves, the left axis displays the reconstruction loss and total loss, while the right axis represents the latent cluster loss, which classifies batch effects, and the Kullback-Leibler divergence (KLD) loss. The total loss is computed as the sum of the reconstruction loss, latent cluster loss, and KLD loss, each adjusted by their respective weights.

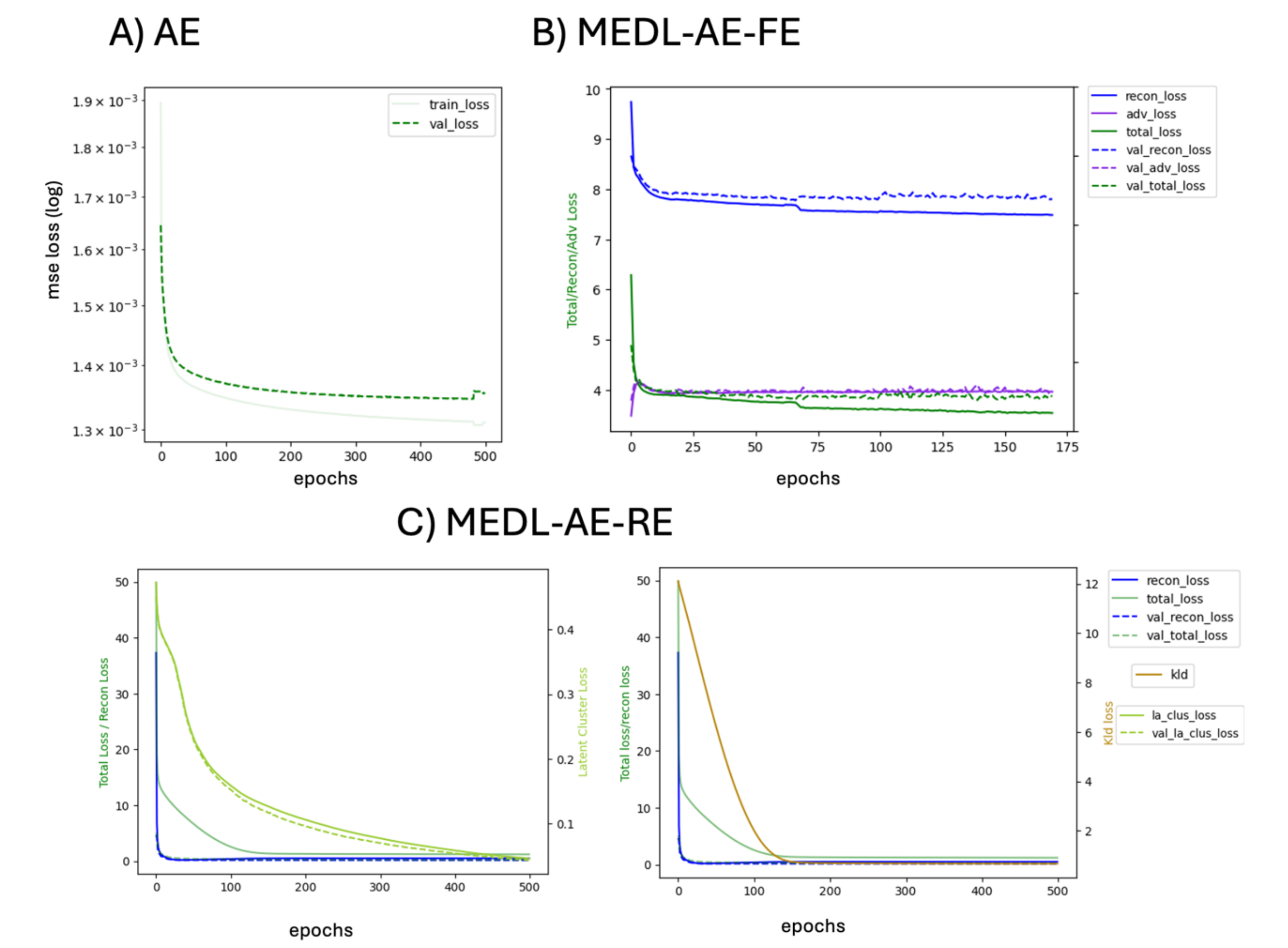

Fig. S5. Training and validation curves for the Healthy Heart dataset for the A) AE, B) MEDL-AE-FE and C) MEDL-AE-RE.

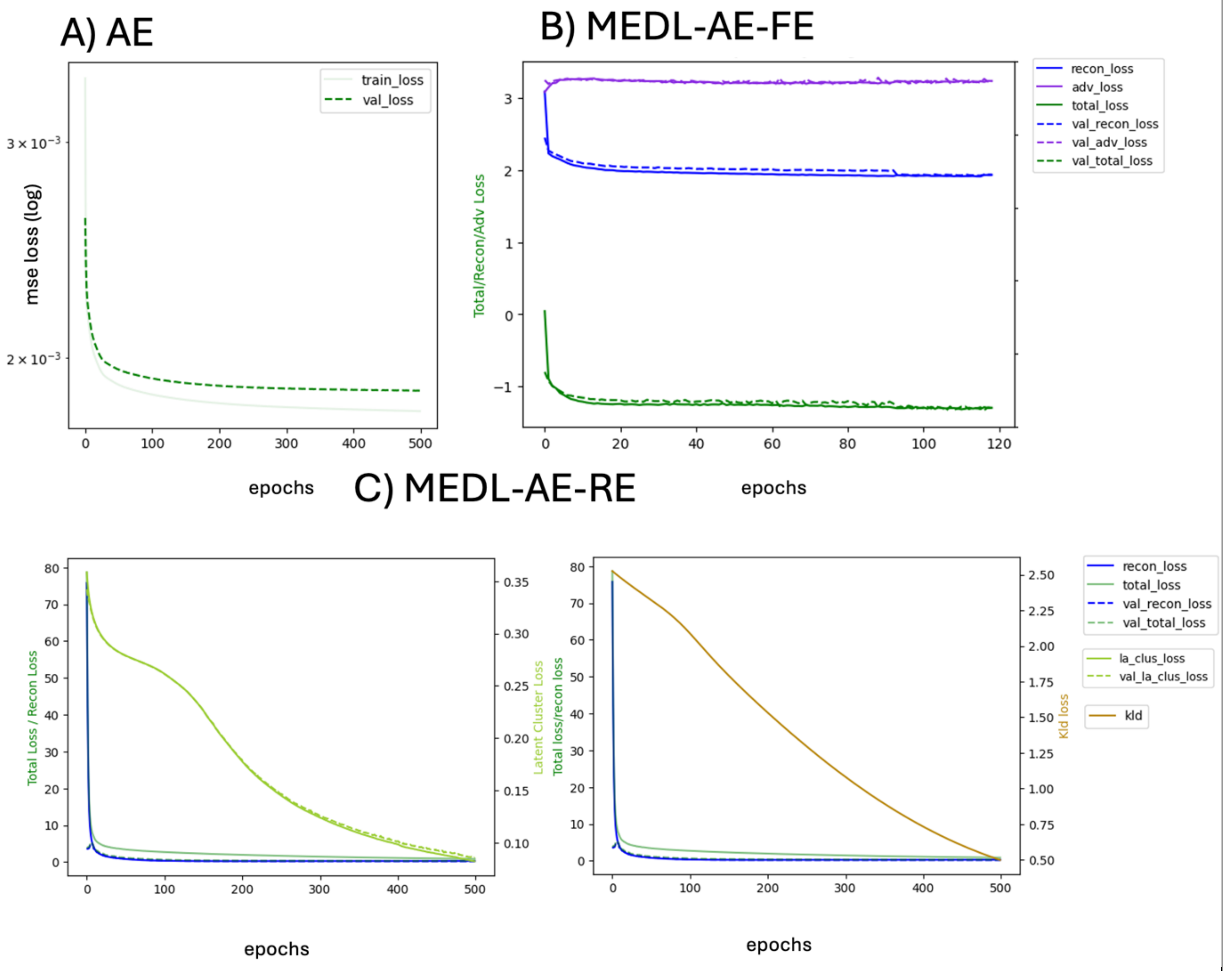

Fig. S6. Training and validation curves for the ASD dataset for the A) AE, B) MEDL-AE-FE and C) MEDL-AE-RE models.

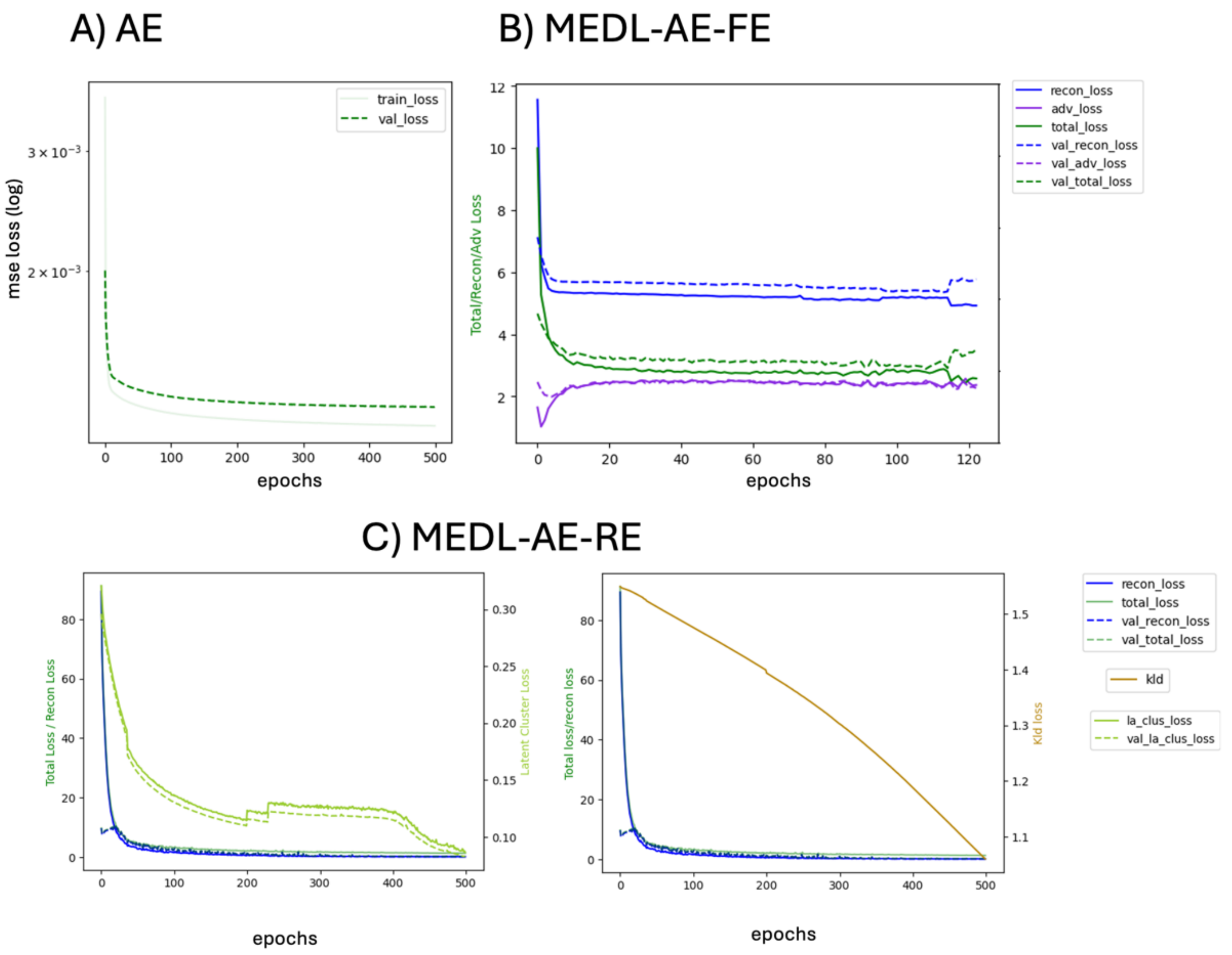

Fig. S7. Training and validation curves for the AML dataset for the A) AE, B) MEDL-AE-FE and C) MEDL-AE-RE models.

Supplementary Results for Experiment 3

CH and 1/DB scores

In tables S10-S12, the CH and 1/DB scores are bolded when they correspond to the top-ranked model based on the ASW result, even if their values differ. The bolding indicates that, despite variations in the CH and 1/DB values, these models maintain the top performance ranking across all metrics for cell type preservation.

Table S10. 1/DB and CH scores (Mean and 95% CI across 5 folds) for batch and cell type separability in the latent spaces of the Healthy Heart Dataset, using AEC and MEDL-AEC-FE models.

	batch						cell type					
	1/DB			CH			1/DB			CH		
	mean	95% CI		mean	95% CI		mean	95% CI		mean	95% CI	
AEC	0.02	0.01	0.03	**48.29**	**15.26**	**81.32**	**0.23**	**0.06**	**0.41**	**5563.96**	**2833.93**	**8293.99**
MEDL-AEC-FE	0.02	0.01	0.02	41.39	27.01	55.77	0.21	0.04	0.38	3750.68	2318.16	5183.19

Table S11. 1/DB and CH scores (Mean and 95% CI across 5 folds) for batch and cell type separability in the latent spaces of the ASD Dataset, using AEC and MEDL-AEC-FE models.

	batch						cell type					
	1/DB			CH			1/DB			CH		
	mean	95% CI		mean	95% CI		mean	95% CI		mean	95% CI	
AEC	0.02	0.01	0.03	20.06	14.48	25.64	**0.73**	**0.51**	**0.95**	**10631.01**	**5846.41**	**15415.61**
MEDL-AEC-FE	0.02	0.01	0.02	12.72	7.40	18.03	0.34	0.18	0.50	9911.04	8726.06	11096.02

Table S12. 1/DB and CH scores (Mean and 95% CI across 5 folds) for batch and cell type separability in the latent spaces of the AML, using AEC and MEDL-AEC-FE models.

	batch						cell type					
	1/DB			CH			1/DB			CH		
	mean	95% CI		mean	95% CI		mean	95% CI		mean	95% CI	
AEC	0.08	0.04	0.12	142.75	81.95	203.54	0.17	0.09	0.25	**2159.15**	**1216.68**	**3101.61**
MEDL-AEC-FE	0.06	0.04	0.07	87.24	70.03	104.45	0.18	0.10	0.25	2065.85	1301.67	2830.04

Average Training and Validation Curves for AEC and MEDL-AEC-FE Across 5 Folds in the Healthy Heart, ASD, and AML Datasets

Fig. S8-S10 present the training and validation curves, weighted accordingly, for the AEC and MEDL-AEC-FE models in all three datasets. For AEC the total loss is calculated as the sum of the reconstruction loss and the cell type classification loss, with each component adjusted by its respective weight. For MEDL-AEC-FE, the total loss is calculated as the sum of the reconstruction loss and the cell type classification loss minus the adversarial loss, with each component adjusted by its respective weight.

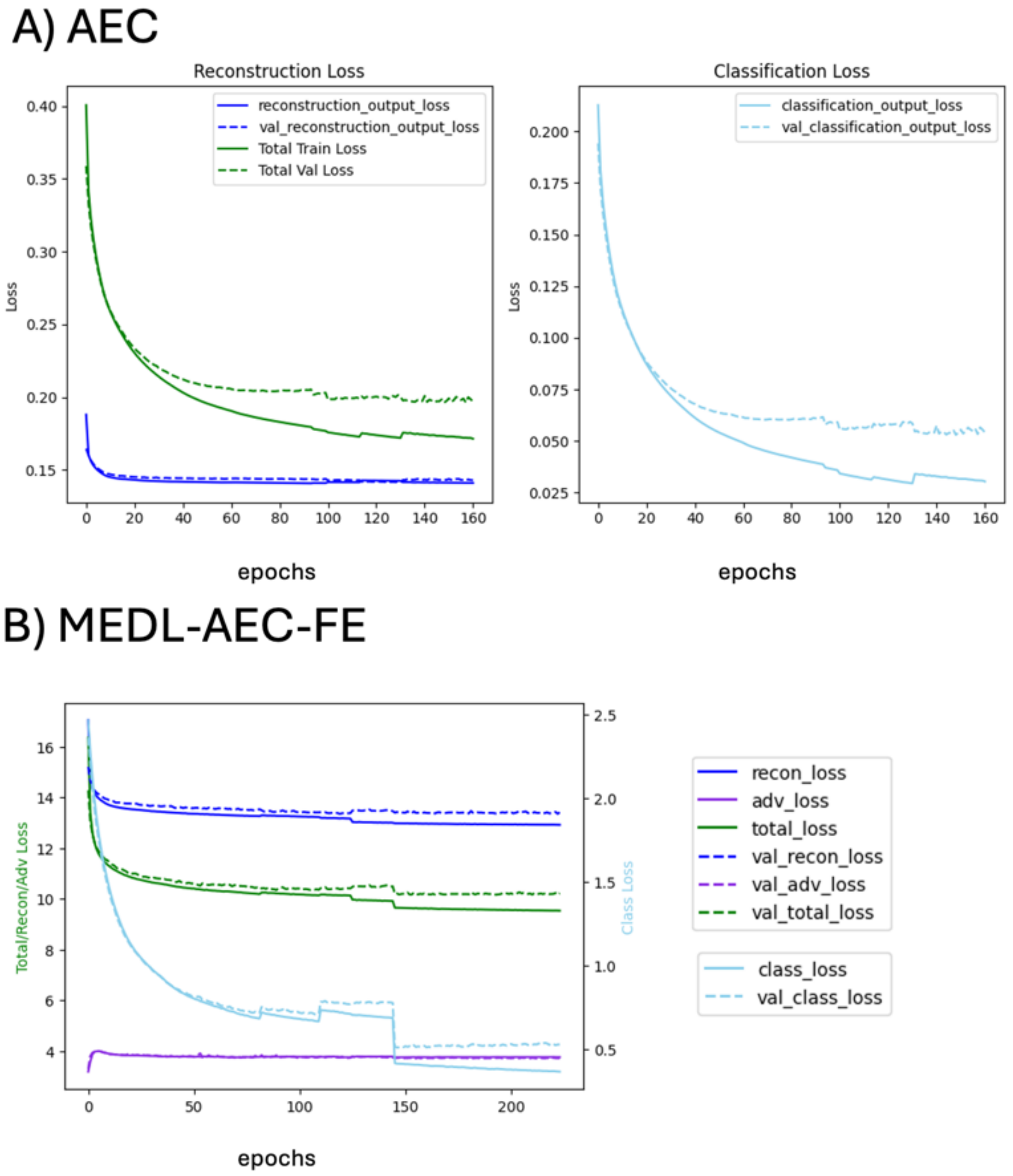

Fig. S8. Training and validation curves for the Healthy Heart dataset for the A) AEC and B) MEDL-AEC-FE models.

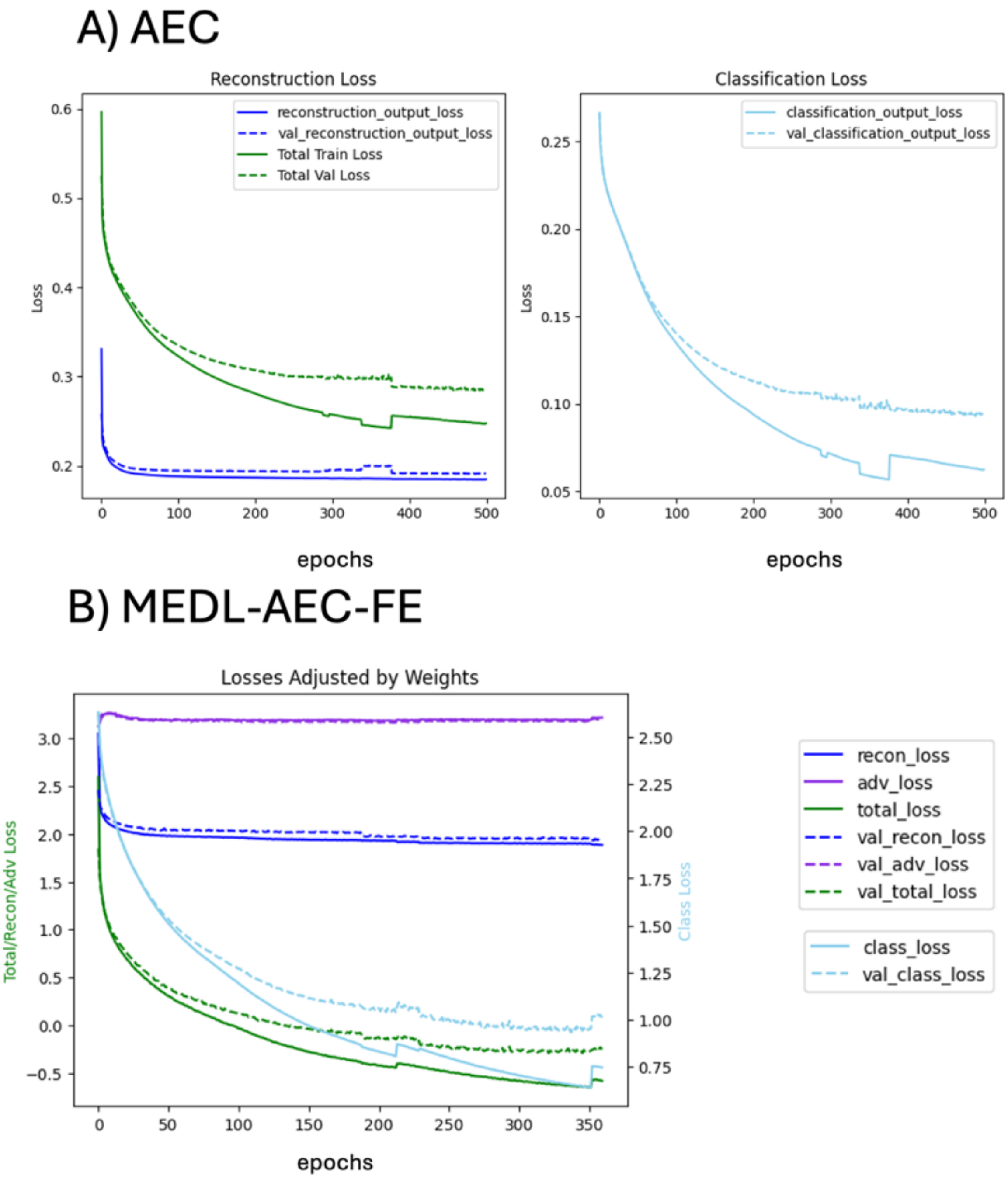

Fig. S9. Training and validation curves for the ASD dataset for the A) AEC and B) MEDL-AEC-FE models.

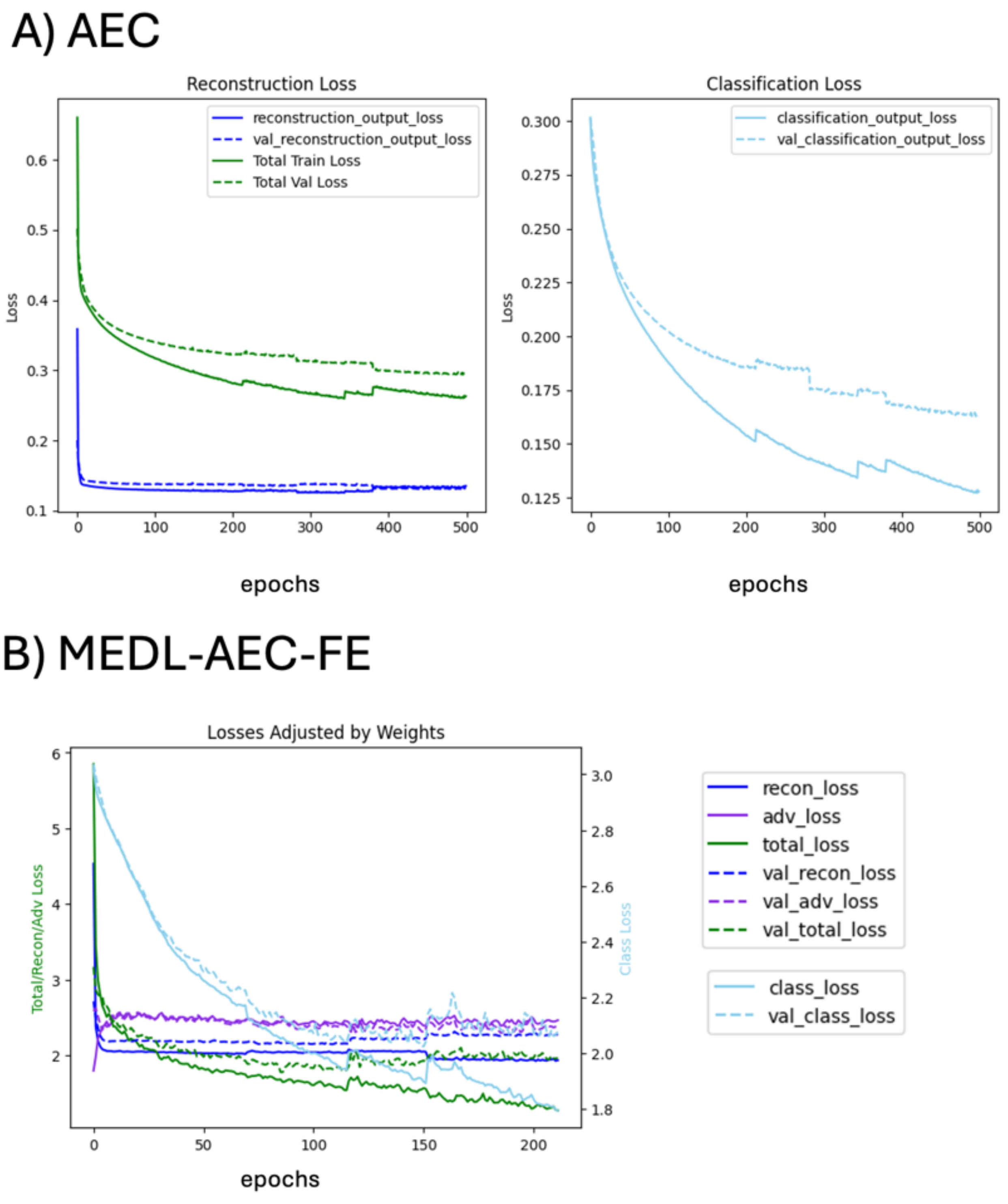

Fig. S10. Training and validation curves for the AML dataset for the A) AEC and B) MEDL-AEC-FE models.

References

Yu, X., Xu, X., Zhang, J., & Li, X. (2023). Batch alignment of single-cell transcriptomics data using deep metric learning. *Nat Commun*, *14*(1), 960. https://doi.org/10.1038/s41467-023-36635-5
